# Galectin-3 associates with NF-κB activation and mitochondrial redox–related protein signatures in catecholamine-induced stress cardiomyopathy

**DOI:** 10.1186/s10020-026-01472-x

**Published:** 2026-04-10

**Authors:** Mana Kalani, Ermir Zulfaj, Erik Axel Andersson, Marion Laudette, Emanuel Fryk, Rossella Crescitelli, Karin Ekström, Per-Anders Jansson, Jan Borén, Elmir Omerovic, Björn Redfors, Vagner Ramon Rodrigues Silva

**Affiliations:** 1 Department of Molecular and Clinical Medicine, Institute of Medicine, Sahlgrenska Academy, University of Gothenburg, Gothenburg, Sweden; 2 Department of Cardiology, Sahlgrenska University Hospital, Region Västra Götaland, Gothenburg, Sweden; 3 Department of Surgery, Institute of Clinical Sciences, Sahlgrenska Center for Cancer Research, Sahlgrenska Academy, University of Gothenburg, Gothenburg, Sweden

**Keywords:** Galectin-3, Extracellular vesicles, Catecholamine-induced cardiac stress, NF-κB activation, Inflammation, Oxidative stress, Mitochondrial remodeling

## Abstract

**Background:**

Acute catecholamine-induced myocardial stress contributes to several cardiac conditions, including takotsubo syndrome (TS), a life-threatening acute cardiac syndrome characterized by transient regional left ventricular dysfunction that lacks evidence-based therapy. Extracellular vesicles (EVs) reflect active cellular stress signaling and enable region-specific analysis of myocardial stress responses to acute injury. Galectin-3 (Gal-3) regulates inflammation and myocardial remodeling and predicts adverse outcomes in myocardial infarction and heart failure, yet its role in catecholamine-driven stress cardiomyopathy, including TS, remains unclear.

**Objectives:**

To characterize regional myocardial stress signaling after catecholamine exposure using cardiac EV proteomics and to determine whether Gal-3–linked stress pathways are associated with the TS-like apical akinesia phenotype.

**Methods and results:**

Catecholamine-induced stress was induced in rats by isoprenaline administration and cardiac function was evaluated by echocardiography. EVs were isolated from apical and basal regions of the left ventricular myocardium using enzymatic digestion, differential centrifugation, and iodixanol purification. EVs were characterized by transmission electron microscopy, western blotting, and nanoparticle tracking analysis. EV protein abundance was quantified using TMTpro 18-plex LC–MS/MS and analyzed for stress and region-specific differences. EV proteomics analysis showed significant enrichment of Gal-3 in EVs isolated from the apical left ventricular myocardium after catecholamine stress and associated Gal-3 with inflammatory (TNF/TLR4-NF-κB-related) and mitochondrial and redox-related signatures. In cardiomyoblasts, recombinant Gal-3 increased NF-κB p65 Ser536 phosphorylation and reduced expression of antioxidant (*Sod3*) and mitochondrial biogenesis related (*Ppargc1a*) transcripts, while inhibition of Gal-3 attenuated TNF-α-induced NF-κB activation and suppressed isoprenaline-induced inflammatory gene expression. *In vivo*, myocardial Gal-3 increased after isoprenaline exposure, but did not differ between animals with or without TS-like apical akinesia, suggesting association with neurohumoral stress exposure rather than the TS-like contractile phenotype.

**Conclusions:**

Gal-3 is upregulated following acute catecholamine exposure and is associated with inflammatory and mitochondrial redox signaling during myocardial stress. Although myocardial Gal-3 increased after isoprenaline exposure it was not associated with the TS-like contractile phenotype, indicating that Gal-3 reflects catecholamine-induced myocardial stress rather than determining regional contractile dysfunction.

**Supplementary Information:**

The online version contains supplementary material available at 10.1186/s10020-026-01472-x.

## Highlights

- Gal-3 is enriched in cardiac extracellular vesicles in a rat model of catecholamine-induced stress cardiomyopathy.
- Gal-3 promotes NF-κB p65 activation and downregulates antioxidant and mitochondrial biogenesis markers, while its inhibition suppresses catecholamine- and TNF-α-induced inflammatory signaling in cardiomyoblasts.
- Gal-3 is upregulated by catecholamine exposure but does not differ between TS and non-TS phenotypes, indicating involvement in the global myocardial stress response rather than in determining the TS-specific contractile pattern.

**Supplementary Information:**

The online version contains supplementary material available at 10.1186/s10020-026-01472-x.

## Introduction

Takotsubo syndrome (TS) is an acute heart failure syndrome that clinically mimics acute coronary syndromes, accounting for approximately 1–3% of suspected acute myocardial infarction presentations. TS is characterized by transient regional left ventricular contractile dysfunction, most often with an apical ballooning pattern on imaging, in the absence of culprit obstructive coronary artery disease (Templin et al., 2015; Prasad et al., 2008). Although the contractile dysfunction typically recovers, TS is associated with significant in-hospital mortality and acute complications. Long-term follow-up reveals persistent low-grade inflammation, diffuse fibrosis, and increased mortality during mid to long term follow up (Ghadri et al., 2018; Scally et al., 2019). Thus, while TS is clinically defined by transient regional wall motion abnormalities, it may be accompanied by broader and more persistent myocardial stress features. Despite extensive investigation, no Takotsubo-specific therapy exists. Furthermore, the molecular responses activated by neurohumoral stress and how they relate to region-specific myocardial contractile dysfunction remain incompletely defined.

A catecholamine surge is widely regarded as the principal trigger of TS, with excess β-adrenergic stimulation contributing to transient regional contractile dysfunction, increased inflammation, oxidative stress related processes and mitochondrial remodelling, and impaired microvascular function (Paur et al., 2012; Pelliccia et al., 2017; Lyon et al., 2021). In experimental models, catecholaminergic drugs reproduce this pattern of abnormalities (Ueyama et al., 2002; Zhao et al., 2019; Zulfaj et al., 2024a). Recent conceptual frameworks have suggested that TS represents an acute stress-response phenomenon in which a surge of sympathetic hormones triggers downstream molecular cascades that lead to transient ventricular stunning (Omerovic and Redfors 2025). However, the specific mediators bridging catecholamine release to localized myocardial contractile dysfunction have not been fully elucidated and remain a major knowledge gap in Takotsubo pathophysiology (Omerovic and Redfors 2025).

Galectin-3 (Gal-3) is a β-galactoside-binding lectin secreted predominantly by macrophages and recognized as a regulator of inflammation, fibrosis, and myocardial remodeling (de Boer et al., 2011; Henderson and Sethi 2009). Elevated Gal-3 levels predict adverse outcomes after myocardial infarction and chronic heart failure and have been incorporated into international heart-failure guidelines (McDonagh et al., 2021; Imran et al., 2017). Mechanistically, Gal-3 promotes leukocyte adhesion, skews macrophage polarization toward a pro-fibrotic phenotype, and activates cardiac fibroblasts (Lok et al., 2013; Sharma et al., 2004). However, whether Gal-3 plays a role in catecholamine-induced stress cardiomyopathy, including TS, remains unknown.

Extracellular vesicles (EVs) are membrane-bound particles secreted by cells that facilitate intercellular communication by transporting a diverse cargo, including proteins, lipids, RNA, and DNA. EVs have recently attracted attention as both mediators and biomarkers in cardiovascular disease, especially under stress conditions where they convey regulatory signals between cardiac cells and regions (Xu et al., 2016; Di Febo et al., 2025). Because EV cargo reflects regulated molecular packaging rather than passive release from injured tissue, EV profiling provides a sensitive readout of active myocardial stress signaling. Recent work using this experimental model demonstrated that cardiac tissue–derived EV proteomics can reveal early molecular signatures of catecholamine-induced myocardial stress, supporting the use of EV profiling to map myocardial stress responses (Zulfaj et al., 2026). In acute catecholamine-driven conditions such as TS, where responses are rapid and regionally heterogeneous, EVs enable detection of early, region-specific signaling. This motivated our use of region-specific cardiac EV proteomics as a hypothesis-generating discovery approach. Preliminary observations suggested that Gal-3 is prominently enriched in EVs derived from cardiac tissue exposed to catecholamine stress, suggesting the use of EV cargo as a discovery platform to map Gal-3-associated stress pathways in the functionally affected versus unaffected myocardium.

In this study, we utilized a rat model of catecholamine-induced stress cardiomyopathy capable of producing a transient TS-like apical ballooning phenotype. We employed region-specific cardiac EV proteomics as an unbiased discovery approach to identify stress-responsive signaling pathways activated in functionally affected versus unaffected myocardium. These analyses revealed Gal-3–associated inflammatory TNF/TLR4–NF-κB and mitochondrial redox signatures enriched in the apical region following catecholamine exposure. We then investigated whether Gal-3 directly modulates these pathways in cardiomyoblasts using gain- and loss-of-function strategies. Finally, we examined whether myocardial Gal-3 expression is associated with the development of the TS-like regional contractile phenotype under identical catecholamine stimulation, thereby distinguishing stress-responsive molecular activation from phenotype-defining determinants.

## Materials and method

### Animals and tissue collection

Transient regional wall motion abnormalities replicating TS was induced in male Sprague–Dawley rats (7–9 weeks old, 300–350 g; Janvier Labs, France) via isoprenaline infusions as previously described (Zulfaj et al., 2024a). Briefly, following one week of acclimatization, rats were anesthetized with ketamine (70 mg/kg) and midazolam (3.5 mg/kg, i.p), given intravenous isoprenaline infusion (1 mg/kg over 15 min) through the tail vein. Animals were sampled across a broader time course including baseline (0 h), 6 h, 24 h, and 30 d after isoprenaline. For the present manuscript, quantitative EV proteomic analyses were restricted to baseline and 24 h samples; 6 h and 30 d samples were used for exploratory western blot assessment of EV-associated Gal-3. Echocardiography was performed using the Vevo 3100 at 6 h to confirm presence of apical ballooning and akinesia and repeated before heart harvesting when applicable. EV and proteomic analysis were performed on hearts from animals that developed apical ballooning. The presence of apical ballooning was defined as apical akinesia of at least 20% of left ventricular endocardial length (left ventricular akinesia index, LVAI). Fractional area change (FAC) and fractional shortening (FS) was measured as previously described (Zulfaj et al., 2025). In a subset of animals, additional hearts were collected at 6 h and 30 days after isoprenaline for exploratory time-course analysis of EV-associated Gal-3.

**Fig. 1:**
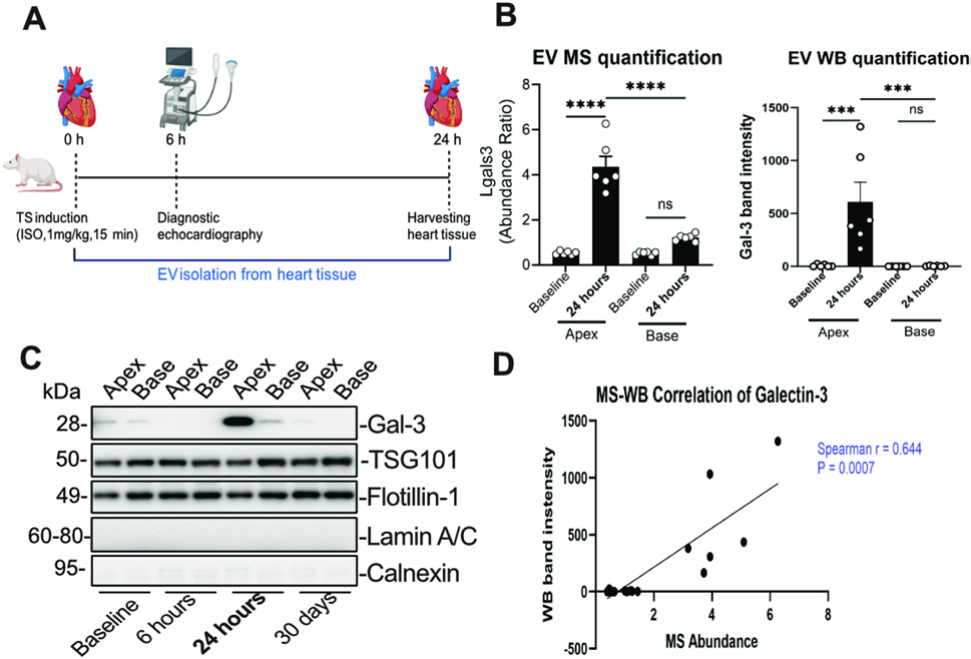
Experimental design and Gal-3 enrichment in cardiac EVs after catecholamine-induced stress in rats. **A** Schematic experimental design. Male rats received intravenous ISO (1 mg/kg, 15 min) to induce catecholamine-mediated cardiac stress. EVs were isolated from apical and basal myocardial regions. Animals were sampled at baseline, 6 h, 24 h, and 30 days after ISO administration. Quantitative EV proteomic analyses in the present study were restricted to baseline and 24 h samples, whereas 6 h and 30 days samples were assessed by western blot as an exploratory time course. **B** Cross-method validation of EV-associated Gal-3 at 24 h. Left: TMTpro LC–MS/MS quantification of EV-associated Gal-3 (*Lgals3*) in EVs isolated from apical and basal myocardium at baseline and 24 h after ISO. Right: Western blot densitometric quantification of EV-associated Gal-3 in the corresponding EV samples. **C** Exploratory western blot time-course analysis of EV-associated Gal-3 in apical and basal myocardial EVs at baseline, 6 h, 24 h, and 30 days after ISO, showing a transient apical peak at 24 h with minimal temporal variation in basal EVs. TSG101 and Flotillin-1 were used as EV markers, while Lamin A/C and Calnexin served as negative controls for nuclear and endoplasmic reticulum contamination. **D** Spearman correlation between LC–MS/MS abundance values and western blot measurements of EV-associated Gal-3 across matched apical and basal samples at baseline and 24 h. Data are presented as mean ± SEM. Data were analyzed using a mixed two-way ANOVA with time (baseline, 24 h) as a between-subject factor and region (apex, base) as a within-subject factor (*n* = 6 hearts per time point), and post hoc comparisons with Benjamini–Hochberg adjustment of p-values *** *p* < 0.001; **** *p* < 0.0001; ns, not significant. ISO = isoprenaline; Gal-3 = Galectin-3; EV = extracellular vesicles; LC–MS/MS = Liquid Chromatography-Tandem Mass Spectrometry; MS = Mass Spectrometry; WB = Western blot

Following euthanasia, hearts were excised, dissected into apex and base regions, rinsed in cold PBS, snap-frozen in liquid nitrogen, and stored at –80 °C for further analysis. Animals were housed 2–3 per cage under controlled conditions (19–21 °C, 12 h light/dark cycle) with ad libitum access to food and water. All procedures were approved by the Gothenburg Animal Ethics Committee (Dnr 5.8.18–02426/2023, renewed approval Dnr. 5.8.18–14,413/2025) and followed national regulations and the ARRIVE guidelines. Figure 1A and Supplementary Fig. S1 show the experimental design for isoprenaline infusion and EV extraction, respectively.

### EV isolation from rat heart tissue

EV isolation was performed as previously reported (Crescitelli et al., 2021), with minor modifications described here. After thawing, the frozen (–80 °C) rat heart tissue (~ 0.2 g per sample) was kept hydrated and placed into a 6-well suspension plate with 2 mL high-glucose DMEM (Gibco). Tissue was minced into ~ 1 mm^3^ fragments using sterile tools. Tissues were treated with Collagenase D (2 mg/mL; Roche) and DNase I (40 U/mL; Qiagen) for (30 min, 37 °C) with gentle agitation in a ThermoMixer (Eppendorf). The digested tissue suspension was filtered through a 70 µm strainer into 50 mL tubes. Wells were rinsed with 1 mL PBS to maximize yield. Filtrate was centrifuged sequentially to remove debris at 300 g (10 min, 4 °C) and 2,000 g (20 min, 4 °C) to remove cells and apoptotic bodies. The remaining liquid was further centrifuged to collect EVs (SW 40 Ti; Beckman Coulter; k-factor 280). Samples were centrifuged at 16,500 g (20 min, 4 °C; large EVs; lEVs) and 118,000 g (2.5 h, 4 °C; small EVs). EV pellets were resuspended in 500 µL PBS and kept on ice.

To further purify EVs, large and small EVs were combined (950 µL) and mixed with 60% (w/v) iodixanol (OptiPrep™, Merck) to achieve a final iodixanol concentration of 45% (w/v). This mixture served as the bottom layer of a discontinuous density gradient. The gradient was completed by sequentially layering 4 mL of 30% and 4 mL of 10% (w/v) iodixanol, followed by approximately 200 µL of PBS on top. Gradients were ultracentrifuged at 186,000 g (2.5 h, 4 °C). EVs were collected between 10–30% iodixanol interface and used for downstream analyses, including western blotting, nanoparticle tracking analysis (NTA), transmission Electron Microscopy (TEM), and proteomics. The EV extraction workflow is shown in Supplementary Fig. S1.

### Nanoparticle tracking analysis

EV samples were characterized for particle concentration and size distribution using a ZetaView® PMX120 instrument (Particle Metrix, Germany). Prior to analysis, the system was calibrated with 100-nm polystyrene beads (Sigma). Each sample was diluted in PBS, typically at ratios between 1:1000 and 1:5000, depending on the initial EV concentration. The diluted suspensions were loaded into the instrument using a syringe, and measurements were performed at eleven positions, with three cycles recorded at each. Data acquisition and processing were carried out using ZetaView® software (version 8.05.11, SP1). Camera sensitivity was set to 80, shutter speed to 100, brightness threshold to 20, and the particle detection range was limited to 10–1000 nm.

### Transmission electron microscopy

EVs were visualized by negative-stain transmission electron microscopy following a procedure adapted from a previously reported protocol (Crescitelli et al., 2021). Approximately 5 µg of purified EVs were deposited onto glow-discharged, 200-mesh copper grids coated with formvar and carbon (Electron Microscopy Sciences). The grids were rinsed twice with distilled water, and the vesicles were fixed using 2.5% glutaraldehyde. After two additional washes, the samples were contrasted with 2% uranyl acetate for (1.5 min). Imaging was performed with a Talos L120C transmission electron microscope (Thermo Fisher Scientific) operating at 120 kV and fitted with a CCD digital camera.

### Mass spectrometry

All apex and base EV protein samples (50 µg) were lysed in 2% sodium dodecyl sulfate (SDS), reduced with 100 mM dithiothreitol (DTT)**,** and alkylated with 10 mM methyl methanethiosulfonate (MMTS)**.** Following acidification with phosphoric acid, proteins were loaded onto S-Trap micro columns (Protifi) in 90% methanol/triethylammonium bicarbonate (TEAB). Digestion was performed overnight at 37 °C with MS-grade trypsin. Peptides were eluted, dried, resolubilized in TEAB, and labeled with TMTpro 18-plex reagents (Thermo Scientific) per manufacturer’s instructions. Samples were pooled, desalted, fractionated by high-pH reversed-phase chromatography, and analyzed by LC–MS/MS on an Orbitrap mass spectrometer using MS^3^-based TMT quantification and data analyzed using Thermo Proteome Discoverer (3.1.0.638) (Cvjetkovic et al., 2024).

### Cell culture

H9c2 rat cardiomyoblasts (ATCC® CRL-1446™) were cultured in high-glucose Dulbecco´s modified Eagle´s medium (DMEM) supplemented with 10% fetal bovine serum, 100 U/mL penicillin and 100 µg/mL streptomycin at 37 °C in a humidified atmosphere of 95% air/5% CO₂. Upon thawing, cells were centrifuged (125 g, 5 min), resuspended in fresh medium, and cultured in T-75 flasks. Medium was changed every 2–3 days, and subculturing was performed at 70–80% confluence using 0.25% trypsin-0.53 mM EDTA (1:4 ratio), with a maximum of six passages to preserve myoblastic properties. Cells were seeded in 12-well plates and grown to ~ 70% confluence and pre-treated, in fresh complete medium, with isoprenaline in dose response (0.1, 1 and 10 µM) for 24 h. The cells were pre-incubated with or without recombinant Gal-3 (Biotechne) and Gal-3 inhibitor GB1107(MedChemTronica;1 µM) for 24 h with or without TNF-α (Biotechne) stimulation for 30 min. Cells not receiving any stimulus were used as a negative control. The cells were cultured for a total of 48 h following pre-incubation and treatment, after which the medium was discarded, and the cells were washed in PBS, followed by lysis for protein measurement or RNA extraction.

### Protein sample preparation

Frozen apex and base regions from rat hearts (24 h post-stress induction), heart-derived EVs, and H9c2 cells were lysed in RIPA buffer (Thermo Fisher Scientific) supplemented with Halt™ protease/phosphatase inhibitor cocktail (Thermo Fisher Scientific). Heart tissue was homogenized using a TissueLyser II (Qiagen) with a 5 mm stainless steel bead (25 Hz, 5 min), followed by centrifugation at 20,000 g (15 min, 4 °C). EV samples were incubated on ice for 5 min before centrifugation at 14,000 g for (10 min, 4 °C). For cells, media were removed, cells were washed with PBS (Ca^2^⁺/Mg^2^⁺-free), lysed with 120 µL RIPA buffer, scraped, vortexed, and centrifuged at 20,000 g (15 min, 4 °C). Protein concentration was measured using the BCA assay kit according to the manufacturer’s instructions (Thermo Fisher Scientific).

### Western blot

Lysed samples were mixed with 4 × Laemmli loading buffer containing β-mercaptoethanol (3:1; Bio-Rad, Cat #1,610,747), boiled at 95 °C for 5 min, and stored at –20 °C. Equal amounts of protein were separated on 10–15% SDS-PAGE gels and transferred to PVDF membranes via wet transfer (100 V, 90 min, 4 °C). Membranes were blocked in 3% bovine serum albumin, and 0.1% tween solution (0.1% Tween-20) for 1 h at room temperature, then incubated overnight at 4 °C with primary antibodies: Galectin-3 (1:1000; Cell Signaling Technology, Cat #89,572), Tubulin α/β (1:1000; Cell Signaling Technology, Cat #2148), Calnexin (1:1000; Abcam, Cat #ab13504, Lot:650,185), Flotillin-1 (1:1000; Abcam, Cat #Ab133497), TSG101 (1:1000; Abcam, Cat #ab125011, clone EPR7130(B)), Lamin A/C (1:1000; Cell Signaling Technology, Cat #2032), p-NFkappaB (1:500; Cell Signaling Technology, Cat #3033), total NFkappaB (1:500; Cell Signaling Technology, Cat #8242) and anti-GAPDH (1:5000; Sigma, Cat #G9545). After washing, membranes were incubated with HRP-conjugated anti-rabbit IgG (1:3000; Cell Signaling Technology, Cat #7074) for 1 h at room temperature. Signals were developed using enhanced chemiluminescence (Merck, Cat #WBKLS0500) and imaged with a ChemiDoc™ system (Bio-Rad). Band intensities were quantified using ImageLab 6.1 and normalized to endogenous control or total protein. In a subset of animals subjected to the same isoprenaline protocol, EVs were also isolated from apical and basal myocardial regions at baseline (0 h), 6 h, 24 h, and 30 days after isoprenaline administration to assess the temporal profile of EV-associated Gal-3 expression.

### RNA extraction and cDNA synthesis

Total RNA was extracted from frozen rat heart tissues (apex/base, ~ 25–35 mg) and H9c2 cells using the RNeasy Mini Kit (Qiagen). RNA purity and concentration were assessed via NanoDrop (Thermo Fisher Scientific); only samples with A260/A280 ratios of 1.7–2.0 were used. cDNA synthesis was performed using the SuperScript™ VILO™ cDNA Synthesis Kit (Thermo Fisher Scientific, Cat #11,754–050) according to the manufacturer’s instructions.

### Quantitative Real-Time PCR

Quantitative Real-Time PCR (RT-qPCR) was performed using the ViiA™ 7 Real-Time PCR System (Applied Biosystems) with SsoAdvanced™ Universal Inhibitor-Tolerant SYBR® Green Supermix (Bio-Rad, Cat #1,725,274). Each 20 µL reaction contained 2 µL of cDNA, 10 µL of SYBR Green Supermix, 0.5 µL each of forward and reverse primers, and nuclease-free water. Amplification was carried out according to the manufacturer's protocol, followed by melt curve analysis to confirm reaction specificity. Relative gene expression was calculated using the ΔΔCt method, normalized to GAPDH as the internal control. Primer sequences are shown in Supplementary Table S1.

### Statistics

For EV proteomics, quantitative analysis focused on differences in EV protein abundance between apex and base at baseline and 24 h after isoprenaline. The TMT-based EV proteomic data from heart regions were log₂-transformed and normalized prior to analysis. Differential expression was assessed using the *limma* package in R (v4.2.0) with empirical Bayes moderation; proteins with |log₂FC|> 0.58 and adjusted *P* < 0.05 (Benjamini-Hochberg) were considered significant. Heatmaps of protein expression data were generated using Z-score normalization. Hierarchical clustering was performed with the average linkage method, utilizing Euclidean distance for row clustering and correlation distance for column clustering. Over representation analysis (ORA) was performed on DEPs with enrichment analysis using the clusterProfiler (4.10.1) by querying the Gene Ontology database (GO, BP; Biological Processes). Gene set enrichment analysis (GSEA) was performed by querying the GO-BP. Protein–protein interaction analysis was conducted in Cytoscape, with interactions enriched through the STRING database. Co-expression between *Lgals3* and all detected proteins was evaluated using Spearman correlation. Redox-specific networks were filtered by ROS-related functions (Reactome/GO) and visualized in Cytoscape. Single comparisons between groups on continuous variables were calculated using the Mann–Whitney U test. Multiple group comparisons were analyzed using one and two-way ANOVA followed by appropriate post hoc tests (Benjamini–Hochberg adjusted comparisons) in GraphPad Prism v10.4.1. Correlations between variables were assessed using Spearman rank correlation analysis, including evaluation of the relationship between mass spectrometry–derived Galectin-3 abundance and normalized western blot band intensity across EV samples. A *P* value < 0.05 was considered statistically significant. Additional figures were created using Biorender.

## Results

### Galectin-3 increases in EVs after catecholamine-induced cardiac stress

To capture region-specific myocardial stress signaling rather than bulk tissue composition, we performed quantitative proteomic profiling of EV cargo from the apical and basal myocardium at baseline and 24 h after isoprenaline exposure (Fig. 1A). This regional design was motivated by the preferential involvement of the left ventricular apex and enabled comparison of EV cargo between functionally affected apical and unaffected basal myocardium during early stress. Within this dataset, EV-associated Gal-3 (*Lgals3*) was markedly increased in apical EVs at 24 h compared with both apical baseline and the corresponding basal region (Fig. 1B). Gal-3 ranked among the top 30 enriched proteins (Supplementary Table S2), supporting its prominence in the apical EV response. Western blot analysis showed a similar pattern and demonstrated that apical EV-associated Gal-3 was low at baseline and 6 h, peaked at 24 h, and returned toward baseline by 30 d, whereas basal EVs showed minimal temporal variation (Fig. 1C). Consistent with this, mass spectrometry and western blot measurements of EV-associated Gal-3 were positively correlated across matched samples (Fig. 1D). EV identity and purity were supported by detection of canonical EV markers (TSG101, Flotillin-1), absence of the nuclear endoplasmic Lamin A/C and Calnexin, and complementary characterization with nanoparticle tracking analysis (NTA) and transmission electron microscopy (TEM) (Supplementary Figs. S2 and S3). Together, these findings identify a transient enrichment of Gal-3 in apical EVs at 24 h as a prominent early response to catecholamine stress and provide the rationale for focusing subsequent EV proteomic network analyses on this time point.

### Gal-3 associated EV proteome reveals inflammatory and remodeling pathways after catecholamine-induced cardiac stress

**Fig. 2:**
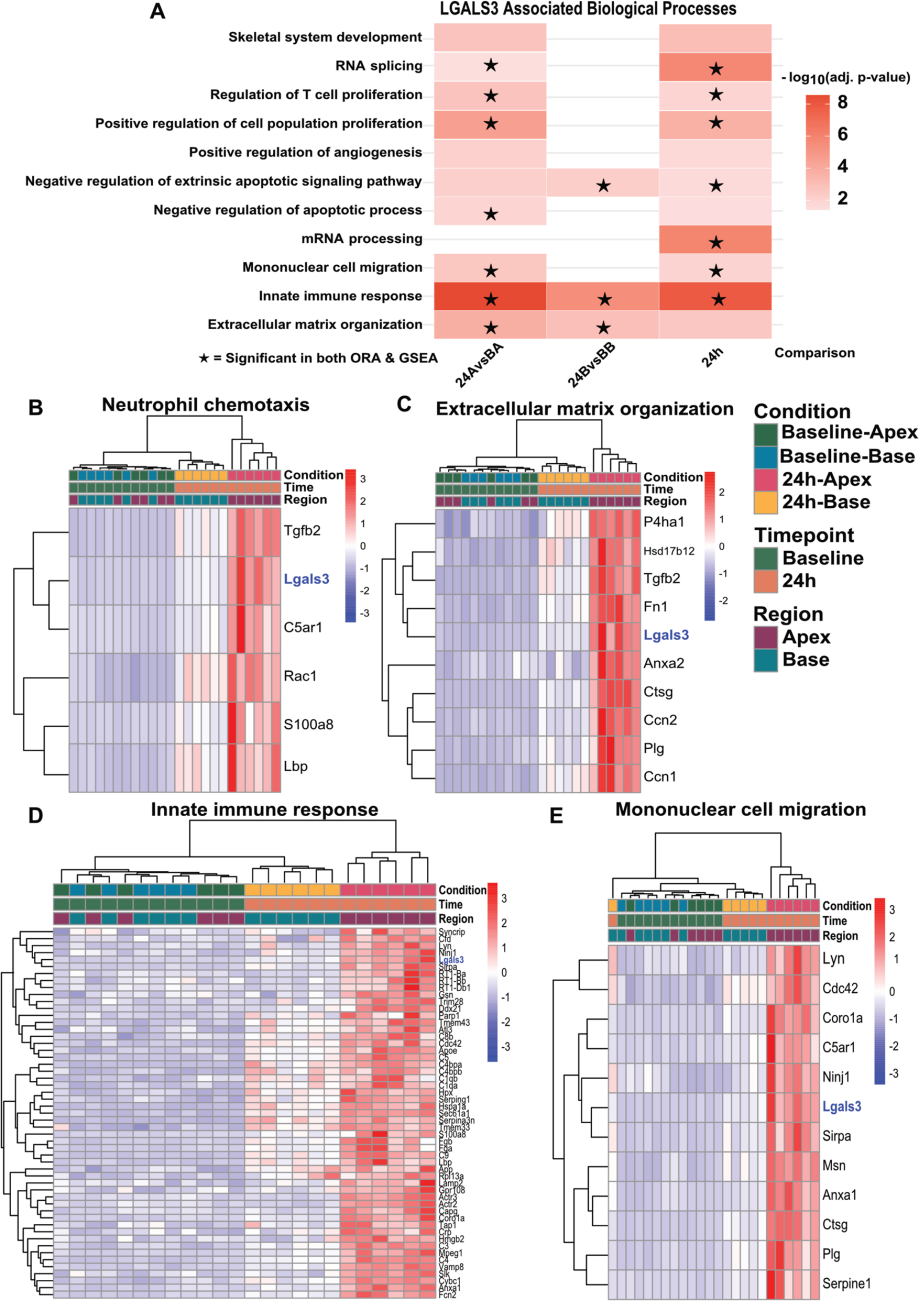
Gal-3 associated EVs signatures following catecholamine-induced stress in rats. **A** Significant *Lgals3*-associated biological processes in Over-Representation Analysis (ORA) and Gene Set Enrichment Analysis (GSEA). 24 A vs BA; 24-h apex vs Baseline Apex. 24B vs BB; 24-h base vs baseline base. 24 h; 24-h apex vs 24-h base. Asterisks denote significant processes in both ORA and GSEA. Specific heat-maps of all proteins in the Gene Ontology term (**B**) Neutrophil chemotaxis; **C** Extracellular matrix organization; **D** Innate immune response; **E** Mononuclear cell migration. Ordered by hierarchical clustering; columns = Baseline-Apex, Baseline-Base, 24 h-Apex, 24 h-Base. *Lgals3*-highlighted in blue

To guide subsequent functional studies, we applied Over-Representation Analysis (ORA) and Gene Set Enrichment Analysis (GSEA) to the 24 h EV proteome in the apex and base region after catecholamine-induced stress. The analyses focused on biological processes that contain *Lgals3*, to translate the peak Gal-3-linked protein changes into pathway-level insights. In the apical region, 315 proteins were upregulated compared with the 24-h basal samples, and 586 proteins were upregulated compared with apical baseline (|FC|> 0.58, adj.P < 0.05), ORA highlighted innate immune response (GO:0045087, FDR = 3.3 × 10^–7^), and mononuclear cell migration (GO:0071674, FDR = 0.004). GSEA corroborated this and further showed a positive enrichment in neutrophil chemotaxis (NES = 1.7, FDR = 0.003) and extracellular matrix organization (NES = 1.8, FDR = 2.1 × 10^–5^) gene sets (Fig. 2A–E). Heatmap visualization revealed distinct expression clusters across enriched pathways. In the neutrophil chemotaxis module (Fig. 2B), *Lgals3*, *Tgfβ2*, and *C5aR1* were prominently upregulated in apical EVs, consistent with enrichment of innate immune–related protein signatures. The extracellular matrix organization cluster (Fig. 2C) showed coordinated up-regulation of Gal-3 with matrix proteins (*Fn1*, *P4ha1*, *Anxa2*), consistent with enrichment of extracellular matrix–associated proteins commonly linked to fibrotic remodeling processes, including matrix organization and collagen-processing pathways. In parallel, the innate immune response module (Fig. 2D) displayed broad activation of cytokine and complement components, while the mononuclear cell migration cluster (Fig. 2E) showed co-expression of *Lgals3*, *C5aR1*, and *NinJ1*, suggesting recruitment of inflammatory cells. These findings suggest that Gal-3 associated EV signatures are enriched for inflammatory and extracellular matrix related processes, indicating a close association between Gal-3 and immune- and remodeling-related protein networks during catecholamine-induced myocardial stress.

### Gal-3 associated EV networks reveal coordinated inflammatory signaling.

**Fig. 3:**
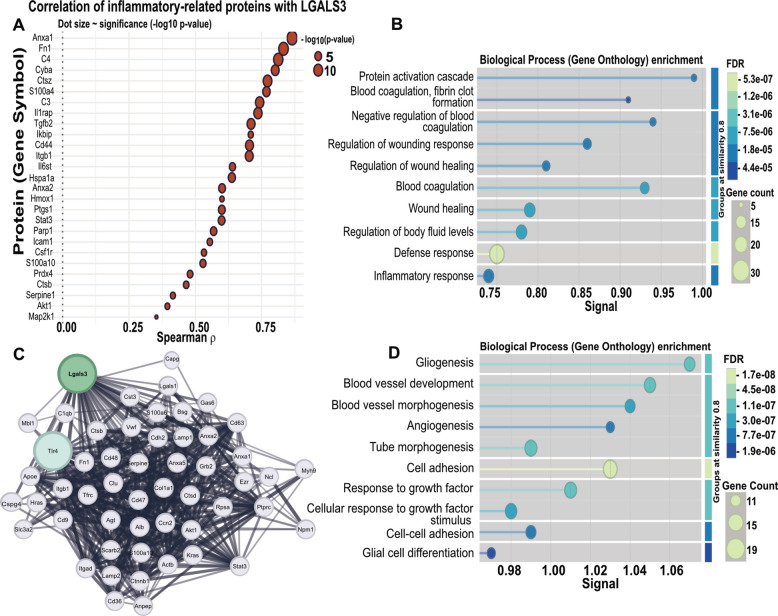
Gal-3-centered EV protein networks link to early innate-immune and pro-fibrotic signaling after catecholamine-induced stress in rats. **A** Spearman correlation between *Lgals3* and inflammation- related proteins (adj. *p*<0.05). Dot size reflects the –log10 FDR. **B** Top Gene-Ontology biological-process terms enriched among the positively correlated proteins. Processes related to coagulation, wound healing and inflammation predominate. Node size represents gene count and color reflects the FDR. Terms are arranged on the y-axis by semantic similarity (clustered at 0.8 similarity threshold) and along the x-axis by their enrichment score (signal = −log10 FDR × gene count). **C **STRING protein–protein-interaction network seeded on Gal-3. Edge width and opacity denote STRING combined-score confidence. Immune-signaling nodes (e.g., TLR4) cluster around Gal-3, highlighting a densely connected inflammatory interaction network centered on Gal-3. **D** GO enrichment map of the STRING network, depicting significantly over-represented terms (FDR<0.05); angiogenesis, tube morphogenesis and cell-adhesion categories are highlighted, indicating pro-fibrotic and vascular-remodeling pathways.

Having identified innate-immune and pro-fibrotic pathways as the dominant 24 h signals, we next examined which EV proteins co-varied with Gal-3. Correlation analysis revealed that *Lgals3* showed strong positive associations with multiple inflammation-related proteins, including *Fn1*, *Anxa1*, *C3*, *S100a8*, *Tgfβ2*, and *C5aR1* (Fig. 3A and Supplementary Appendix). Many of these proteins mediate immune activation and tissue remodeling: *S100a8* and *C5aR1* promote neutrophil recruitment, *Tgfβ2* drives fibroblast activation, and *Fn1* and *Anxa1* support matrix stabilization and repair. Functional enrichment analysis of EV proteomics–derived proteins that were correlated positively with *Lgals3* revealed overrepresentation of biological processes related to inflammatory response, coagulation, wound healing, and vascular homeostasis (Fig. 3B). These processes reflect coordinated activation of innate immunity and early myocardial repair. A STRING-derived protein–protein interaction (PPI) network placed on Gal-3 as the center of a densely interconnected inflammatory module that included *Tlr4*, *Fn1*, *C3*, and *S100a9* (Fig. 3C). Notably, *Tlr4* is a well-established regulator of catecholamine-induced myocardial inflammation, suggesting a mechanistic link between Gal-3 signaling and toll-like receptor activation (Kawai and Akira 2007). Gene Ontology enrichment of the same PPI highlighted biological processes associated with angiogenesis, tube morphogenesis, and cell adhesion (Fig. 3D), pointing to concurrent initiation of vascular repair and extracellular matrix remodeling. Taken together, the Gal-3-centered correlations, 24 h-apical enrichment, and the STRING PPI network are consistent with a role for Gal-3 in inflammatory and reparative signaling during the early myocardial response to catecholamine stress.

### Gal-3 contributes to TNF-induced NF-κB activation

Three complementary layers of our EV-proteomics analysis (pathway enrichment, protein co-variation, and STRING interaction mapping) converged on Gal-3 and the TNF/TLR4- > NF-κB inflammatory axis. Based on the central role of NF-κB downstream of TNF/TLR signaling in regulating cardiac inflammatory stress responses, this pathway was examined as a candidate mediator linking Gal-3 to catecholamine-induced signaling. To move from correlation to mechanism, and to establish a functional link, we investigated whether Gal-3 modulates this pathway in cardiomyoblasts. Specifically, we examined the effect of catecholaminergic stimulation on Gal-3 expression, assessed the ability of Gal-3 to independently trigger canonical NF-κB signaling, and determined the necessity of Gal-3 for maximal NF-κB activation following TNF-α stimulation. These mechanisms were examined using a rat cardiomyoblast model exposed to isoprenaline to mimic catecholamine surges, recombinant Gal-3 (rGal-3) to assess gain-of-function effects, and the selective Gal-3 inhibitor GB1107 to evaluate loss-of-function responses.

**Fig. 4:**
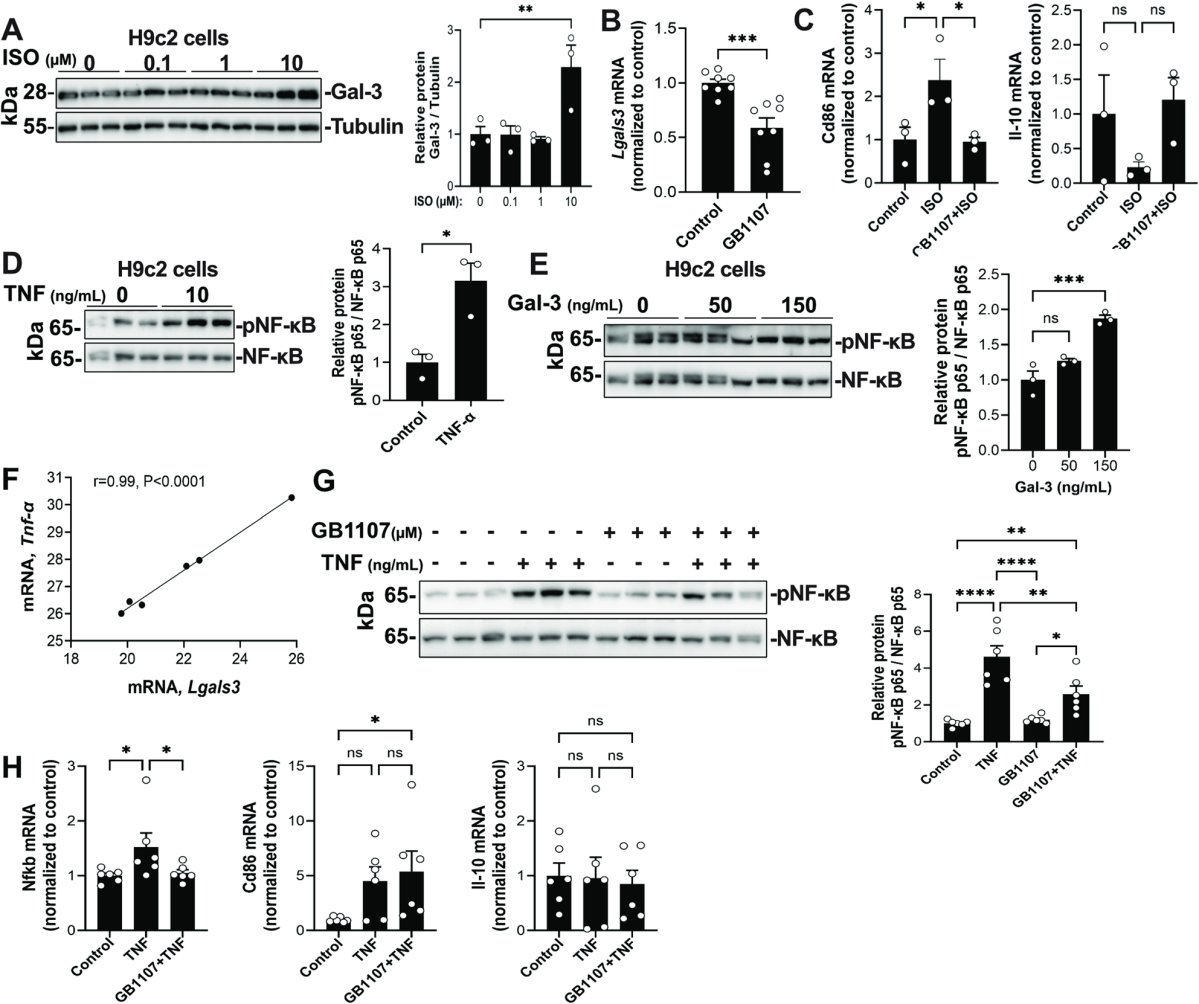
Gal-3 contributes to TNF-α induced NF-κB activation and inflammatory gene expression in cardiomyoblasts. **A** Western blot quantifications of Gal-3 after stimulation with isoprenaline (ISO) (0, 0.1, 1 and 10 μM) in H9c2 cells for 24 h (*n* = 3 per group and per time point). **B**-**C** mRNA expression analysis in H9c2 cells after treatment with GB1107 (1 μM) with or without ISO (*n* = 8 in panel B; *n* = 3 in panel (**C**). GB1107 was added 24 h prior to adding ISO and ISO, when indicated, was added for 24 h. **D**-**E** Analysis of NF-κB p65 phosphorylation at Ser536 and western blot quantification in H9c2 cells after recombinant TNF-α (rTNF-α; 10 ng/mL) or recombinant Gal-3 (rGal-3; 0, 50 and 100 ng/mL) administered for 30 min (*n* = 3 per group). **F** Spearman correlation analysis between *Tnf* mRNA levels and *Lgals3* mRNA levels in rats submitted to catecholamine-induced stress (*n* = 6, 3 apex and 3 base region). **G** Analysis of NF-κB p65 phosphorylation at Ser536 and western blot quantifications. **H** mRNA expression analysis after rTNF-α (10 ng/mL) individually or in combination with GB1107 (1 μM) in H9c2 cells. GB1107 was added 24 h before rTNF-α and rTNF-α was added for 30 min (*n* = 6). Control cells received no stimulus. Data are presented as mean ± SEM or Spearman correlation coefficients. ***p < 0.001; **p < 0.01 by Mann–Whitney test (**B** and **D**). *p < 0.05; **p < 0.01; ***p < 0.01; ****p < 0.0001 by one-way ANOVA (**C**, **E**, **G**, and **H**), ns = non-significant

*In vitro*, treatment of H9c2 cardiomyoblasts with isoprenaline (10 μM) for 24 h significantly induced Gal-3 expression (Fig. 4A). *In vivo*, isoprenaline infusion in rats resulted in a significant increase in Gal-3 protein and mRNA levels in the apical myocardium at 24 h compared with both the basal region and baseline group (Supplementary Fig. S4C). No significant differences were observed between the base region and the baseline group.

Since it is well established that catecholamine-induced stress activates inflammatory signaling pathways, including TNF-α/NF-κB signaling (Lyon et al., 2008), we examined whether inhibition of Gal-3 could suppress the inflammatory response in cardiomyoblasts. Treatment with GB1107 reduced *Lgals3* gene expression after 24 h (Fig. 4B). Notably, GB1107 also suppressed isoprenaline-induced inflammatory gene expression. CD86, an immune activation marker typically expressed on antigen-presenting cells, was used here as a marker of inflammatory activation in cardiomyoblasts (Fig. 4C). Consistent with activation of inflammatory signaling pathways, It is well established that TLR signaling converges on NF-κB activation, which regulates the expression of numerous inflammatory cytokine genes (Kawai and Akira 2007). Moreover, TNF-α promotes NF-κB p65 phosphorylation on Ser536 (Hayden and Ghosh 2014). We confirmed this by showing that treatment with 10 ng/mL of TNF-α for 30 min significantly increased NF-κB p65 phosphorylation (Fig. 4D).

We investigated whether Gal-3 could modulate NF-κB pathway activation in the context of spatially localized stress signaling during catecholamine-induced cardiac stress. Indeed, a dose-dependent increase in NF-κB p65 phosphorylation at Ser536 was observed following treatment with recombinant Gal-3, reaching significance at 150 ng/mL (Fig. 4E). Interestingly, *Lgals3* mRNA levels exhibited a strong positive correlation with *Tnf-α* expression (*r* = 0.99, *P* < 0.0001) in cardiac tissue, suggesting a close association between Gal-3 expression and the TNF-α/NF-κB axis (Fig. 4F). Notably, while TNF-α alone robustly induced NF-κB p65 phosphorylation and downstream gene expression, this effect was significantly reduced by GB1107 (Fig. 4G). However, Gal-3 inhibition did not affect TNF-α–induced *Cd86,* a marker of pro-inflammatory immune activation, and *Il-10,* an anti-inflammatory cytokine, gene expression (Fig. 4H).

Taken together, our data indicate that Gal-3 can independently activate NF-κB signaling and contributes to TNF-α–induced NF-κB activation under these experimental conditions.

### Gal-3 is associated with changes in mitochondrial-related oxidative stress

**Fig. 5:**
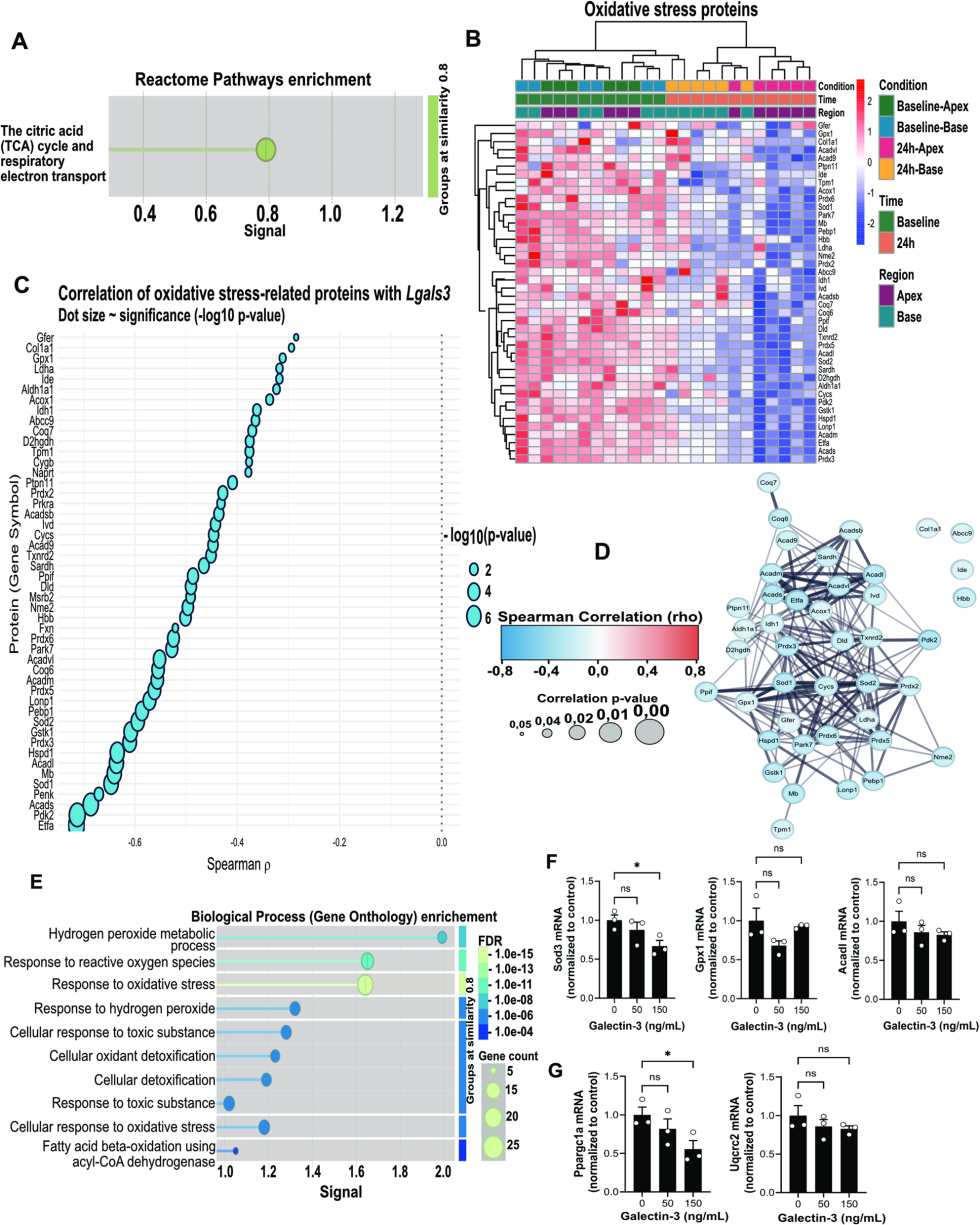
Gal-3 associates with mitochondrial oxidative stress–related protein signatures after catecholamine challenge.** A** Functional enrichment of significantly negatively correlated EV proteins with *Lgals3*. Significant Reactome pathway enrichment related to the citric acid (TCA) cycle and respiratory electron transport (FDR <0.05). **B** Heat-map of all proteins in the GO term “response to oxidative stress,” ordered by hierarchical clustering; columns = Baseline-Apex, Baseline-Base, 24 hours-Apex, 24 hours-Base. **C** Bubble plot of the same oxidative-stress proteins plotted against *Lgals3* abundance. X-axis, Spearman ρ; bubble size, –log10(p-value). **D** STRING protein-protein interaction network of oxidative-stress proteins that are negatively correlated with Gal-3. Edge width and opacity scale with STRING combined confidence score. **E** Network enrichment highlights proteins annotated to mitochondrial TCA cycle, β-oxidation and ROS-detoxification pathways (FDR < 0.05). **F-G** mRNA expression analysis after recombinant Gal-3 (rGal-3; 0, 50 and 150 ng/mL) treatment for 24 hours in H9c2 cells (n=3 per time point). Data are presented as mean ± SEM. *p<0.05 by one-way ANOVA, ns=non-significant.

Correlation analysis (Supplementary Appendix) identified 388 proteins that showed a significant negative correlation with *Lgals3* (Spearman ρ < 0, *p* < 0.05). Reactome pathway analysis revealed that these proteins were enriched in the tricarboxylic acid (TCA) cycle and respiratory electron transport pathways, suggesting a link between Gal-3 and mitochondrial oxidative metabolism (Fig. 5A). These pathways are central to mitochondrial ATP production but are also major sources of intracellular reactive oxygen species (ROS). These data suggest that Gal-3 upregulation coincides with altered abundance of EV-associated proteins linked to mitochondrial metabolism and redox-related pathways. We therefore investigated redox-related profiles in the EV dataset and validated key findings in H9c2 cardiomyoblasts.

An overview heat-map of EV-associated proteins annotated to the Gene Ontology term “response to oxidative stress” revealed a coordinated down-regulation specifically confined to the 24 h apical samples (Fig. 5B). This suppression involved multiple mitochondrial and cytosolic antioxidant enzymes, including *Prdx2*, *Gpx4*, *Sod2*, and *Txn2*, together with components of fatty-acid β-oxidation such as *Acadl*. Plotting these proteins against Gal-3 abundance revealed significant inverse correlations, indicating that higher Gal-3 levels coincide with reduced expression of core redox and metabolic regulators (Fig. 5C). When the negatively correlated set was projected onto a STRING protein–protein-interaction map, the proteins formed a single, densely connected network whose enrichment pointed to β-oxidation and ROS-detoxification pathways (Fig. 5D and E). This network structure suggests that Gal-3 is associated with proteins involved in mitochondrial oxidative activity and antioxidant pathways. Collectively, these data suggest that the Gal-3 surge following catecholamine stress is associated with coordinated changes in EV-associated mitochondrial antioxidant and metabolic proteins in the apical myocardium. Mitochondrial ROS production was not directly assessed in these experiments.

To test whether Gal-3 can directly reproduce the redox signature uncovered in the EV dataset, we exposed H9c2 cardiomyoblasts to recombinant Gal-3 (rGal-3; 50 and 150 ng/mL) for 24 h and quantified representative antioxidant and mitochondrial transcripts. rGal-3 lowered *Sod3* mRNA in a dose-dependent manner, reaching ~ 40% repression at 150 ng/mL, whereas neither *Gpx1* nor the β-oxidation enzyme *Acadl* changed significantly (Fig. 5F). The same concentration of rGal-3 also reduced *Ppargc1a,* the master regulator of mitochondrial biogenesis, without significantly affecting the respiratory‐chain subunit *Uqcrc2* (Fig. 5G).

Thus Gal-3 treatment reduced expression of selected antioxidant (*Sod3*) and mitochondrial biogenesis related (*Ppargc1a*) transcripts, indicating that Gal-3 can modulate components of redox and mitochondrial-associated gene programs in cardiomyoblasts. Notably, NF-κB (p65) phosphorylation and TNF-α were modestly elevated, consistent with a limited inflammatory response.

### Gal-3 is not associated with catecholamine-induced myocardial dysfunction

**Fig. 6:**
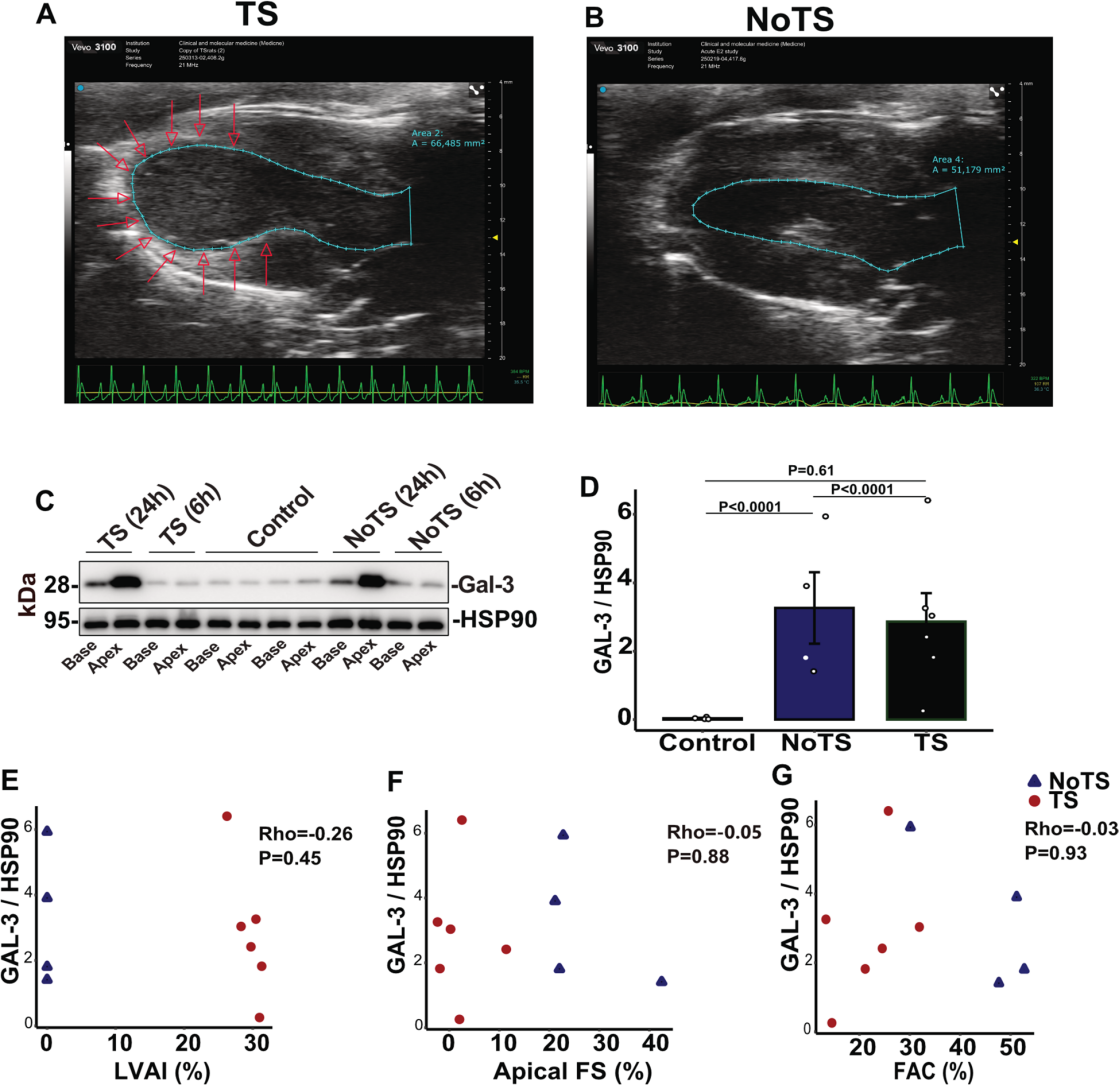
Gal-3 expression is not significantly associated with takotsubo-like phenotype after isoprenaline infusion. Representative parasternal long-axis echocardiographic images at end-systole 6 h after isoprenaline infusion showing Takotsubo-like apical ballooning (TS; **A**) or preserved apical contraction (NoTS; **B**). Endocardial borders are traced (cyan), and the akinetic apical segment in TS is indicated by arrows. **C** Representative Western blot of Gal-3 in myocardial samples from control, TS, and NoTS rats at 6 h and 24 h after isoprenaline. **D** Quantification of Gal-3 protein levels in apical myocardium, normalized to HSP90, in control, NoTS, and TS groups at 24 h. Bars represent mean ± SEM. *p < 0.05 by one-way ANOVA with Benjamini–Hochberg adjustment for multiple comparisons (*n* = 4, 4, 6). Spearman correlation coefficient (**E–G**). Scatter plots showing the relationship between apical Gal-3/HSP90 and indices of left ventricular function at 24 h, left ventricular akinesia index (**E**), apical fractional shortening (**F**), and fractional area change (**G**)

To determine whether Gal-3 associates with the TS-like phenotype, we compared animals that developed apical akinesia with those that did not following identical isoprenaline exposure (Fig. 6A–B). Isoprenaline induced a marked increase in myocardial Gal-3, peaking at 24 h (Fig. 6C). Apical Gal-3 levels were elevated in both animals that did or did not develop the TS-like phenotype relative to controls (Fig. 6D). Furthermore, Gal-3 abundance did not differ between TS and non-TS groups and did not correlate with left ventricular akinesia index, fractional shortening, or fractional area change (Fig. 6E–G). Thus, Gal-3 upregulation was observed following catecholamine exposure but was not associated with the TS-like contractile phenotype. These data indicate that while Gal-3 is embedded within the inflammatory and redox programs activated during catecholamine challenge, it does not determine the emergence of transient apical dysfunction. Rather, Gal-3 appears to reflect activation of the catecholamine-induced myocardial stress response. The dissociation between molecular stress activation and regional wall motion abnormality implies that additional region-specific mechanisms govern the TS-like pattern.

## Discussion

In this study, we show that Gal-3 is enriched in EVs derived from the apical myocardium compared with basal regions 24 h after catecholamine-induced stress. This enrichment is associated with EV-derived protein networks related to immune activation, redox regulation, and extracellular matrix remodeling. In complementary mechanistic experiments, Gal-3 promoted NF-κB p65 activation and reduced antioxidant and mitochondrial biogenesis markers in cardiomyoblasts, while pharmacological Gal-3 inhibition attenuated catecholamine- and TNF-α-induced inflammatory signaling. Despite this robust association with stress-related molecular programs, myocardial Gal-3 abundance did not differ between rats with and without TS-like apical akinesia after isoprenaline and did not correlate with indices of global or regional left ventricular function. Taken together, these findings suggest that Gal-3 predominantly reflects and modulates the myocardial stress-response axis rather than determining the TS-specific contractile phenotype. 

Our EV data indicates that Gal-3 is embedded in a broader program of innate immune activation and extracellular matrix-associated signaling in the catecholamine-stressed apex. Although isoprenaline is delivered to the whole body, the heart does not respond uniformly, and the apical myocardium is more susceptible to catecholamine-induced stress than the basal region. Gal-3-associated EV signatures were enriched for processes related to leukocyte recruitment, complement and coagulation cascades, and matrix organization, together pointing to coordinated activation of inflammatory and reparative pathways. These extracellular matrix signatures likely represent early remodeling-related signaling rather than overt fibrosis, which is consistent with the acute time frame of this model (Zulfaj et al., 2024a). Gal-3 is a pleiotropic lectin expressed by multiple cardiac cell types and plays a well-established role in inflammatory signaling and extracellular matrix remodeling in heart failure and after myocardial infarction (de Boer et al., 2011; Henderson and Sethi 2009; Sharma et al., 2004; Calvier et al., 2015).

By capturing proteins actively released into the extracellular space, EV proteomics provides a complementary perspective to conventional tissue proteomics, emphasizing the signaling component of the cardiac stress response (Xu et al., 2016; Crescitelli et al., 2021; Cvjetkovic et al., 2024). These findings extend previous work demonstrating that cardiac tissue–derived EV proteomics can capture early molecular signatures of catecholamine-induced myocardial stress in this experimental model (Zulfaj et al., 2026). Our findings suggest that Gal-3 enriched EV signatures reflect regionally active inflammatory and remodeling signaling within the stressed myocardium. Because EVs were isolated from whole myocardial regions, the cellular origin of Gal-3–enriched EVs cannot be definitively assigned and likely reflects contributions from multiple cardiac cell types (Henderson and Sethi 2009; Sharma et al., 2004; Xu et al., 2016; Crescitelli et al., 2021).

Multiple layers of analysis converged on a tight link between Gal-3 and the TNF/TLR4-NF-κB axis. Although catecholamines do not directly activate TNF/TLR4 signaling, excessive β-adrenergic stimulation is known to induce myocardial oxidative and inflammatory stress, which secondarily engages TNF/TLR4–NF-κB–dependent pathways (Scally et al., 2019; Paur et al., 2012; Lyon et al., 2008; Hayden and Ghosh 2014). Proteins that clustered with Gal-3 in EVs were enriched for pathways typically driven by pattern-recognition receptors and pro-inflammatory cytokines, and network analysis placed Gal-3 within a dense inflammatory interaction module that included TLR4 and complement components (Zhou et al., 2018; Wang et al., 2025).

These observations fit well with the previous reports that Gal-3 can amplify the TLR4/MyD88/NF-κB signaling and that pharmacological Gal-3 inhibition reduces inflammatory injury in cardiovascular and inflammatory models (Xu et al., 2020; Sun et al., 2021). Our *in vitro* experiments extend these concepts to cardiomyoblasts, showing that recombinant Gal-3 is sufficient to increase NF-κB p65 phosphorylation and that the selective inhibitor GB1107 dampens TNF-α-induced NF-κB activation and inflammatory gene expression. Together, these findings support a model in which Gal-3 is not simply a bystander marker of inflammation but contributes to shaping NF-κB-dependent signaling in the context of catecholamine-induced stress.

A second major theme emerging from our data is the association between Gal-3 and mitochondrial redox remodeling. These mechanistic experiments were used to examine whether Gal-3 could modulate pathways highlighted by the EV proteomic analysis.

Proteins that were inversely related to Gal-3 in EVs formed a coherent network enriched for TCA-cycle enzymes, respiratory-chain components, β-oxidation pathways and antioxidant defenses. Apical samples 24 h after catecholamine challenge displayed a coordinated reduction in mitochondrial antioxidant enzymes, and *in vitro* Gal-3 exposure reduced expression of *Sod3* and the mitochondrial biogenesis regulator *Ppargc1a*. Although mitochondrial function and ROS were not directly assessed, these findings are consistent with Gal-3 being linked to redox- and mitochondria-related stress signaling programs. Similar Gal-3-associated changes in redox and mitochondrial regulation have been described in other organs and disease settings (de Boer et al., 2011; Henderson and Sethi 2009; Sharma et al., 2004; Calvier et al., 2015), suggesting that related stress programs may also be engaged in acute stress cardiomyopathy, although their relationship to contractile phenotype appears more complex.

One of the most informative findings of this study is the dissociation between Gal-3 induction and the TS-like contractile phenotype. Despite clear evidence that Gal-3 is induced by catecholamine stress in EVs, myocardium, and cardiomyoblasts, myocardial Gal-3 levels did not differ between rats with and without TS-like apical akinesia and did not correlate with global or regional systolic echocardiographic indices. This finding argues against Gal-3 as a primary driver of transient wall motion abnormalities and instead supports a role for Gal-3 as a component of the catecholamine-induced myocardial stress response. At the same time, Gal-3 cannot be regarded as a mere innocent bystander, as our EV proteomic and *in vitro* data demonstrate that it actively modulates inflammatory NF-κB and redox-related signaling pathways. Conceptually, this aligns with the view that catecholamine-induced stress cardiomyopathy exists on a spectrum, in which transient regional contractile dysfunction and broader myocardial stress signatures such as Gal-3 induction, can dissociate (Zulfaj et al., 2024a, 2024b). Thus, other factors may determine the development of TS-like pattern of regional dysfunction, while the extracellular matrix signatures identified in EVs may represent early remodeling signals preceding structural fibrosis.

Our findings have implications for the role of Gal-3 in catecholamine-induced cardiac stress. In several cardiovascular models, pharmacological Gal-3 inhibition reduces inflammation, fibrosis and adverse remodeling (Boer et al., 2011; Henderson and Sethi 2009; Sharma et al., 2004; Calvier et al., 2015), which has prompted interest in Gal-3 as a therapeutic target. These studies have reported that elevated Gal-3 levels are associated with heart failure in human patients, and with increased risk of cardiovascular mortality. Our data suggest that in the setting of acute catecholamine-induced stress, Gal-3 inhibition may be more likely to modulate inflammatory and mitochondrial redox programs than to prevent the development of a TS-like contractile pattern per se. This distinction is clinically relevant since therapies that attenuate the stress cardiomyopathy axis might still improve long-term structural and functional outcomes, even if they do not fully abolish the acute wall motion abnormality. Finally, by establishing cardiac EVs as a rich source of Gal-3-associated stress signatures, our work highlights tissue-derived EV proteomics as a useful discovery strategy to map spatially resolved stress responses and to generate mechanistic hypothesis that can be tested *in vitro* and ultimately, in patients.

Our study has several important limitations. The TS model used has translational limitations, as catecholamine-centered induction via isoprenaline produces systemic effects that may not fully reflect the complex, multifactorial nature of human TS. Only healthy male rats were used, a common approach in catecholamine-based stress models to limit sex hormone–related variability and enhance reproducibility. This limits generalizability to both sexes and does not account for pre-existing comorbidities often present in patients with TS. As whole-region cardiac lysates were analyzed for EVs, the cellular origin of Gal-3-positive EVs was not determined. Future cell-specific isolation studies will be required to clarify how EVs from different cell types contribute to cardiac stress signaling. Although Gal-3 inhibition reduced inflammatory signaling *in vitro*, the lack of *in vivo* blockade limits causal inference and represents an important direction for future studies. While ultracentrifugation combined with iodixanol (OptiPrep) density gradients is widely regarded as yielding highly purified EVs, this approach is associated with low recovery and may still co-isolate small amounts of non-vesicular material. We were also unable to investigate Gal-3 levels in patients with TS, which would have provided important translational support for this framework. Future studies should determine whether Gal-3 enrichment in cardiac EVs is reflected in circulating EV populations, which could provide a clinically accessible biomarker of acute myocardial stress. However, based on our experimental findings, elevated plasma Gal-3 alone would not be expected to specify the presence or pattern of regional wall motion abnormalities. Rather, Gal-3 may better reflect systemic and myocardial stress exposure and the risk of subsequent remodeling than the immediate contractile phenotype.

In conclusion, our findings position Gal-3 as a stress-responsive mediator within inflammatory and mitochondrial redox programs activated during acute catecholamine exposure. Using region-specific EV proteomics as a spatial discovery platform, we identified Gal-3 as part of protein networks associated with immune activation and metabolic remodeling in the apical myocardium. Complementary functional studies demonstrated that Gal-3 can modulate NF-κB signaling and influence redox-related gene expression in cardiomyoblasts. Notably, myocardial Gal-3 induction occurred irrespective of whether animals developed a TS-like apical contractile phenotype, indicating that Gal-3 reflects catecholamine-driven myocardial stress rather than determining the spatial pattern of transient dysfunction. These findings support a hierarchical model in which Gal-3 contributes to the molecular stress axis engaged by catecholamine exposure, while additional region-specific mechanisms govern the emergence of TS-like wall motion abnormalities. By integrating spatial EV proteomics with mechanistic validation, this study identifies Gal-3 as a regulator of myocardial stress signaling rather than a phenotype-defining determinant in stress cardiomyopathy.

## Supplementary Information

Supplementary Material 1. Supplementary Appendix.

## Data Availability

The minimal dataset underlying the findings reported in this manuscript are, available from the corresponding author upon reasonable request.

## Abbreviations

BP: Biological processes
EVs: Extracellular vesicles
FAC: Fractional area change
FS: Fractional shortening
Gal-3: Galectin-3
GAPDH: Glyceraldehyde 3-phosphate dehydrogenase
GO: Gene ontology
GSEA: Gene set enrichment analysis
ISO: Isoprenaline
NF-κB: Nuclear factor-kappa B
NTA: Nanoparticle tracking analysis
LVAI: Left ventricular akinesia index
ORA: Over-representation analysis
PBS: Phosphate buffer saline
PPI: Protein–protein interaction
ROS: Reactive oxygen species
TNF-α: Tumor necrosis factor-α
TEM: Transmission electron microscopy
