# Supplementary material for: Galectin-3 associates with NF-κB activation and mitochondrial redox–related protein signatures in catecholamine-induced stress cardiomyopathy
Source: Mol Med. 2026 Apr 10;32:61. doi: 10.1186/s10020-026-01472-x (PMC13088642; doi:10.1186/s10020-026-01472-x)
Supplement: Supplementary file 1 — Supplementary Material 1. Supplementary Appendix. [file 10020_2026_1472_MOESM1_ESM.zip › Supplementary Appendix-Spearman correlation-Lgals3.docx]

| **GeneName** | **rho** | **p_value** | **Accession** |
| --- | --- | --- | --- |
| Lypla2 | -0,920588235 | 4,32041E-07 | Q9QYL8 |
| Kcnab2 | 0,879411765 | 7,18727E-06 | P62483 |
| Pdpn | 0,879411765 | 7,18727E-06 | Q64294 |
| Anxa1 | 0,864602275 | 2,44249E-15 | P07150 |
| St3gal4 | 0,850806452 | 6,94313E-10 | P61131 |
| Mpeg1 | 0,848784195 | 2,53131E-14 | Q9WV57 |
| Atp2a3 | 0,847058824 | 3,46805E-05 | P18596 |
| Fcer1g | 0,838235294 | 5,01034E-05 | P20411 |
| Ptprc | 0,838145897 | 1,0747E-13 | P04157 |
| Clic1 | 0,833360475 | 1,98952E-13 | Q6MG61 |
| Fn1 | 0,832329155 | 2,2693E-13 | P04937 |
| C1qa | 0,825119409 | 5,49782E-13 | P31720 |
| Cd74 | 0,823313783 | 7,2263E-09 | P10247 |
| Cd82 | 0,817759926 | 1,30429E-12 | O70352 |
| M6pr | 0,813222603 | 2,17915E-12 | Q6AY20 |
| C4 | 0,812440633 | 2,37765E-12 | P08649 |
| Tmed3 | 0,809477285 | 3,29492E-12 | Q6AY25 |
| Kidins220 | 0,808823529 | 0,000148476 | Q9EQG6 |
| Cd302 | 0,808823529 | 0,000148476 | Q5FVR3 |
| C9 | 0,80775076 | 3,9746E-12 | Q62930 |
| Fgb | 0,805547263 | 5,03575E-12 | P14480 |
| Cyba | 0,800953079 | 3,67896E-08 | Q62737 |
| Aldh3b1 | 0,8 | 0,000198571 | Q5XI42 |
| Ei24 | 0,797058824 | 0,000218098 | Q4KM77 |
| Gusb | 0,795821114 | 5,19402E-08 | P06760 |
| Cp | 0,795614297 | 1,41143E-11 | P13635 |
| Tm9sf4 | 0,794615719 | 1,56062E-11 | Q4KLL4 |
| Htra1 | 0,794072948 | 1,64784E-11 | Q9QZK5 |
| Sptlc2 | 0,788015307 | 2,99119E-11 | Q3B7D2 |
| Vkorc1l1 | 0,787819575 | 3,04838E-11 | Q6TEK3 |
| C4bpa | 0,787125488 | 3,25966E-11 | Q63514 |
| Clec10a | 0,786474164 | 3,47042E-11 | P49301 |
| Gabarap | 0,782352941 | 0,000341221 | P60517 |
| Hpx | 0,782283017 | 5,16722E-11 | P20059 |
| Rpl3 | 0,782023448 | 5,29465E-11 | P21531 |
| C4bpb | 0,780416316 | 6,15232E-11 | Q63515 |
| Enpp3 | 0,778006947 | 7,68727E-11 | P97675 |
| Gc | 0,776963579 | 8,45848E-11 | P04276 |
| Sptlc1 | 0,774825631 | 1,02725E-10 | D4A2H2 |
| Ctsz | 0,772057427 | 1,31701E-10 | Q9R1T3 |
| Fam3c | 0,771990775 | 1,32486E-10 | Q810F4 |
| Hsd17b11 | 0,76939778 | 1,66671E-10 | Q6AYS8 |
| S100a4 | 0,767961877 | 2,89002E-07 | P05942 |
| Arfrp1 | 0,767647059 | 0,000516884 | Q63055 |
| Coro1a | 0,76760659 | 1,94974E-10 | Q91ZN1 |
| Sfxn1 | 0,766420589 | 2,16145E-10 | Q63965 |
| Ugt1a6 | 0,765099444 | 3,40158E-07 | P08430 |
| Serpina10 | 0,763915651 | 2,68194E-10 | Q62975 |
| Pla2g4a | 0,762700825 | 2,97495E-10 | P50393 |
| Rap1b | 0,762687946 | 2,97821E-10 | Q62636 |
| F2 | 0,762612967 | 2,99727E-10 | P18292 |
| Cotl1 | 0,761730205 | 4,10902E-07 | B0BNA5 |
| Tmed5 | 0,761025922 | 3,4287E-10 | Q6AXN3 |
| Alox5ap | 0,759530792 | 4,64077E-07 | P20291 |
| Plg | 0,757673624 | 4,53976E-10 | Q01177 |
| F9 | 0,754349304 | 5,97036E-10 | P16296 |
| Lrrc59 | 0,752462887 | 6,96114E-10 | Q5RJR8 |
| Cfl1 | 0,751485874 | 7,53326E-10 | P45592 |
| Slc39a9 | 0,75 | 0,000819764 | Q3KR82 |
| Rpn2 | 0,749497911 | 8,83683E-10 | P25235 |
| Snx3 | 0,749335536 | 8,02638E-07 | Q5U211 |
| Lrp1 | 0,746960486 | 1,08104E-09 | G3V928 |
| Vamp8 | 0,746329417 | 1,1362E-09 | Q9WUF4 |
| Slc12a4 | 0,746275138 | 1,14106E-09 | Q63632 |
| Map1 | 0,746112302 | 1,15578E-09 | P01048 |
| Etfb | -0,744504154 | 1,31103E-09 | Q68FU3 |
| Tbxas1 | 0,744117647 | 0,000948283 | P49430 |
| Apoh | 0,743724048 | 1,39322E-09 | P26644 |
| C3 | 0,742332954 | 1,55195E-09 | P01026 |
| Coq9 | -0,742184108 | 1,56991E-09 | Q68FT1 |
| Serping1 | 0,739876239 | 1,8746E-09 | Q6P734 |
| Cd63 | 0,739856157 | 1,87748E-09 | P28648 |
| Hsd17b12 | 0,738661965 | 2,05645E-09 | Q6P7R8 |
| Il1rap | 0,735927917 | 2,52844E-09 | Q63621 |
| Tst | -0,735819357 | 2,54913E-09 | P24329 |
| Ogfod3 | 0,732352941 | 0,001254958 | Q5M843 |
| Slc17a5 | 0,732352941 | 0,001254958 | Q5Q0U0 |
| Tram1 | 0,732217006 | 3,33354E-09 | Q5XI41 |
| Apob | 0,732197134 | 3,33844E-09 | Q7TMA5 |
| Lyz1 | 0,729411765 | 0,001343079 | P00697 |
| Nsdhl | 0,729231688 | 4,15016E-09 | Q5PPL3 |
| Cybc1 | 0,728940512 | 4,23915E-09 | Q6AYA6 |
| Acsl4 | 0,726470588 | 0,001436183 | O35547 |
| Gpld1 | 0,726470588 | 0,001436183 | Q8R2H5 |
| Hp | 0,726463484 | 5,07219E-09 | P06866 |
| Mrrf | -0,726029256 | 5,2332E-09 | Q5RKI9 |
| Fgg | 0,725249674 | 5,5344E-09 | P02680 |
| Rab8a | 0,725017642 | 5,62714E-09 | P35280 |
| Mgat1 | 0,723607038 | 2,87156E-06 | Q09325 |
| Crp | 0,722935382 | 6,52731E-09 | P48199 |
| Lbr | 0,722900723 | 6,54338E-09 | O08984 |
| Apoe | 0,721341728 | 7,30582E-09 | P02650 |
| Tmem165 | 0,721041056 | 3,23587E-06 | Q4V899 |
| Sgpp1 | 0,720588235 | 0,001638191 | Q99P55 |
| Ptgis | 0,718104595 | 9,16315E-09 | Q62969 |
| Rab3d | 0,717647059 | 0,001747535 | Q63942 |
| Slc39a7 | 0,717635565 | 9,46643E-09 | Q6MGB4 |
| Rhoa | 0,717023129 | 9,87664E-09 | P61589 |
| Glul | 0,714705882 | 0,001862741 | P09606 |
| Far1 | 0,714433047 | 1,18031E-08 | Q66H50 |
| Etfa | -0,714359378 | 1,18627E-08 | P13803 |
| Cyp20a1 | 0,714033707 | 1,21298E-08 | Q6P7D4 |
| Isca1 | -0,713510287 | 1,25708E-08 | Q80W96 |
| Pdk2 | -0,71283958 | 1,31579E-08 | Q64536 |
| Cdc42 | 0,712568188 | 1,34027E-08 | Q8CFN2 |
| Slc39a6 | 0,711764706 | 0,001984043 | Q4V887 |
| H4c16; H4c2; Hist1h4m | 0,710125655 | 1,58066E-08 | P62804 |
| Tgfb2 | 0,709966349 | 1,59767E-08 | Q07257 |
| Serinc1 | 0,70931085 | 5,49694E-06 | Q7TNK0 |
| Ikbip | 0,708823529 | 0,002111681 | Q5EAJ6 |
| Cept1 | 0,708082289 | 1,81225E-08 | Q6AXM5 |
| Serpina3n | 0,707989579 | 1,82348E-08 | P09006 |
| Tufm | -0,706578376 | 2,00263E-08 | P85834 |
| Kdelr1 | 0,706054767 | 2,07321E-08 | Q569A6 |
| Glg1 | 0,70594621 | 2,08813E-08 | Q62638 |
| Sirpa | 0,70591109 | 2,09298E-08 | P97710 |
| Slc22a23 | 0,705882353 | 0,002245901 | Q9QZG1 |
| Arpc5 | 0,704976632 | 6,64302E-06 | Q4KLF8 |
| Cd44 | 0,703992184 | 2,37453E-08 | P26051 |
| Itgb1 | 0,703142812 | 2,51017E-08 | P49134 |
| Cdipt | 0,702453322 | 2,62558E-08 | P70500 |
| Atrn | 0,701115423 | 2,86384E-08 | Q99J86 |
| Fga | 0,701042119 | 2,87747E-08 | P06399 |
| Clu | 0,700246967 | 3,02919E-08 | P05371 |
| Retsat | 0,700230698 | 3,03238E-08 | Q8VHE9 |
| F10 | 0,698257614 | 3,44228E-08 | Q63207 |
| Dyrk1a | 0,697058824 | 0,002690589 | Q63470 |
| Slc6a6 | 0,695381232 | 9,986E-06 | P31643 |
| Pdp2 | -0,695014663 | 1,01396E-05 | O88484 |
| Tmem33 | 0,694443703 | 4,38575E-08 | Q9Z142 |
| Sec11a | 0,694222054 | 4,44742E-08 | P42667 |
| Hrg | 0,693209576 | 4,73959E-08 | Q99PS8 |
| Coq10b | -0,692695448 | 1,11619E-05 | Q5I0I9 |
| Tmco1 | 0,692107267 | 5,07807E-08 | Q5I0H4 |
| Tap1 | 0,691725242 | 5,20056E-08 | P36370 |
| Rac1 | 0,689627098 | 5,92411E-08 | Q6RUV5 |
| Cr1l | 0,68823274 | 6,45595E-08 | Q63135 |
| Sec61a1 | 0,687691684 | 6,6741E-08 | P61621 |
| Acads | -0,686387321 | 7,22869E-08 | P15651 |
| Rab7a | 0,686061659 | 7,37373E-08 | P09527 |
| C5ar1 | 0,685627442 | 7,57136E-08 | P97520 |
| Ifi30 | 0,68523042 | 7,75638E-08 | Q499T2 |
| Cct4 | 0,685193226 | 7,77393E-08 | Q7TPB1 |
| Gpr108 | 0,68414482 | 8,28412E-08 | Q6P6V6 |
| Tm9sf2 | 0,684000545 | 8,35673E-08 | Q66HG5 |
| Abhd6 | 0,683637746 | 8,54197E-08 | Q5XI64 |
| Polb | -0,683530385 | 1,61785E-05 | P06766 |
| Tor1aip1 | 0,682624981 | 9,07957E-08 | Q5PQX1 |
| Arpc1b | 0,682153713 | 9,34037E-08 | O88656 |
| Eci2 | -0,680853383 | 1,00968E-07 | Q5XIC0 |
| Gpat3 | 0,680815263 | 1,01198E-07 | Q4V8J4 |
| Uxs1 | 0,679411765 | 0,003793433 | Q5PQX0 |
| Akap13 | 0,679411765 | 0,003793433 | F1M3G7 |
| Tmem43 | 0,679187931 | 1,11493E-07 | Q5XIP9 |
| Ahsg | 0,678969794 | 1,12945E-07 | P24090 |
| Rpl9 | 0,678843807 | 1,13792E-07 | P17077 |
| Plpp1 | 0,67875268 | 1,14408E-07 | O08564 |
| Capza1 | 0,678571429 | 1,15643E-07 | B2GUZ5 |
| Pmpca | -0,677702996 | 1,21734E-07 | P20069 |
| Pa2g4 | 0,677612832 | 1,22383E-07 | Q6AYD3 |
| Mccc1 | -0,676726831 | 1,28938E-07 | Q5I0C3 |
| Tmem214 | 0,676564937 | 2,1266E-05 | A1L1L2 |
| Pttg1ip | 0,676470588 | 0,004008252 | Q6P767 |
| Nup210 | 0,675857577 | 1,35687E-07 | P11654 |
| Slc3a2 | 0,675785704 | 1,36259E-07 | Q794F9 |
| H2bc1 | 0,67558651 | 2,20859E-05 | Q00729 |
| Plvap | 0,674853372 | 2,27188E-05 | Q9WV78 |
| Fdxr | -0,674084729 | 1,50486E-07 | P56522 |
| Cpd | 0,673216273 | 1,58273E-07 | Q9JHW1 |
| Macroh2a1 | 0,672600955 | 1,64017E-07 | Q02874 |
| Sacm1l | 0,672076424 | 1,69066E-07 | Q9ES21 |
| Tpm3 | 0,671750753 | 1,72274E-07 | Q63610 |
| Gnai3 | 0,671298307 | 1,76824E-07 | P08753 |
| Canx | 0,670810153 | 1,8186E-07 | P35565 |
| Mgat2 | 0,670588235 | 0,004467209 | Q09326 |
| Penk | -0,670588235 | 0,004467209 | P04094 |
| C8b | 0,670429874 | 1,85875E-07 | P55314 |
| Actb | 0,670086306 | 1,89574E-07 | P60711 |
| Alcam | 0,669561442 | 1,95357E-07 | O35112 |
| Tmed7 | 0,668928271 | 2,02553E-07 | D3ZTX0 |
| Myh9 | 0,668729307 | 2,04865E-07 | Q62812 |
| Echs1 | -0,667788423 | 2,16135E-07 | P14604 |
| Glmp | 0,667521994 | 3,00083E-05 | Q68FV6 |
| Vcan | 0,666666847 | 0,004795904 | Q9ERB4 |
| Aldh6a1 | -0,666666668 | 2,30327E-07 | Q02253 |
| Gnb2 | 0,664676349 | 2,5767E-07 | P54313 |
| Cd93 | 0,664589443 | 3,34707E-05 | Q9ET61 |
| Zdhhc3 | 0,664589443 | 3,34707E-05 | Q2TGK3 |
| Afg1l | -0,664549749 | 2,59508E-07 | Q32PX9 |
| Mogs | 0,664549581 | 2,5951E-07 | O88941 |
| Idh3B | -0,663337585 | 2,77734E-07 | Q68FX0 |
| Akr7a2 | -0,663244741 | 3,51753E-05 | Q8CG45 |
| Serpina1 | 0,662722536 | 2,87432E-07 | P17475 |
| Cfi | 0,662396874 | 2,92695E-07 | Q9WUW3 |
| Hibadh | -0,66237855 | 2,92993E-07 | P29266 |
| Rab29 | 0,662251835 | 0,005188865 | Q63481 |
| Nrm | 0,662022209 | 3,67925E-05 | Q6MG14 |
| Yipf5 | 0,66113011 | 3,1403E-07 | Q5XID0 |
| Eef1a1 | 0,659465914 | 3,44267E-07 | P62630 |
| Eci1 | -0,658633231 | 3,60395E-07 | P23965 |
| Slc7a8 | 0,658271819 | 3,67612E-07 | Q9WVR6 |
| Capg | 0,657529786 | 3,82854E-07 | Q6AYC4 |
| Rab18 | 0,656950552 | 3,95159E-07 | Q5EB77 |
| Atp1b3 | 0,656860617 | 3,97102E-07 | Q63377 |
| Msn | 0,656769887 | 3,99072E-07 | O35763 |
| Slc25a25 | 0,655882353 | 0,005800737 | Q8K3P6 |
| Tmem120a | 0,655647832 | 4,24193E-07 | Q5HZE2 |
| Plek | 0,654752088 | 4,78689E-05 | Q4KM33 |
| Hadh | -0,654579999 | 4,49462E-07 | Q9WVK7 |
| Slc38a10 | 0,654363873 | 4,54744E-07 | E9PT23 |
| C1qbp | -0,654001683 | 4,63726E-07 | O35796 |
| Rpl28 | 0,653530913 | 4,75648E-07 | P17702 |
| Svip | 0,652941176 | 0,006102047 | P0C0A9 |
| Atl3 | 0,651088314 | 5,42223E-07 | Q0ZHH6 |
| Pcna | 0,651086063 | 5,45193E-05 | P04961 |
| Rpn1 | 0,650960812 | 5,45925E-07 | P07153 |
| Mrpl17 | -0,649560117 | 5,75237E-05 | Q6PDW6 |
| Tmem106b | 0,649496567 | 5,90172E-07 | Q6AYA5 |
| Ddx39b | 0,649193548 | 5,82672E-05 | Q63413 |
| Selenot | 0,648658955 | 6,16963E-07 | Q1H5H1 |
| Slc7a5 | 0,648336544 | 6,00392E-05 | Q63016 |
| Dad1 | 0,647524967 | 6,55039E-07 | P61805 |
| Mrpl22 | -0,647325427 | 6,61961E-07 | P0C2C0 |
| Sod1 | -0,646616553 | 6,87107E-07 | P07632 |
| Plpp3 | 0,646528796 | 6,90281E-07 | P97544 |
| Itih3 | 0,646222319 | 7,01473E-07 | Q63416 |
| Atg9a | 0,646063457 | 0,006855472 | Q5FWU3 |
| Mpst | -0,645660317 | 7,22438E-07 | P97532 |
| Tor1a | 0,644117647 | 0,007081524 | Q68G38 |
| Fh | -0,642748589 | 8,40709E-07 | P14408 |
| H2az1 | 0,642205818 | 8,64657E-07 | P0C0S7 |
| Ptbp1 | 0,640762463 | 7,79307E-05 | Q00438 |
| Mb | -0,640360399 | 9,50907E-07 | Q9QZ76 |
| Ormdl3 | 0,640303978 | 9,53666E-07 | Q6QI25 |
| Pdhb | -0,640212793 | 9,58141E-07 | P49432 |
| Wdr12 | 0,640029326 | 7,98936E-05 | P61480 |
| Il6st | 0,640029326 | 7,98936E-05 | P40190 |
| Itgb3 | 0,63851498 | 1,0451E-06 | Q8R2H2 |
| Hmgcl | -0,638478031 | 1,04708E-06 | P97519 |
| Ncbp1 | 0,638235294 | 0,007801139 | Q56A27 |
| Hspa1a | 0,637646548 | 1,09237E-06 | P0DMW0 |
| Sgpl1 | 0,6375726 | 1,09649E-06 | Q8CHN6 |
| Lamp1 | 0,636901535 | 1,1345E-06 | P14562 |
| Aco2 | -0,636306891 | 1,1692E-06 | Q9ER34 |
| Pfn1 | 0,636215601 | 1,17461E-06 | P62963 |
| Acadl | -0,636195857 | 1,17579E-06 | P15650 |
| Flnc | 0,635618521 | 1,2106E-06 | D3ZHA0 |
| Ak4 | -0,635092959 | 1,24312E-06 | Q9WUS0 |
| Ctsg | 0,635075722 | 1,2442E-06 | P17977 |
| Trim72 | -0,634912882 | 1,25445E-06 | A0JPQ4 |
| Hspd1 | -0,634658707 | 1,27061E-06 | P63039 |
| Tf | 0,633864358 | 1,32235E-06 | P12346 |
| Defa | 0,632352941 | 0,008577584 | Q62715 |
| Actr3 | 0,632110291 | 1,44361E-06 | Q4V7C7 |
| Cant1 | 0,631656129 | 0,000105668 | Q8K4Y7 |
| Hsd17b10 | -0,63026191 | 1,58251E-06 | O70351 |
| Gtpbp4 | 0,630010585 | 1,60233E-06 | Q99P77 |
| Yif1b | 0,629411765 | 0,008988089 | Q6PEC3 |
| Afm | 0,629179331 | 1,66952E-06 | P36953 |
| Orm1 | 0,628962223 | 1,6875E-06 | P02764 |
| Arpc2 | 0,628853669 | 1,69656E-06 | P85970 |
| Ogdh | -0,628636561 | 1,7148E-06 | Q5XI78 |
| Litaf | 0,628403408 | 0,009132348 | P0C0T0 |
| Gfm1 | -0,627927808 | 1,77565E-06 | Q07803 |
| Tmed2 | 0,627693645 | 1,79619E-06 | Q63524 |
| Magt1 | 0,626950742 | 1,86282E-06 | O35777 |
| Pon1 | 0,626696345 | 1,88615E-06 | P55159 |
| Pip4p1 | 0,626470588 | 0,009413977 | Q5PPM8 |
| Ccdc175 | 0,626470588 | 0,009413977 | Q5PQJ9 |
| Sdc2 | 0,626466276 | 0,000125156 | P34900 |
| Glrx | 0,626065245 | 1,94523E-06 | Q9ESH6 |
| C5 | 0,625739566 | 1,97638E-06 | P08650 |
| Gpnmb | 0,625057284 | 0,000130973 | Q6P7C7 |
| Fetub | 0,624528456 | 2,09634E-06 | Q9QX79 |
| C1qc | 0,624094228 | 2,14097E-06 | P31722 |
| Elovl5 | 0,623529412 | 0,00985565 | Q920L7 |
| Coq8a | -0,623117215 | 2,24463E-06 | Q5BJQ0 |
| Abhd18 | -0,622434018 | 0,000142449 | Q4V7A8 |
| Niban2 | 0,622282537 | 2,33686E-06 | B4F7E8 |
| Tcp1 | 0,622119693 | 2,35525E-06 | P28480 |
| Alb | 0,620946075 | 2,49187E-06 | P02770 |
| Ncstn | 0,620637248 | 2,52903E-06 | Q8CGU6 |
| Hmbs | -0,620588235 | 0,010313516 | P19356 |
| Man2a1 | 0,619914783 | 2,61796E-06 | P28494 |
| Mrps26 | -0,619063129 | 2,72651E-06 | Q9EPJ3 |
| Gsn | 0,618411769 | 2,81233E-06 | Q68FP1 |
| Hspb1 | 0,618394985 | 2,81458E-06 | P42930 |
| Fhl1 | 0,617814689 | 2,8932E-06 | Q9WUH4 |
| Jagn1 | 0,617647059 | 0,010787985 | Q4KM64 |
| Serpinb1a | 0,617647059 | 0,010787985 | Q4G075 |
| H3-3b | 0,616912723 | 3,01945E-06 | P84245 |
| Gnb4 | 0,616568915 | 0,000171414 | O35353 |
| Tmbim6 | 0,616495237 | 3,0796E-06 | P55062 |
| Suclg1 | -0,615898174 | 3,16755E-06 | P13086 |
| Pdha1 | -0,615714482 | 3,19508E-06 | P26284 |
| Fcn2 | 0,61459549 | 3,36759E-06 | P57756 |
| Acaa2 | -0,614161262 | 3,43683E-06 | P13437 |
| Srprb | 0,613689411 | 3,51356E-06 | Q4FZX7 |
| Acbd3 | 0,613689411 | 3,51356E-06 | Q7TNY6 |
| Pnpo | -0,613382228 | 0,000189258 | O88794 |
| Rpl35a | 0,612967135 | 3,6341E-06 | P04646 |
| Ech1 | -0,612490845 | 3,71567E-06 | Q62651 |
| Acat1 | -0,612440971 | 3,7243E-06 | P17764 |
| Aldh5a1 | -0,612261514 | 3,75554E-06 | P51650 |
| Nolc1 | 0,612098679 | 3,7841E-06 | P41777 |
| Sec11c | 0,611952452 | 3,80991E-06 | Q9WTR7 |
| Efhd2 | 0,611859594 | 0,000198354 | Q4FZY0 |
| Daglb | 0,611764706 | 0,011788405 | P0C1S9 |
| Plscr1 | 0,611764706 | 0,011788405 | P58195 |
| Rps15a | 0,611405157 | 3,90798E-06 | P62246 |
| Gpr107 | 0,610753744 | 4,02774E-06 | D3ZWZ9 |
| Grpel1 | -0,610574317 | 4,06133E-06 | P97576 |
| Crat | -0,610340618 | 4,10545E-06 | Q704S8 |
| Prdx3 | -0,610307488 | 4,11174E-06 | Q9Z0V6 |
| Ndufaf3 | -0,610085771 | 4,15408E-06 | O08776 |
| Mipep | -0,610048047 | 4,16132E-06 | Q01992 |
| Hspe1 | -0,609856709 | 4,19824E-06 | P26772 |
| Rpl8 | 0,60949331 | 4,26919E-06 | P62919 |
| Atp2b1 | 0,608912773 | 4,38485E-06 | P11505 |
| A1m | 0,608896247 | 4,38818E-06 | Q63041 |
| Gstk1 | -0,608207133 | 4,52933E-06 | P24473 |
| Atp6v0a1 | 0,608082069 | 4,55539E-06 | P25286 |
| Apoa1 | 0,607501494 | 4,67821E-06 | P04639 |
| Hsph1 | 0,607251411 | 4,73205E-06 | Q66HA8 |
| Hnrnpa1 | 0,606198774 | 4,96502E-06 | P04256 |
| Slc11a1 | 0,605882353 | 0,012860299 | P70553 |
| Anpep | 0,605639537 | 5,09306E-06 | P15684 |
| Qdpr | -0,605634571 | 5,09421E-06 | P11348 |
| Ccn2 | 0,604988195 | 5,24604E-06 | Q9R1E9 |
| Tmx2 | 0,604950335 | 5,25507E-06 | Q5XIK2 |
| Tmed9 | 0,603989689 | 5,48884E-06 | Q5I0E7 |
| Gnai2 | 0,603664 | 5,57025E-06 | P04897 |
| Bst1 | 0,603017803 | 5,7351E-06 | Q63072 |
| Dpy19l1 | 0,602958341 | 5,7505E-06 | D4AD75 |
| Mtfmt | -0,602941176 | 0,013424125 | Q5I0C5 |
| Coq5 | -0,602453456 | 5,88275E-06 | Q4G064 |
| Ddost | 0,601693536 | 6,08712E-06 | Q641Y0 |
| Rps3 | 0,60151437 | 6,13625E-06 | P62909 |
| Adam17 | 0,601173021 | 0,000273929 | Q9Z1K9 |
| Basp1 | 0,601172384 | 6,23105E-06 | Q05175 |
| Ppm1k | -0,600911957 | 6,30415E-06 | A6K136 |
| Anxa2 | 0,600879336 | 6,31336E-06 | Q07936 |
| Hmox1 | 0,600441664 | 0,013918336 | P06762 |
| Piezo1 | 0,6 | 0,014007122 | Q0KL00 |
| Chmp5 | 0,6 | 0,014007122 | Q4QQV8 |
| RT1-Bb | 0,599869735 | 6,60469E-06 | P29826 |
| Ptgs1 | 0,599869735 | 6,60469E-06 | Q63921 |
| Spns1 | 0,599652627 | 6,66894E-06 | Q2YDU8 |
| Stat3 | 0,599001303 | 6,86517E-06 | P52631 |
| Ldlr | 0,598366217 | 7,06164E-06 | P35952 |
| Myo1d | 0,59825766 | 7,09573E-06 | Q63357 |
| Idh3g | -0,597855885 | 7,22326E-06 | P41565 |
| Ak3 | -0,597714875 | 7,26852E-06 | P29411 |
| Gdap2 | 0,597058824 | 0,014609732 | Q66H63 |
| Rap2b | 0,595972211 | 7,84988E-06 | P61227 |
| Idh2 | -0,595250375 | 8,10304E-06 | P56574 |
| Rpl18a | 0,595109507 | 8,15331E-06 | P62718 |
| Clybl | -0,594946672 | 8,21178E-06 | Q5I0K3 |
| Auh | -0,594924686 | 8,21971E-06 | F1LU71 |
| Lpl | -0,594729558 | 8,29035E-06 | Q06000 |
| Sod2 | -0,594474299 | 8,38361E-06 | P07895 |
| Yars2 | -0,594273309 | 8,45772E-06 | Q5I0L3 |
| Apoc1 | 0,594023937 | 8,55052E-06 | P19939 |
| Pcmt1 | -0,592823801 | 9,01039E-06 | P22062 |
| Inpp5j | -0,592612962 | 0,00035189 | Q9JMC1 |
| C1qb | 0,592053843 | 9,31734E-06 | P31721 |
| Atp5f1b | -0,592009557 | 9,33528E-06 | P10719 |
| Rpl11 | 0,591907075 | 9,37693E-06 | P62914 |
| Nr3c1 | 0,591176471 | 0,01587558 | P06536 |
| Dlat | -0,590767227 | 9,85187E-06 | P08461 |
| Tars2 | -0,589448545 | 1,0429E-05 | Q68FW7 |
| P4ha1 | 0,588698299 | 1,07711E-05 | P54001 |
| Agpat4 | 0,588580113 | 1,08259E-05 | Q924S1 |
| Slc38a2 | 0,588465194 | 1,08795E-05 | Q9JHE5 |
| Brd2 | 0,588235294 | 0,016539722 | Q6MGA9 |
| Tmem192 | 0,588235294 | 0,016539722 | Q5U1Y0 |
| Sugt1 | 0,588235294 | 0,016539722 | B0BN85 |
| Hsdl2 | -0,586875103 | 1,16461E-05 | Q4V8F9 |
| Adam10 | 0,586859175 | 1,1654E-05 | Q10743 |
| Mgst1 | 0,586782469 | 1,16922E-05 | P08011 |
| Wdr5 | 0,586510264 | 0,000418899 | Q498M4 |
| Pebp1 | -0,586191924 | 1,19905E-05 | P31044 |
| Macf1 | 0,585410557 | 0,000432107 | D3ZHV2 |
| Mfge8 | 0,585393655 | 1,24048E-05 | P70490 |
| A2m | 0,585294118 | 0,017225287 | P06238 |
| Agtrap | 0,585294118 | 0,017225287 | Q642A2 |
| Rpl10 | 0,584455059 | 1,29088E-05 | Q6PDV7 |
| Slc38a1 | 0,584145249 | 1,30793E-05 | Q9JM15 |
| Agps | 0,583362738 | 1,35191E-05 | Q9EQR2 |
| Magmas-ps1 | -0,583211144 | 0,000459635 | Q6EIX2 |
| Hnrnpd | 0,582352941 | 0,017932734 | Q9JJ54 |
| Mrpl38 | -0,582261305 | 1,41613E-05 | Q5PQN9 |
| Mrpl16 | -0,582082666 | 1,42681E-05 | Q5M818 |
| Bckdha | -0,581967217 | 1,43375E-05 | P11960 |
| Pgam5 | 0,581919831 | 1,43661E-05 | Q562B5 |
| Rpl37a | 0,581756995 | 1,44647E-05 | P61515 |
| Xirp2 | 0,580524345 | 1,52315E-05 | Q71LX6 |
| Ndufaf7 | -0,580278592 | 0,000498745 | Q5XI79 |
| Pgd | 0,579493026 | 1,59016E-05 | P85968 |
| Osmr | 0,579411765 | 0,018662529 | Q65Z14 |
| Mybbp1a | 0,579067376 | 1,61861E-05 | O35821 |
| Myh10 | 0,578950226 | 1,62652E-05 | Q9JLT0 |
| Qrsl1 | -0,578315464 | 0,000526544 | Q5FWT5 |
| Ctsl | 0,577346041 | 0,000540769 | P07154 |
| Slc7a1 | 0,576979472 | 0,000546236 | P30823 |
| Pspc1 | 0,576470588 | 0,01941514 | Q4KLH4 |
| Ldhb | -0,576437702 | 1,80497E-05 | P42123 |
| Gnb1 | 0,575964827 | 1,8405E-05 | P54311 |
| Alox5 | 0,575879288 | 1,847E-05 | P12527 |
| Mlec | 0,574375119 | 1,96475E-05 | Q5FVQ4 |
| Bpnt2 | 0,573630788 | 2,02553E-05 | D4AD37 |
| Msi1 | 0,573529412 | 0,020191038 | Q8K3P4 |
| Clcc1 | 0,573343827 | 2,04943E-05 | Q9WU61 |
| Colec12 | 0,573180992 | 2,0631E-05 | Q4V885 |
| Rps2 | 0,573049262 | 2,07422E-05 | P27952 |
| Yipf3 | 0,572580645 | 0,000615748 | Q6TUD4 |
| Rmdn1 | -0,572211823 | 2,14623E-05 | Q4G069 |
| P2rx4 | 0,571847507 | 0,000628062 | P51577 |
| Rps20 | 0,571839549 | 2,17898E-05 | P60868 |
| Lonp1 | -0,571552637 | 2,20453E-05 | Q924S5 |
| RT1-Db1 | 0,571296749 | 2,22754E-05 | P18211 |
| Naxe | -0,571281244 | 2,22895E-05 | B0BNM1 |
| Arl5a | 0,571040849 | 2,25078E-05 | P51646 |
| Agt | 0,570629902 | 2,28857E-05 | P01015 |
| Aldh9a1 | -0,57059111 | 2,29217E-05 | Q9JLJ3 |
| Pkn1 | 0,570588235 | 0,020990696 | Q63433 |
| Ptpn6 | 0,570588235 | 0,020990696 | P81718 |
| Rpl27a | 0,56959071 | 2,38675E-05 | P18445 |
| Parp1 | 0,568970205 | 2,4472E-05 | P27008 |
| Ykt6 | 0,56889299 | 2,45482E-05 | Q5EGY4 |
| Gabarapl2 | 0,568548387 | 0,000686205 | P60522 |
| Rab35 | 0,568513041 | 2,49263E-05 | Q5U316 |
| Rab11a | 0,568181818 | 0,000692949 | P62494 |
| Npc1l1 | 0,567647059 | 0,021814592 | Q6T3U3 |
| Acot2 | -0,567536028 | 2,59234E-05 | O55171 |
| Tgfbr2 | 0,567082111 | 0,000713534 | P38438 |
| Hagh | -0,566884686 | 2,66084E-05 | O35952 |
| Clcn3 | 0,566652193 | 2,68569E-05 | P51792 |
| Tkt | 0,566504736 | 2,70156E-05 | P50137 |
| Slc15a4 | 0,566348974 | 0,000727554 | O09014 |
| Nfs1 | -0,565799115 | 2,77871E-05 | Q99P39 |
| Rab27a | 0,565615836 | 0,000741816 | P23640 |
| Pcca | -0,565558422 | 2,80548E-05 | P14882 |
| Tnni1 | -0,564705882 | 0,022663205 | P13413 |
| Decr1 | -0,564442153 | 2,93278E-05 | Q64591 |
| Smc3 | 0,564162418 | 2,9655E-05 | P97690 |
| Banf1 | 0,56414956 | 0,000771081 | Q9R1T1 |
| Cs | -0,563829787 | 3,00485E-05 | Q8VHF5 |
| Hibch | -0,563712853 | 3,01879E-05 | Q5XIE6 |
| Fabp3 | -0,563395571 | 3,05693E-05 | P07483 |
| Pfkp | 0,563049853 | 0,000793692 | P47860 |
| Nudt2 | -0,563049853 | 0,000793692 | Q6PEC0 |
| Timm44 | -0,562868076 | 3,12132E-05 | O35094 |
| Glt8d1 | 0,562178224 | 0,023412621 | Q6AYF6 |
| Pdk1 | -0,562069154 | 3,22121E-05 | Q63065 |
| Prdx5 | -0,561891063 | 3,24387E-05 | Q9R063 |
| Prg2 | 0,561764706 | 0,023537016 | Q63189 |
| Cpa3 | 0,561734934 | 3,26386E-05 | P21961 |
| Prnp | 0,561076886 | 3,34936E-05 | P13852 |
| Eef2 | 0,560983554 | 3,36165E-05 | P05197 |
| Slc25a1 | 0,560859772 | 3,37802E-05 | P32089 |
| Lyn | 0,560642658 | 3,4069E-05 | Q07014 |
| Slc41a3 | -0,560117302 | 0,000856865 | Q3SWT5 |
| Ndrg1 | 0,559704733 | 3,53429E-05 | Q6JE36 |
| Cnnm4 | 0,559234883 | 0,024309145 | P0C588 |
| Cpt2 | -0,5588363 | 3,65612E-05 | P18886 |
| Cyp51a1 | 0,558823529 | 0,024436511 | Q64654 |
| Brix1 | 0,558823529 | 0,024436511 | Q4QQT6 |
| Comt | 0,558664678 | 3,68065E-05 | P22734 |
| Mtdh | 0,55829353 | 3,73421E-05 | Q9Z1W6 |
| Emc3 | 0,558152326 | 0,000901628 | Q5U2V8 |
| Actr2 | 0,558091568 | 3,76366E-05 | Q5M7U6 |
| Kyat3 | -0,558013301 | 3,77513E-05 | Q58FK9 |
| Ssr4 | 0,557425098 | 3,86235E-05 | Q07984 |
| Rpl14 | 0,557060276 | 3,91737E-05 | Q63507 |
| Klhl41 | 0,556448111 | 4,01131E-05 | Q9ER30 |
| Acadm | -0,55625221 | 4,04181E-05 | P08503 |
| Slc20a2 | 0,556083263 | 4,06828E-05 | Q63488 |
| Rer1 | 0,555882353 | 0,025362176 | Q498C8 |
| Bckdk | -0,55510625 | 4,2245E-05 | Q00972 |
| Rpl15 | 0,5548198 | 4,27133E-05 | P61314 |
| Coq6 | -0,554756414 | 4,28176E-05 | Q68FU7 |
| Icam1 | 0,554618768 | 0,000987314 | Q00238 |
| Got2 | -0,554307117 | 4,35635E-05 | P00507 |
| Ak2 | -0,554035717 | 4,40198E-05 | P29410 |
| Mdh2 | -0,553640731 | 4,46917E-05 | P04636 |
| Capzb | 0,553625706 | 4,47174E-05 | Q5XI32 |
| Aldh4a1 | -0,553330077 | 4,52267E-05 | P0C2X9 |
| C1galt1c1 | 0,552941176 | 0,0263145 | Q499P3 |
| Rmdn3 | 0,552941176 | 0,0263145 | Q66H15 |
| Cdc37 | 0,551958097 | 4,76606E-05 | Q63692 |
| Acadvl | -0,551879497 | 4,78036E-05 | P45953 |
| Rps4x | 0,551701678 | 4,81285E-05 | P62703 |
| Kcnj5 | 0,551128962 | 4,9189E-05 | P48548 |
| H1-1 | 0,550601135 | 5,01853E-05 | D4A3K5 |
| Lpcat3 | 0,550601135 | 5,01853E-05 | Q5FVN0 |
| Ddx39a | 0,550453239 | 5,04677E-05 | Q5U216 |
| Reep4 | 0,55 | 0,027293973 | Q4QQW1 |
| Ninj1 | 0,549732679 | 5,18648E-05 | P70617 |
| Asl | 0,549720468 | 0,001117886 | P20673 |
| Vat1 | 0,548647109 | 5,40365E-05 | Q3MIE4 |
| Rps18 | 0,548429995 | 5,44807E-05 | P62271 |
| Cavin1 | -0,548089449 | 5,51842E-05 | P85125 |
| Tm9sf1 | 0,547987921 | 0,001167565 | Q66HF2 |
| Mug1 | 0,547670095 | 5,60619E-05 | Q03626 |
| Cntnap1 | 0,547653959 | 0,00117736 | P97846 |
| Vwf | 0,547349569 | 5,67413E-05 | Q62935 |
| Slc30a1 | 0,547058824 | 0,028301087 | Q62720 |
| Pycr2 | 0,547058824 | 0,028301087 | Q6AY23 |
| Aox3 | 0,547058824 | 0,028301087 | Q5QE80 |
| A1i3 | 0,54681648 | 5,78881E-05 | P14046 |
| Rab1A | 0,54659936 | 5,83612E-05 | Q6NYB7 |
| Rps27a | 0,546554252 | 0,001210126 | P62982 |
| Lmbrd1 | 0,545989846 | 0,028674071 | Q6AZ61 |
| Hspb7 | 0,545933183 | 5,9835E-05 | Q9QUK5 |
| Cd47 | 0,545489092 | 6,08363E-05 | P97829 |
| Osgepl1 | -0,545158489 | 6,15916E-05 | Q4V7F3 |
| Mlycd | -0,545133801 | 6,16484E-05 | Q920F5 |
| Gyg1 | -0,545064727 | 6,18074E-05 | O08730 |
| Nptn | 0,544901892 | 6,21838E-05 | P97546 |
| Rpl31 | 0,544724273 | 6,25967E-05 | P62902 |
| Gna13 | 0,543342562 | 6,58958E-05 | Q6Q7Y5 |
| Pdgfrb | 0,542878854 | 6,70381E-05 | Q05030 |
| H1-5 | 0,542543088 | 6,78765E-05 | D3ZBN0 |
| Emc8 | 0,542528362 | 6,79135E-05 | Q5FVL2 |
| Epdr1 | -0,542296524 | 6,84983E-05 | Q5XII0 |
| Cap1 | 0,542039842 | 6,91511E-05 | Q08163 |
| Idh3a | -0,541551323 | 7,04092E-05 | Q99NA5 |
| Lifr | -0,541176471 | 0,030400219 | O70535 |
| Msmo1 | 0,54068915 | 0,001398719 | O35532 |
| Lrrc8c | 0,540322581 | 0,001411315 | Q498T9 |
| App | 0,539947894 | 7,46873E-05 | P08592 |
| Hk2 | 0,539760083 | 7,52037E-05 | P27881 |
| Myadm | 0,539419763 | 7,61476E-05 | Q6VBQ5 |
| Pdp1 | -0,538753799 | 7,80262E-05 | O88483 |
| Adgrg1 | 0,538631492 | 0,031344276 | Q8K3V3 |
| Saraf | 0,53835579 | 0,001480607 | Q6AYN2 |
| Tmem30a | 0,538038506 | 0,00149206 | Q6AY41 |
| Sec22b | 0,537806004 | 8,07728E-05 | Q4KM74 |
| Ndufaf6 | -0,53776632 | 8,08897E-05 | D3ZN43 |
| B4galt4 | 0,537390029 | 0,001515707 | Q66HH1 |
| Copb1 | 0,537263204 | 8,23853E-05 | P23514 |
| Rpl13a | 0,537223506 | 8,25044E-05 | P35427 |
| Rlc-a | 0,537154644 | 8,27113E-05 | P13832 |
| Taco1 | -0,536789254 | 8,38172E-05 | B2RYT9 |
| Tspan2 | 0,536522777 | 0,001547844 | Q9JJW1 |
| Pmpcb | -0,53624644 | 8,54851E-05 | Q03346 |
| Vti1b | 0,536177605 | 8,56988E-05 | P58200 |
| RT1-Ba | 0,535689085 | 8,72292E-05 | P20037 |
| Gba2 | 0,535294118 | 0,032615863 | Q5M868 |
| Kbtbd8 | 0,535294118 | 0,032615863 | B1H285 |
| Ostf1 | 0,534457478 | 0,001626781 | Q6P686 |
| Copb2 | 0,533557685 | 9,42026E-05 | O35142 |
| Ndufaf4 | -0,53343465 | 9,46202E-05 | Q9NQR8 |
| Lyrm7 | -0,532797786 | 9,68093E-05 | B4F7A1 |
| Csf1r | 0,532624633 | 0,001699752 | Q00495 |
| Masp1 | -0,532352941 | 0,033768623 | Q8CHN8 |
| Bet1 | 0,531439056 | 0,00174846 | Q62896 |
| Clta | 0,530720799 | 0,000104273 | P08081 |
| Rpl32 | 0,530695327 | 0,000104368 | P62912 |
| Wls | 0,530329762 | 0,000105736 | Q6P689 |
| Rab2a | 0,529989688 | 0,000107023 | P05712 |
| Hgs | 0,529411765 | 0,034952007 | Q9JJ50 |
| Dhrs7b | 0,529164297 | 0,000110207 | Q5RJY4 |
| S100a10 | 0,528849808 | 0,000111443 | P05943 |
| Cmbl | -0,528715669 | 0,000111974 | Q7TP52 |
| Rpl29 | 0,528441164 | 0,000113067 | P25886 |
| Zdhhc17 | 0,528401227 | 0,000113227 | E9PTT0 |
| Tpt1 | 0,528198448 | 0,000114042 | P63029 |
| Park7 | -0,527804163 | 0,000115643 | O88767 |
| Apoc4 | 0,527532771 | 0,000116756 | P55797 |
| Tmem38b | 0,527412879 | 0,000117251 | Q68FV1 |
| Preb | 0,526812853 | 0,000119757 | Q9WTV0 |
| Cfd | 0,526595745 | 0,000120675 | P32038 |
| Dars2 | -0,526470588 | 0,036166515 | Q3KRD0 |
| Dgat1 | 0,526470588 | 0,036166515 | Q9ERM3 |
| Gatc | -0,526470588 | 0,036166515 | D3ZY68 |
| Hdlbp | 0,526470588 | 0,036166515 | Q9Z1A6 |
| Mrpl37 | -0,526012973 | 0,000123173 | Q6AXT0 |
| Hnrnpa2b1 | 0,525850137 | 0,000123879 | A7VJC2 |
| Capn5 | 0,525850137 | 0,000123879 | Q8R4C0 |
| Slc33a1 | 0,525659824 | 0,002003721 | Q6AYY8 |
| Rps10 | 0,525618758 | 0,000124889 | P63326 |
| Eml1 | -0,525510204 | 0,000125366 | Q4V8C3 |
| Hnrnpm | 0,525198795 | 0,000126742 | Q62826 |
| Prdx6 | -0,52465601 | 0,000129173 | O35244 |
| Rnh1 | 0,52286482 | 0,0001375 | P29315 |
| Ndufa11 | 0,521847691 | 0,000142443 | Q80W89 |
| Pex14 | 0,521710812 | 0,00014312 | Q642G4 |
| Tcn2 | 0,521125472 | 0,002226124 | Q9R0D6 |
| Fxn | -0,520971443 | 0,038522524 | D3ZYW7 |
| Sort1 | 0,520544972 | 0,00014901 | O54861 |
| Lta4h | 0,520408163 | 0,000149715 | P30349 |
| Ran | 0,520287693 | 0,000150339 | P62828 |
| Tpi1 | -0,520247533 | 0,000150547 | P48500 |
| Timm8b | -0,519839332 | 0,000152681 | P62078 |
| Klhl21 | 0,519499773 | 0,039172207 | D4A2K4 |
| Pars2 | -0,519187972 | 0,000156142 | Q5M7W7 |
| Ddx21 | 0,51903922 | 0,000156943 | Q3B8Q1 |
| Mcam | 0,518742556 | 0,002351376 | Q9EPF2 |
| Cavin3 | -0,518562744 | 0,000159532 | Q9Z1H9 |
| Rps6ka1 | 0,51845419 | 0,000160127 | Q63531 |
| Esyt1 | 0,518007873 | 0,000162596 | Q9Z1X1 |
| Ubxn4 | 0,517993813 | 0,000162675 | Q5HZY0 |
| Cd151 | 0,517925476 | 0,000163056 | Q9QZA6 |
| Snd1 | 0,51791142 | 0,000163134 | Q66X93 |
| Ca4 | -0,517111298 | 0,000167661 | P48284 |
| Lrpprc | -0,516622792 | 0,00017048 | Q5SGE0 |
| Aldh7a1 | -0,516528253 | 0,000171031 | Q64057 |
| Hspa9 | -0,516473974 | 0,000171347 | P48721 |
| Rnps1 | 0,516405678 | 0,000171747 | Q6AYK1 |
| Kras | 0,516176314 | 0,000173094 | P08644 |
| Magoh | 0,51597145 | 0,000174306 | Q27W02 |
| Oxct1 | -0,515631785 | 0,000176332 | B2GV06 |
| Ntn1 | -0,515631785 | 0,000176332 | Q924Z9 |
| Rpl7a | 0,5155915 | 0,000176574 | P62425 |
| Ufd1 | -0,515259832 | 0,002545429 | Q9ES53 |
| Serpind1 | 0,514709928 | 0,002577305 | Q64268 |
| Map2k2 | 0,514705882 | 0,041345806 | P36506 |
| Cavin2 | -0,514329136 | 0,000184302 | Q66H98 |
| Lbp | 0,514329136 | 0,000184302 | Q63313 |
| Rpl23 | 0,51380031 | 0,00018763 | P62832 |
| Rpl24 | 0,513759975 | 0,000187886 | P83732 |
| Anxa4 | 0,513460703 | 0,000189796 | P55260 |
| Rbm8a | 0,513135041 | 0,000191895 | Q27W01 |
| Gnpat | 0,512782935 | 0,000194187 | Q9ES71 |
| Cltc | 0,511900562 | 0,000200041 | P11442 |
| Ssr1 | 0,511887963 | 0,000200126 | Q7TPJ0 |
| Ralb | 0,511684721 | 0,000201497 | P36860 |
| Lamp2 | 0,511317376 | 0,000203998 | P17046 |
| Stim1 | 0,51016365 | 0,000212035 | P84903 |
| Col3a1 | 0,510127397 | 0,002856671 | P13941 |
| Spryd4 | -0,509878419 | 0,000214066 | Q4FZT8 |
| Rpl12 | 0,509797536 | 0,000214645 | P23358 |
| Gpt | -0,509661311 | 0,000215624 | P25409 |
| Gcc2 | 0,509577493 | 0,002891894 | D3ZZL9 |
| Coq4 | -0,509458029 | 0,000217091 | Q4FZU1 |
| Rpl10a | 0,509227095 | 0,00021877 | P62907 |
| Nae1 | -0,509198078 | 0,043953521 | Q9Z1A5 |
| Mccc2 | -0,509037617 | 0,000220156 | Q5XIT9 |
| Gcsh | -0,508983337 | 0,000220554 | Q5I0P2 |
| Sh3bgrl3 | 0,508823529 | 0,044135218 | B2RZ27 |
| Syt1 | -0,508823529 | 0,044135218 | P21707 |
| Jup | -0,508141554 | 0,000226819 | Q6P0K8 |
| Golga7 | 0,507871027 | 0,000228866 | Q6AYQ1 |
| Rps19 | 0,507775396 | 0,000229593 | P17074 |
| Pcyox1 | 0,507734897 | 0,000229902 | Q99ML5 |
| Arf4 | 0,5072334 | 0,000233757 | P61751 |
| Pgrmc1 | 0,506827974 | 0,003073706 | P70580 |
| Gpx3 | 0,506324481 | 0,003108049 | P23764 |
| Plod2 | 0,506231672 | 0,003114415 | Q811A3 |
| Psen1 | 0,504994961 | 0,003200333 | P97887 |
| RT1-Aw2 | 0,504857937 | 0,003209977 | P15978 |
| Anxa3 | -0,504260979 | 0,000257835 | P14669 |
| Smc1a | 0,503541673 | 0,00026399 | Q9Z1M9 |
| Cbr4 | -0,503365176 | 0,00026552 | Q7TS56 |
| Man2c1 | 0,502941176 | 0,04706314 | P21139 |
| Slco3a1 | 0,502932551 | 0,003348173 | Q99N02 |
| Ctcf | 0,502727495 | 0,000271117 | Q9R1D1 |
| Pecr | -0,502293267 | 0,000274989 | Q9WVK3 |
| Nop58 | 0,501924848 | 0,003422527 | Q9QZ86 |
| Rab8b | 0,501832845 | 0,003429386 | P70550 |
| Ece1 | 0,501777719 | 0,00027965 | P42893 |
| Pex3 | 0,50127558 | 0,00028426 | Q9JJK4 |
| Hbb | -0,50109914 | 0,000285896 | P02091 |
| Cct5 | 0,500882026 | 0,00028792 | Q68FQ0 |
| Pon3 | 0,5 | 0,048580289 | Q68FP2 |
| Ncln | 0,499660743 | 0,000299553 | Q5XIA1 |
| Selenbp1 | -0,499321501 | 0,000302859 | Q8VIF7 |
| Mrpl2 | -0,499267221 | 0,000303391 | Q498T4 |
| Rplp0 | 0,499104381 | 0,000304992 | P19945 |
| Pc | -0,498439493 | 0,000311609 | P52873 |
| Top1 | 0,498222379 | 0,000313798 | Q9WUL0 |
| Tmed10 | 0,497910222 | 0,000316969 | Q63584 |
| Septin4 | -0,497800587 | 0,003741892 | A0A096MJN4 |
| Dlst | -0,497557271 | 0,00032059 | Q01205 |
| Sec22a | 0,497058824 | 0,050133558 | Q642F4 |
| Vamp7 | 0,496746405 | 0,003827529 | Q9JHW5 |
| Nme2 | -0,496498942 | 0,000331672 | P19804 |
| Dnajc5 | 0,496281822 | 0,000333988 | P60905 |
| Ces1c | 0,495603086 | 0,000341322 | P10959 |
| Rpl26 | 0,495521903 | 0,000342209 | P12749 |
| Arl8b | 0,49549452 | 0,000342509 | Q66HA6 |
| Clpx | -0,495182783 | 0,000345937 | Q5U2U0 |
| Rpl35 | 0,495128505 | 0,000346537 | P17078 |
| Hdac1 | 0,494868035 | 0,00398431 | Q4QQW4 |
| Pyurf | -0,494868035 | 0,00398431 | Q5U1Z8 |
| Cct2 | 0,494789405 | 0,000350306 | Q5XIM9 |
| Cspg4 | 0,494694277 | 0,000351371 | Q00657 |
| Rnaseh2a | -0,49454679 | 0,00401167 | Q5U209 |
| Adhfe1 | -0,494355189 | 0,000355188 | Q4QQW3 |
| Cryz | -0,494117647 | 0,051723442 | Q6AYT0 |
| Tbpl2 | 0,494117647 | 0,051723442 | A6H909 |
| Stx7 | 0,493554428 | 0,000364351 | O70257 |
| Atp5if1 | -0,492835432 | 0,00037276 | Q03344 |
| Dstn | 0,492292662 | 0,000379224 | Q7M0E3 |
| Rpsa | 0,491532783 | 0,000388444 | P38983 |
| Acot7 | -0,491355681 | 0,000390621 | Q64559 |
| Msrb2 | -0,491202346 | 0,004306249 | Q4FZX5 |
| Dync1i2 | 0,491176471 | 0,053350436 | Q62871 |
| Pigu | 0,490772905 | 0,000397865 | Q8CHJ1 |
| Dld | -0,490460553 | 0,000401797 | Q6P6R2 |
| Slmap | 0,490447243 | 0,000401966 | P0C219 |
| Tap2 | 0,490102639 | 0,00440709 | P36372 |
| Mrpl30 | -0,489062586 | 0,00041983 | P0C2C1 |
| Mrps7 | -0,488940755 | 0,000421436 | Q5I0K8 |
| Dhrs4 | -0,488723641 | 0,000424311 | Q8VID1 |
| Hnrnpa3 | 0,488235294 | 0,05501503 | Q6URK4 |
| Ptpn1 | 0,488235294 | 0,05501503 | P20417 |
| Bpi | 0,488131246 | 0,004592946 | Q6AXU0 |
| Lyrm9 | -0,487398041 | 0,004663768 | B2RZD7 |
| Cdh2 | -0,487271346 | 0,000444002 | Q9Z1Y3 |
| Pecam1 | 0,486864959 | 0,000449658 | Q3SWT0 |
| Bpnt1 | -0,486837107 | 0,000450048 | Q9Z1N4 |
| Trap1 | -0,486741758 | 0,000451385 | Q5XHZ0 |
| Ppif | -0,486511427 | 0,000454631 | P29117 |
| Arcn1 | 0,486429272 | 0,000455794 | Q66H80 |
| Tefm | -0,486172552 | 0,000459444 | Q4KM51 |
| Gmfg | 0,485294118 | 0,056717714 | Q80T18 |
| Rpl19 | 0,48514126 | 0,000474376 | P84100 |
| F12 | 0,484237537 | 0,00497988 | D3ZTE0 |
| Sel1l | 0,483945394 | 0,000492238 | Q80Z70 |
| Galnt1 | 0,483634588 | 0,000496979 | Q10473 |
| Lnpep | 0,483484871 | 0,000499277 | P97629 |
| Mme | 0,482352941 | 0,058458977 | P07861 |
| Map1b | 0,482352941 | 0,058458977 | P15205 |
| Mrps15 | -0,48188455 | 0,000524447 | Q5XI37 |
| Spp1 | 0,481588685 | 0,005258801 | P08721 |
| Atp6v1e1 | 0,481016094 | 0,00053858 | Q6PCU2 |
| Ckm | -0,480853258 | 0,000541268 | P00564 |
| Rab31 | 0,480785931 | 0,000542382 | Q6GQP4 |
| Mrpl11 | -0,480744701 | 0,000543066 | Q5XIE3 |
| Rps5 | 0,480445109 | 0,000548058 | P24050 |
| Prdx4 | 0,480241019 | 0,000551481 | Q9Z0V5 |
| Insr | 0,479007789 | 0,000572584 | P15127 |
| Vapa | 0,477854972 | 0,000592964 | Q9Z270 |
| Mrps31 | -0,477325156 | 0,000602547 | B0BN56 |
| Rps13 | 0,477270877 | 0,000603537 | P62278 |
| Amigo1 | -0,476470588 | 0,062059174 | Q80ZD7 |
| Metap2 | 0,476118107 | 0,000624903 | P38062 |
| Mapt | -0,475439883 | 0,005958306 | P19332 |
| Cct3 | 0,474924012 | 0,00064775 | Q6P502 |
| Snx1 | 0,473621363 | 0,000673528 | Q99N27 |
| Ruvbl1 | 0,473471382 | 0,000676555 | P60123 |
| Got1 | -0,47282004 | 0,00068984 | P13221 |
| Mt-atp6 | 0,472804259 | 0,000690165 | P05504 |
| Akr1b1 | -0,472778593 | 0,000690693 | P07943 |
| Lrpap1 | 0,472778593 | 0,000690693 | Q99068 |
| Ncam1 | 0,472752931 | 0,000691222 | P13596 |
| Ndrg2 | -0,472657204 | 0,000693198 | Q8VBU2 |
| Itpr2 | 0,472406309 | 0,064639943 | P29995 |
| Rab14 | 0,472005862 | 0,000706776 | P61107 |
| Trim28 | 0,47173447 | 0,000712504 | O08629 |
| Tsnax | 0,470588235 | 0,065819473 | Q9JHB5 |
| Nup155 | 0,46957478 | 0,006698146 | P37199 |
| Picalm | 0,469387755 | 0,000763797 | O55012 |
| Rps14 | 0,469346216 | 0,000764734 | P13471 |
| Mrpl10 | -0,468515908 | 0,000783681 | P0C2C4 |
| Mbnl1 | 0,467647059 | 0,06776085 | A0A8I6B1J2 |
| Pbxip1 | -0,467609304 | 0,00080485 | A2VD12 |
| Yars1 | 0,467593098 | 0,000805233 | Q4KM49 |
| Ssb | 0,467500747 | 0,000807419 | P38656 |
| Pigs | 0,466970635 | 0,000820069 | Q5XI31 |
| Ghitm | -0,466912769 | 0,000821461 | Q5XIA8 |
| Rps17 | 0,465709556 | 0,00085088 | P04644 |
| Selenok | 0,465600999 | 0,00085358 | P59798 |
| Ctsb | 0,465588363 | 0,000853895 | P00787 |
| Sardh | -0,465262701 | 0,000862044 | Q64380 |
| Dut | -0,464711282 | 0,007369714 | P70583 |
| Tars1 | 0,464705882 | 0,069743676 | Q5XHY5 |
| Mbl1 | 0,464202356 | 0,000889063 | P19999 |
| Ndrg4 | -0,463876677 | 0,000897512 | Q9Z2L9 |
| S100a8 | 0,463701251 | 0,000902093 | P50115 |
| Por | 0,463672142 | 0,000902855 | P00388 |
| Rps9 | 0,462507124 | 0,000933837 | P29314 |
| Smarca4 | 0,462331741 | 0,000938583 | Q8K1P7 |
| Fkbp8 | 0,461876833 | 0,007786905 | Q3B7U9 |
| Acly | 0,461552564 | 0,007835888 | P16638 |
| Rps15 | 0,461392321 | 0,000964371 | P62845 |
| Ankh | 0,460770212 | 0,000981796 | P58366 |
| Camlg | 0,460632943 | 0,072558801 | Q6DGG9 |
| Mdh1 | -0,459939206 | 0,001005512 | O88989 |
| Mrpl9 | -0,4590333 | 0,001031949 | Q641X9 |
| Filip1 | 0,458823529 | 0,073835534 | Q8K4T4 |
| Them4 | -0,458599072 | 0,001044839 | Q566R0 |
| Spryd7 | 0,457676337 | 0,001072711 | Q5M7T2 |
| Slk | 0,457580199 | 0,001075653 | O08815 |
| Rab21 | 0,457555363 | 0,001076414 | Q6AXT5 |
| Lypla1 | -0,45668693 | 0,001103334 | P70470 |
| Rheb | 0,456669835 | 0,00110387 | Q62639 |
| Cpn1 | 0,455882353 | 0,075945491 | Q9EQV8 |
| Gria3 | 0,455882353 | 0,075945491 | P19492 |
| Cdh8 | 0,455882353 | 0,075945491 | O54800 |
| Sigmar1 | 0,455830868 | 0,001130458 | Q9R0C9 |
| Rab10 | 0,455705138 | 0,001134491 | P35281 |
| G6pdx | 0,455645161 | 0,008775139 | P05370 |
| Serpina3k | 0,45562612 | 0,001137033 | P05545 |
| Rps7 | 0,455475643 | 0,001141887 | P62083 |
| Emc2 | 0,455384281 | 0,001144843 | B0BNG0 |
| Ssr3 | 0,455179526 | 0,001151492 | Q08013 |
| Rpl18 | 0,455011811 | 0,001156965 | P12001 |
| Gng5 | -0,454728045 | 0,001166277 | P63219 |
| Ap2m1 | 0,454565196 | 0,001171652 | P84092 |
| Lactb2 | -0,453889161 | 0,001194199 | Q561R9 |
| Bzw2 | -0,453684355 | 0,001201106 | Q9WTT7 |
| Uso1 | 0,453487308 | 0,009141221 | P41542 |
| Rps3a | 0,4532255 | 0,001216711 | P49242 |
| Pdia4 | 0,453087259 | 0,001221447 | P38659 |
| Cyp2d3 | -0,452941176 | 0,078098741 | P12938 |
| Bzw1 | 0,45271261 | 0,009275765 | Q6P7P5 |
| Prkcd | 0,452695001 | 0,001234977 | P09215 |
| Gaa | -0,452411323 | 0,001244844 | Q6P7A9 |
| Myo1b | 0,452344768 | 0,00124717 | Q05096 |
| Rpl22 | 0,451947348 | 0,001261135 | P47198 |
| Casq2 | -0,451826522 | 0,001265409 | P51868 |
| Atp6v1c1 | 0,451555122 | 0,001275055 | Q5FVI6 |
| Txnrd2 | -0,451150673 | 0,001289552 | Q9Z0J5 |
| Nudc | 0,450933565 | 0,001297395 | Q63525 |
| Eif5 | 0,450825011 | 0,001301332 | Q07205 |
| Atp5f1d | -0,450360963 | 0,001318283 | P35434 |
| Ccdc47 | 0,450264624 | 0,001321827 | Q5U2X6 |
| Rab11b | 0,450004584 | 0,009759322 | O35509 |
| Copg2 | -0,45 | 0,080295737 | D4ABY2 |
| Nucb2 | 0,449956578 | 0,001333215 | Q9JI85 |
| Coq3 | -0,449474803 | 0,001351201 | Q63159 |
| Sh3bp5 | -0,448352603 | 0,001393939 | Q91Y80 |
| Fahd1 | -0,448026924 | 0,001406565 | Q6AYQ8 |
| Pex13 | 0,447947214 | 0,010140787 | D4A2Y9 |
| Minpp1 | -0,447713395 | 0,001418817 | O35217 |
| Acad9 | -0,447333425 | 0,001433792 | B1WC61 |
| Pgam2 | -0,447321284 | 0,001434273 | P16290 |
| Mecp2 | 0,447200586 | 0,001439061 | Q00566 |
| Apmap | 0,446555568 | 0,001464889 | Q7TP48 |
| Pls3 | 0,446302078 | 0,001475152 | Q63598 |
| Cycs | -0,44609706 | 0,001483499 | P62898 |
| Rab6a | 0,445922108 | 0,001490656 | Q9WVB1 |
| Gys1 | -0,445843624 | 0,001493876 | A2RRU1 |
| Csde1 | 0,445381232 | 0,010634131 | P18395 |
| Acyp2 | -0,44527073 | 0,001517571 | P35745 |
| Hspa13 | 0,445083725 | 0,001525378 | O35162 |
| Tsfm | -0,444727916 | 0,001540331 | Q9QYU2 |
| Glud1 | -0,444432383 | 0,001552849 | P10860 |
| Dram2 | 0,444281525 | 0,010851677 | Q5BK09 |
| Myo5a | 0,444117647 | 0,084822744 | Q9QYF3 |
| Apom | 0,444117647 | 0,084822744 | P14630 |
| Rpl7 | 0,443370879 | 0,001598564 | P05426 |
| Prss23 | 0,443334781 | 0,001600139 | Q6AY61 |
| Lrrc8a | 0,443238256 | 0,001604359 | Q4V8I7 |
| Gsta3 | -0,442912585 | 0,001618668 | P04904 |
| Asb2 | -0,442855835 | 0,011139285 | Q5U2S6 |
| Ivd | -0,442490366 | 0,001637388 | P12007 |
| Vezt | 0,44244868 | 0,01122259 | Q5XI52 |
| Ptma | 0,44244868 | 0,01122259 | P06302 |
| Uggt1 | 0,442273246 | 0,00164709 | Q9JLA3 |
| Pxmp2 | 0,441645861 | 0,001675411 | Q07066 |
| Khdrbs1 | 0,441501223 | 0,086894042 | Q91V33 |
| Rab5a | 0,440741445 | 0,001716999 | M0RC99 |
| Tagln | -0,440210607 | 0,001741835 | P31232 |
| Adamts1 | 0,440198659 | 0,001742397 | Q9WUQ1 |
| Rpl17 | 0,439969605 | 0,001753215 | P24049 |
| Prss1 | 0,439842585 | 0,001759239 | P00762 |
| Myl6 | 0,439643943 | 0,001768698 | Q64119 |
| Hsd11b1 | 0,439275925 | 0,00178634 | P16232 |
| Dctn2 | -0,43914956 | 0,011917158 | Q6AYH5 |
| Myl2 | -0,43835319 | 0,001831266 | P08733 |
| Parva | 0,438235294 | 0,089530032 | Q9HB97 |
| Impa2 | -0,437605168 | 0,001868418 | Q8CIN7 |
| Bcl2l1 | 0,436802824 | 0,001909011 | P53563 |
| Atp6v1b2 | 0,436670557 | 0,001915778 | P62815 |
| Acadsb | -0,435868209 | 0,001957282 | P70584 |
| Entpd5 | -0,435584987 | 0,001972122 | Q6P6S9 |
| Itpr3 | 0,435294118 | 0,091952359 | Q63269 |
| Numb | 0,435294118 | 0,091952359 | Q2LC84 |
| Psip1 | 0,435117302 | 0,012814832 | Q812D1 |
| Maip1 | -0,433576682 | 0,002080247 | Q6AY04 |
| Adgrl2 | 0,433022145 | 0,002111014 | O88923 |
| Slc8a1 | 0,432483787 | 0,002141268 | Q01728 |
| Gfap | 0,432352941 | 0,094421038 | P47819 |
| Bag5 | 0,432352941 | 0,094421038 | Q5QJC9 |
| Lrrc8d | 0,432352941 | 0,094421038 | Q5U308 |
| Usp28 | 0,432352941 | 0,094421038 | D3ZJ96 |
| Hsd17b8 | -0,431894049 | 0,002174851 | Q6MGB5 |
| Bckdhb | -0,43182805 | 0,002178638 | P35738 |
| Prkra | -0,431674459 | 0,013625567 | Q4V8C7 |
| Ctsc | 0,429982631 | 0,002286926 | P80067 |
| Pdgfra | 0,429831465 | 0,002296004 | P20786 |
| Tjp1 | -0,429626012 | 0,002308394 | A0A0G2K2P5 |
| Hadhb | -0,429474828 | 0,002317548 | Q60587 |
| Hsp90aa1 | 0,429222753 | 0,002332883 | P82995 |
| Paics | 0,429040577 | 0,002344022 | P51583 |
| Mrpl24 | -0,429028932 | 0,002344735 | Q66H47 |
| RT1-B | 0,42888563 | 0,014313399 | P06341 |
| Acsf2 | -0,428866092 | 0,002354734 | Q499N5 |
| Huwe1 | 0,428172074 | 0,002397774 | P51593 |
| Prdx2 | -0,428148832 | 0,002399227 | P35704 |
| Rpl38 | 0,428117792 | 0,002401169 | P63174 |
| Timmdc1 | -0,427823161 | 0,002419673 | Q6AY94 |
| Nme1 | 0,427466415 | 0,002442247 | Q05982 |
| Lgmn | 0,42745853 | 0,014676465 | Q9R0J8 |
| Adprhl1 | -0,427280376 | 0,002454093 | Q5XIB3 |
| Cd59 | 0,426943118 | 0,002475697 | P27274 |
| Cdc42bpa | -0,426470588 | 0,099499112 | O54874 |
| Paf1 | 0,426470588 | 0,099499112 | Q4V886 |
| Impdh2 | 0,426470588 | 0,099499112 | E9PU28 |
| Ankrd1 | 0,426369213 | 0,002512847 | Q8R560 |
| Plcd1 | -0,425314807 | 0,002582387 | P10688 |
| Tnnt2 | -0,425109236 | 0,002596142 | P50753 |
| Epm2a | -0,424880591 | 0,002611516 | Q91XQ2 |
| Hspb6 | -0,424795094 | 0,002617285 | P97541 |
| Rps12 | 0,424120712 | 0,00266319 | P63324 |
| Abhd12 | 0,423980894 | 0,002672796 | Q6AYT7 |
| Fhl2 | 0,423872334 | 0,002680275 | O35115 |
| Psmd9 | -0,423852567 | 0,002681639 | Q9WTV5 |
| Tmem168 | 0,423841174 | 0,101830375 | Q5PQM0 |
| Hpcal1 | 0,423589438 | 0,002699853 | P62749 |
| Plod1 | -0,423529412 | 0,102109322 | Q63321 |
| Panx2 | -0,42239531 | 0,00278389 | P60571 |
| Plod3 | 0,421732523 | 0,002831523 | Q5U367 |
| Slc44a2 | 0,421321175 | 0,002861447 | B4F795 |
| Ttc19 | -0,420712677 | 0,002906224 | D4A6D7 |
| Exoc7 | 0,420588235 | 0,104767514 | O54922 |
| Azgp1 | 0,420588235 | 0,104767514 | Q63678 |
| Ermp1 | 0,420181296 | 0,00294583 | Q6UPR8 |
| Bcat2 | -0,420072736 | 0,00295398 | O35854 |
| Cnp | 0,419952778 | 0,002963009 | P13233 |
| Serpina3l | 0,41951855 | 0,002995895 | P05544 |
| Hadha | -0,418607176 | 0,003065966 | Q64428 |
| Kng1 | 0,418293467 | 0,017196264 | P08934 |
| Qars1 | 0,417684417 | 0,00313838 | Q66H61 |
| Trim35 | 0,417647059 | 0,107474079 | Q5RKG6 |
| Lrp2 | -0,417647059 | 0,107474079 | P98158 |
| Nadk2 | -0,417173252 | 0,00317914 | Q1HCL7 |
| Rps26 | 0,417155425 | 0,017532876 | P62856 |
| Tagln2 | 0,415621778 | 0,003305722 | Q5XFX0 |
| Serpine1 | 0,415524496 | 0,003313805 | P20961 |
| Rock1 | -0,41501115 | 0,109941147 | Q63644 |
| Mecr | -0,414644738 | 0,003387695 | Q9Z311 |
| Rps25 | 0,414253535 | 0,003421015 | P62853 |
| Ptgr1 | -0,413939098 | 0,003448005 | P97584 |
| Pgk1 | -0,413418741 | 0,003493083 | P16617 |
| Adh5 | -0,413342019 | 0,003499773 | P12711 |
| Abhd1 | 0,412071211 | 0,003612235 | Q5RK23 |
| Eno3 | -0,411767899 | 0,003639542 | P15429 |
| Uqcrh | 0,411528441 | 0,003661229 | Q5M9I5 |
| Pepd | 0,410596138 | 0,114161839 | Q5I0D7 |
| Rps11 | 0,41057374 | 0,003748824 | P62282 |
| Serpina6 | 0,410345483 | 0,003770038 | P31211 |
| Ap1b1 | 0,40958689 | 0,003841299 | P52303 |
| Hba1 | -0,409477027 | 0,003851717 | P01946 |
| Kpnb1 | 0,40935736 | 0,003863093 | P52296 |
| Ptpn11 | -0,409097077 | 0,003887938 | P41499 |
| Tbrg4 | -0,408945341 | 0,003902486 | Q5M9G9 |
| Asrgl1 | -0,408945103 | 0,020127299 | Q8VI04 |
| Ehd1 | -0,408717128 | 0,003924457 | Q641Z6 |
| Psap | 0,4082829 | 0,003966562 | P10960 |
| S100a6 | 0,407620495 | 0,004031556 | P05964 |
| Steap3 | 0,407577279 | 0,004035829 | Q5RKL5 |
| Nudt19 | -0,407490841 | 0,004044386 | Q6AYD9 |
| Chordc1 | 0,407478693 | 0,020622547 | D4A4T9 |
| Taldo1 | 0,406491709 | 0,004144465 | Q9EQS0 |
| Timm17a | -0,406339902 | 0,00415986 | O35092 |
| Cd9 | 0,406274595 | 0,004166498 | P40241 |
| Bad | 0,405882353 | 0,118791685 | O35147 |
| Ciapin1 | -0,405882353 | 0,118791685 | Q5XID1 |
| Creb1 | 0,405882353 | 0,118791685 | P15337 |
| H1-0 | 0,405514696 | 0,004244423 | P43278 |
| Cd1d | 0,404005863 | 0,004402944 | Q63493 |
| Pon2 | 0,403843023 | 0,004420358 | Q6AXM8 |
| Hmgb2 | 0,403712549 | 0,004434355 | P52925 |
| Rpl21 | 0,40356067 | 0,004450697 | P20280 |
| Sh3kbp1 | -0,403386887 | 0,00446946 | Q925Q9 |
| Yme1l1 | 0,4033764 | 0,004470595 | Q925S8 |
| F13a1 | 0,403343556 | 0,004474149 | O08619 |
| Usp19 | 0,402941176 | 0,121745763 | Q6J1Y9 |
| Rps16 | 0,402399308 | 0,00457741 | P62250 |
| Dlg1 | -0,4021261 | 0,022516579 | Q62696 |
| Fars2 | -0,401823758 | 0,00464137 | Q6AYQ3 |
| Rpl4 | 0,401498087 | 0,004677906 | P50878 |
| Ccn1 | 0,401498087 | 0,004677906 | Q9ES72 |
| Naga | -0,401357038 | 0,004693808 | Q66H12 |
| Man1b1 | -0,4 | 0,124750424 | B2GUY0 |
| Slc35a3 | 0,399926686 | 0,023335339 | Q6AXR5 |
| Erlin2 | 0,399869732 | 0,004864389 | B5DEH2 |
| Selenos | 0,399261832 | 0,004935656 | Q8VHV8 |
| Vwa5a | 0,398863534 | 0,023739812 | Q75WE7 |
| Tpm4 | 0,398697247 | 0,00500266 | P09495 |
| Supv3l1 | -0,398686425 | 0,005003952 | Q5EBA1 |
| Naxd | -0,397198929 | 0,005184349 | D4AAT7 |
| Nmes1 | 0,397058824 | 0,127806009 | Q5RK28 |
| Dock9 | 0,397058824 | 0,127806009 | Q63603 |
| Pi4k2a | 0,396992971 | 0,005209769 | Q99M64 |
| Faah | 0,395747413 | 0,024958689 | P97612 |
| Psmc1 | 0,395690287 | 0,005373086 | P62193 |
| Dnajb11 | 0,394984666 | 0,005463402 | Q6TUG0 |
| Akt1 | 0,394767552 | 0,005491457 | P47196 |
| Ywhab | 0,394441881 | 0,005533775 | P35213 |
| Npr2 | -0,394428152 | 0,025489978 | P16067 |
| Ctnnb1 | -0,393855507 | 0,005610686 | Q9WU82 |
| Atp2b4 | 0,393695015 | 0,025789215 | Q64542 |
| Eif3i | 0,393627704 | 0,005640816 | B0BNA7 |
| Ap1m1 | 0,39359479 | 0,005645181 | Q32Q06 |
| Gripap1 | -0,392961877 | 0,026091328 | Q9JHZ4 |
| Rpl37 | 0,392769908 | 0,005755541 | P61928 |
| Dnaja1 | 0,392422927 | 0,005802522 | P63036 |
| Pabpc1 | 0,391880156 | 0,005876683 | Q9EPH8 |
| Phyh | -0,391781125 | 0,005890303 | P57093 |
| Kcnj3 | 0,391176471 | 0,134071286 | P63251 |
| GLTP | 0,391129032 | 0,026859324 | B0BNM9 |
| Ubl3 | -0,391097844 | 0,005985027 | Q5BJT2 |
| Hmox2 | 0,390479551 | 0,006071882 | P23711 |
| Grn | 0,390172377 | 0,027267475 | P23785 |
| Capn2 | 0,390001629 | 0,006139768 | Q07009 |
| Mavs | -0,389992747 | 0,135357079 | Q66HG9 |
| Vdac3 | 0,389567389 | 0,006202022 | Q9R1Z0 |
| Erp29 | 0,389567389 | 0,006202022 | P52555 |
| Mt-nd4l | 0,389296188 | 0,027645736 | P05507 |
| Syp | -0,38856305 | 0,02796553 | P07825 |
| Sfxn3 | 0,388189318 | 0,006403238 | Q9JHY2 |
| Pak3 | 0,387829912 | 0,028288342 | Q62829 |
| Ptges3 | 0,387494233 | 0,006506867 | P83868 |
| Adrm1 | 0,38742944 | 0,006516601 | Q9JMB5 |
| Tnni3 | -0,387277119 | 0,006539534 | P23693 |
| Mrpl40 | -0,386765651 | 0,028762366 | P83565 |
| Ccdc60 | -0,386734334 | 0,006621824 | Q3ZAV0 |
| Nucks1 | -0,386561007 | 0,006648291 | Q9EPJ0 |
| Cdc42bpb | 0,385294118 | 0,140544177 | Q7TT49 |
| Ces1d | -0,384824142 | 0,006918625 | P16303 |
| Smim20 | -0,384095537 | 0,007034841 | C0HLM6 |
| Pfkl | 0,383857573 | 0,007073162 | P30835 |
| Carhsp1 | 0,383832831 | 0,030102316 | Q9WU49 |
| Timm8a | -0,383630048 | 0,007109972 | Q9WVA1 |
| Gas6 | -0,383586181 | 0,007117088 | Q63772 |
| Fhit | -0,383586181 | 0,007117088 | Q9JIX3 |
| Rps27 | 0,382870169 | 0,007234115 | Q71TY3 |
| Mrpl42 | -0,38276967 | 0,007250673 | P0C2B9 |
| Myo1c | 0,382640324 | 0,007272033 | Q63355 |
| Ptprs | -0,38235853 | 0,007318758 | Q64605 |
| Fbln5 | -0,382352941 | 0,14385925 | Q9WVH8 |
| Gpi | -0,380991153 | 0,007549198 | Q6P6V0 |
| Rps8 | 0,380926534 | 0,007560242 | P62243 |
| St3gal5 | -0,38052329 | 0,007629477 | Q68G12 |
| Bcr | 0,380498534 | 0,03168706 | F1LXF1 |
| Abhd16a | 0,380391419 | 0,007652237 | Q6MG55 |
| Ap2b1 | 0,380156318 | 0,007692961 | P62944 |
| Pccb | -0,379840964 | 0,00774788 | P07633 |
| Amacr | -0,379569572 | 0,007795415 | P70473 |
| Tfam | -0,379417034 | 0,007822244 | Q91ZW1 |
| Serpina3m | 0,379411765 | 0,147227134 | Q63556 |
| Csnk1a1 | 0,379111934 | 0,007876144 | P97633 |
| Eif4a2 | 0,378755394 | 0,007939539 | Q5RKI1 |
| Fbl | 0,378665689 | 0,032586684 | P22509 |
| Ppia | 0,37853828 | 0,00797836 | P10111 |
| Trdn | -0,378491362 | 0,007986771 | Q9QX75 |
| Akr1c15 | -0,378439994 | 0,007995988 | D3ZF77 |
| Naprt | -0,378185158 | 0,032825947 | Q6XQN1 |
| Sord | -0,377967191 | 0,032934945 | P27867 |
| Creld1 | 0,377625794 | 0,008143316 | Q4V7F2 |
| Cygb | -0,376832845 | 0,033506949 | Q921A4 |
| Tmlhe | -0,376470588 | 0,150648115 | Q91ZW6 |
| Mthfd2l | -0,376470588 | 0,150648115 | D3ZUA0 |
| Tmsb4x | 0,376470588 | 0,150648115 | P62329 |
| Gsta4 | -0,37636714 | 0,00837569 | P14942 |
| Tpm1 | -0,376095747 | 0,00842654 | P04692 |
| Aldh2 | -0,376031264 | 0,008438661 | P11884 |
| Cox6a2 | -0,37592271 | 0,0084591 | P10817 |
| Esam | 0,375770076 | 0,008487911 | Q6AYD4 |
| Mcm8 | -0,375733138 | 0,034069156 | D3ZVK1 |
| D2hgdh | -0,375552962 | 0,00852904 | P84850 |
| Fut8 | 0,375118734 | 0,008611814 | Q6EV76 |
| Gpc1 | -0,37490162 | 0,008653461 | P35053 |
| Slc28a2 | 0,374728615 | 0,008686771 | Q62773 |
| Myo1e | 0,373529412 | 0,15412247 | Q63356 |
| Abhd10 | -0,373529412 | 0,15412247 | Q5I0K5 |
| Crip2 | -0,37311043 | 0,009003738 | P36201 |
| Reep5 | 0,372839037 | 0,009057864 | B2RZ37 |
| Bnip1 | 0,372666088 | 0,009092502 | Q8VHI8 |
| Mrpl3 | -0,371753467 | 0,009277184 | P18665 |
| Tamm41 | -0,371482075 | 0,009332725 | D3ZKT0 |
| Actn1 | -0,371437877 | 0,009341797 | Q9Z1P2 |
| Tecr | 0,37103385 | 0,009425083 | Q64232 |
| Rpl6 | 0,371003637 | 0,009431336 | P21533 |
| Vcl | -0,370949357 | 0,009442581 | P85972 |
| Spon1 | 0,370588235 | 0,157650465 | P35446 |
| Coq7 | -0,370569397 | 0,009521614 | Q63619 |
| Gfm2 | -0,369962277 | 0,009649083 | Q5BJP6 |
| Aldoa | -0,368526764 | 0,009956339 | P05065 |
| Napa | 0,368408198 | 0,00998209 | P54921 |
| Igfals | 0,36840176 | 0,038015607 | P35859 |
| Ehd2 | -0,367993924 | 0,010072513 | Q4V8H8 |
| Itga7 | 0,367973946 | 0,010076892 | Q63258 |
| Mtarc2 | 0,367919665 | 0,010088796 | O88994 |
| Lamb2 | -0,367346939 | 0,010215141 | P15800 |
| Pgp | -0,367139794 | 0,010261172 | D3ZDK7 |
| Suox | -0,36584704 | 0,010552495 | Q07116 |
| Ncdn | 0,365835777 | 0,039480973 | O35095 |
| Fam136a | -0,365422718 | 0,010649653 | B0BN94 |
| Czib | -0,365102639 | 0,0399079 | Q498R7 |
| Ptgr2 | -0,36487 | 0,010777363 | Q5BK81 |
| Prkce | 0,364705882 | 0,164868397 | P09216 |
| Pter | -0,364705882 | 0,164868397 | Q63530 |
| Mri1 | -0,364705882 | 0,164868397 | Q5HZE4 |
| Sdhaf2 | -0,364705882 | 0,164868397 | Q5RJQ7 |
| Syncrip | 0,364425869 | 0,010880935 | Q7TP47 |
| Abcc9 | -0,364228526 | 0,01092723 | Q63563 |
| Pdcd6 | -0,363828806 | 0,011021518 | G3V7W1 |
| Clpb | -0,363438993 | 0,011114142 | Q9WTT2 |
| Tprg1l | 0,363269795 | 0,040991487 | A8WCF8 |
| Cavin4 | -0,362101721 | 0,01143699 | B1PRL5 |
| Hspa8 | 0,361830321 | 0,011503485 | P63018 |
| Idh1 | -0,361450361 | 0,011597135 | P41562 |
| Ssrp1 | 0,360300703 | 0,01188449 | Q04931 |
| Snx5 | 0,359052297 | 0,012203418 | B1H267 |
| Stub1 | 0,358823529 | 0,172303863 | A6HD62 |
| Pomgnt1 | 0,358823529 | 0,172303863 | Q5XIN7 |
| Slc25a22 | 0,357867883 | 0,012512748 | A0A0G2K5L2 |
| Mpi | -0,356337642 | 0,045305503 | Q68FX1 |
| Septin9 | 0,356239484 | 0,012948973 | Q9QZR6 |
| Baiap2 | 0,355882353 | 0,176103738 | Q6GMN2 |
| Igh-1a | 0,354863222 | 0,013327732 | P20761 |
| Micos10 | 0,354502525 | 0,013428551 | B2RYW8 |
| Dsp | -0,354320452 | 0,013479689 | F1LMV6 |
| Ptp4a1 | 0,354295021 | 0,013486845 | Q78EG7 |
| Cyp4f6 | 0,354202965 | 0,046704678 | P51871 |
| Yipf4 | 0,353739003 | 0,047013298 | Q5M7T4 |
| Tor1aip2 | 0,353588124 | 0,047114011 | Q6P752 |
| Gstz1 | -0,353570169 | 0,013692177 | P57113 |
| Map2k1 | 0,353372434 | 0,047258283 | Q01986 |
| Eif1a | 0,353298776 | 0,01376974 | Q6VV72 |
| Fxyd1 | -0,352941176 | 0,179958658 | O08589 |
| Pigbos1 | -0,352941176 | 0,179958658 | C0HLN0 |
| Ddah2 | -0,352286607 | 0,01406233 | Q6MG60 |
| Prkab2 | -0,352272727 | 0,047999354 | Q9QZH4 |
| Mrpl41 | -0,351715154 | 0,014229852 | Q5BJX1 |
| Nap1l1 | 0,351280938 | 0,014358278 | Q9Z2G8 |
| Glod4 | -0,350939097 | 0,014460077 | Q5I0D1 |
| Ncl | 0,350195397 | 0,014683674 | P13383 |
| S100a9 | 0,35 | 0,183868822 | P50116 |
| Dkc1 | 0,349372195 | 0,049998468 | P40615 |
| Pnp | 0,349301127 | 0,014956437 | P85973 |
| Map4 | -0,348586007 | 0,015177652 | Q5M7W5 |
| Snrnp200 | 0,348467989 | 0,015214426 | F1LNJ2 |
| Ap2a2 | 0,348215499 | 0,015293356 | P18484 |
| Lias | -0,347539182 | 0,051295592 | Q5XIH4 |
| Galns | 0,347058824 | 0,18783442 | Q32KJ6 |
| Mrps25 | -0,346577648 | 0,015813867 | Q4QR80 |
| Fdx1 | 0,346407625 | 0,052109582 | P24483 |
| Ddx4 | 0,346396005 | 0,01587251 | Q64060 |
| Nid2 | -0,346079735 | 0,015975059 | B5DFC9 |
| Wdr26 | 0,344972965 | 0,053156308 | F1LTR1 |
| Mrpl46 | -0,34465914 | 0,016442655 | Q5RK00 |
| Ube2d3 | 0,344117647 | 0,191855631 | P61078 |
| Kcnj2 | 0,344117647 | 0,191855631 | Q64273 |
| Mt-cyb | 0,344026489 | 0,016654606 | P00159 |
| Copg1 | 0,343475073 | 0,054266878 | Q4AEF8 |
| Rars1 | 0,343474367 | 0,016841469 | P40329 |
| Cntfr | -0,343094418 | 0,016971092 | Q08406 |
| Mink1 | 0,34196385 | 0,017361812 | F1LP90 |
| Scarb2 | 0,341900291 | 0,017384002 | P27615 |
| Eef1g | 0,34185529 | 0,017399728 | Q68FR6 |
| Abhd5 | 0,341176471 | 0,195932625 | Q6QA69 |
| Anxa5 | -0,34093253 | 0,017724858 | P14668 |
| Atp5f1a | -0,340490221 | 0,017882519 | P15999 |
| Pgm1 | -0,340389731 | 0,017918504 | P38652 |
| Myl4 | -0,340181844 | 0,017993141 | P17209 |
| Syngr1 | -0,339747592 | 0,0181499 | Q62876 |
| Ttgn1 | 0,339367622 | 0,018288011 | P19814 |
| Acbd5 | 0,339268268 | 0,018324271 | A0FKI7 |
| Smpx | -0,338933371 | 0,018446941 | Q925F0 |
| Lonp2 | 0,338281993 | 0,018687525 | Q3MIB4 |
| Eif2b5 | 0,338235294 | 0,200065559 | Q64350 |
| Stx12 | 0,337675732 | 0,018913825 | G3V7P1 |
| Acox1 | -0,336526719 | 0,019349078 | P07872 |
| Clic5 | 0,33576682 | 0,019641557 | Q9EPT8 |
| Clstn1 | 0,335294118 | 0,204254581 | Q6Q0N0 |
| Rpl36a | 0,335242232 | 0,019845637 | P83883 |
| Tpcn1 | 0,335006921 | 0,01993776 | Q9WTN5 |
| Fundc1 | -0,334800169 | 0,061064497 | Q5BJS4 |
| Pgm5 | -0,334581773 | 0,020105118 | D3ZVR9 |
| Eif4a3 | 0,334572693 | 0,020108705 | Q3B8Q2 |
| Calr | 0,333975629 | 0,020345772 | P18418 |
| Eif2s3 | 0,333944282 | 0,061769868 | P81795 |
| Gdpd1 | 0,333803815 | 0,020414427 | Q0VGK4 |
| Slc25a29 | -0,33366807 | 0,020468807 | Q5HZE0 |
| Pde4b | -0,333559507 | 0,020512386 | P14646 |
| Map1lc3a | 0,333550454 | 0,020516024 | Q6XVN8 |
| C1galt1 | 0,333211144 | 0,06237915 | Q9JJ05 |
| Dmac2l | -0,33260964 | 0,020897023 | Q5XIM4 |
| Bgn | 0,332292995 | 0,021026588 | P47853 |
| Ssbp1 | -0,332039627 | 0,021130748 | P28042 |
| Letm1 | -0,331379255 | 0,021404271 | Q5XIN6 |
| Cand2 | -0,331261704 | 0,021453272 | Q9R0L4 |
| Ywhaz | 0,330619335 | 0,02172271 | P63102 |
| Ppp1r2 | -0,329654247 | 0,212444652 | P50411 |
| Mtm1 | -0,328602057 | 0,022587427 | Q6AXQ4 |
| Cbr1 | -0,328231016 | 0,022749584 | P47727 |
| Rusf1 | 0,327833261 | 0,022924501 | Q499P8 |
| Pnpla8 | -0,326874017 | 0,023350989 | D3ZRC4 |
| Abcd3 | 0,326494057 | 0,023521754 | P16970 |
| Serbp1 | 0,326470588 | 0,217159491 | Q6AXS5 |
| Vmp1 | 0,326470588 | 0,217159491 | Q91ZQ0 |
| Snap23 | 0,326276937 | 0,023619803 | O70377 |
| Hsp90ab1 | 0,325625577 | 0,023916011 | P34058 |
| Nlrx1 | 0,325073955 | 0,024169293 | Q5FVQ8 |
| Sdf4 | 0,324802562 | 0,024294727 | Q91ZS3 |
| Tppp3 | -0,323599653 | 0,024857287 | Q5PPN5 |
| Adgrf5 | -0,323529412 | 0,221574125 | Q9WVT0 |
| Mesd | 0,323529412 | 0,221574125 | Q5U2R7 |
| Nit2 | -0,323508658 | 0,024900282 | Q497B0 |
| Aldh1a1 | -0,323165436 | 0,025063015 | P51647 |
| Fkbp4 | 0,321320017 | 0,02595333 | Q9QVC8 |
| Wipi2 | 0,3209605 | 0,073274541 | Q6AY57 |
| Stx18 | 0,320747801 | 0,073475959 | Q68FW4 |
| Phf5a | 0,320588235 | 0,226045422 | P83871 |
| Txnrd1 | 0,320486377 | 0,026364101 | O89049 |
| Tmem41b | 0,32030614 | 0,026453622 | Q5FVN2 |
| Chchd4 | 0,320134611 | 0,026539053 | Q5BJN5 |
| Fzd1 | -0,320080332 | 0,026566135 | Q08463 |
| Chid1 | -0,320026054 | 0,026593239 | A0JPQ9 |
| Igg-2a | 0,319583152 | 0,026815274 | P20760 |
| Myh11 | -0,319174923 | 0,027021295 | Q63862 |
| Ago2 | 0,318966455 | 0,02712701 | Q9QZ81 |
| Pf4 | 0,318944186 | 0,075201228 | P06765 |
| Lrrc39 | -0,318548387 | 0,075583999 | D3ZXS4 |
| Ckb | -0,31835206 | 0,027440585 | P07335 |
| Rpl23a | 0,318115199 | 0,027562279 | P62752 |
| Usp7 | 0,317954842 | 0,027644922 | Q4VSI4 |
| Acsl1 | 0,317889426 | 0,027678694 | P18163 |
| Rps28 | 0,317800635 | 0,027724589 | P62859 |
| Arf6 | 0,317647059 | 0,230573464 | P62332 |
| Ide | -0,317429301 | 0,027917218 | P35559 |
| Ldha | -0,317329354 | 0,027969255 | P04642 |
| Nap1l4 | -0,317095015 | 0,028091581 | Q5U2Z3 |
| Prkaa1 | 0,316927872 | 0,077166927 | P54645 |
| Ctdnep1 | 0,316406971 | 0,077681151 | Q3B7T6 |
| Txndc15 | 0,316348974 | 0,077738569 | Q5BJT4 |
| Mcpt1 | -0,316326531 | 0,028495863 | P09650 |
| Gpd2 | 0,315909461 | 0,028717295 | P35571 |
| Tmem11 | 0,315900888 | 0,028721862 | B0BN86 |
| Hnrnpu | 0,315583782 | 0,0288912 | Q6IMY8 |
| Dbi | -0,315140989 | 0,029129046 | P11030 |
| Scfd1 | 0,314715302 | 0,029359237 | Q62991 |
| Alg13 | 0,314705882 | 0,235158319 | Q5I0K7 |
| Slc8a2 | 0,314516129 | 0,079570036 | P48768 |
| Gpx4 | 0,314381089 | 0,029541022 | P36970 |
| Dcaf8 | -0,314361655 | 0,079725901 | Q5U2M6 |
| Cryab | 0,314335342 | 0,029565978 | P23928 |
| Ubtf | 0,313683982 | 0,029923206 | P25977 |
| Etf1 | 0,313412583 | 0,030073106 | Q5U2Q7 |
| Plcb4 | -0,313349798 | 0,030107872 | Q9QW07 |
| P2rx5 | 0,313032623 | 0,030284013 | P51578 |
| Dcn | -0,312823993 | 0,030400341 | Q01129 |
| Ezr | 0,312552586 | 0,030552227 | P31977 |
| Dmd | -0,312064053 | 0,03082721 | P11530 |
| Adra1a | 0,311764706 | 0,239800048 | P43140 |
| Gimap4 | 0,311612136 | 0,082539761 | Q8K3K9 |
| Atp5po | -0,311555067 | 0,031115888 | Q06647 |
| Macrod1 | -0,311521239 | 0,031135153 | Q8K4G6 |
| Gpx1 | -0,311124379 | 0,031361906 | P04041 |
| Abra | 0,310790274 | 0,031553864 | Q8K4K7 |
| Dync1li1 | 0,310590024 | 0,031669382 | Q9QXU8 |
| Farp1 | 0,310117302 | 0,084101291 | F1LYQ8 |
| Rpl36 | 0,309821695 | 0,032115867 | P39032 |
| Eif2s1 | 0,309667264 | 0,032206235 | P68101 |
| Psmd13 | 0,309496024 | 0,032306685 | B0BN93 |
| Atp5mc3 | 0,309241418 | 0,032456519 | Q71S46 |
| Arl1 | 0,309224632 | 0,032466417 | P61212 |
| Lap3 | -0,309120528 | 0,032527862 | Q68FS4 |
| Slc25a5 | 0,309015904 | 0,032589711 | Q09073 |
| Nhlrc1 | -0,308915729 | 0,032649022 | Q6IMG5 |
| Coq8b | -0,308823529 | 0,244498696 | Q6AY19 |
| Ptpn9 | 0,308823529 | 0,244498696 | Q641Z2 |
| Cdk17 | 0,308823529 | 0,244498696 | O35831 |
| Aif1 | 0,308823529 | 0,244498696 | P55009 |
| Gosr1 | 0,308284457 | 0,086046759 | Q62931 |
| Ppm1b | -0,307607705 | 0,086773751 | P35815 |
| Ppp1r12a | -0,30720799 | 0,033673945 | Q10728 |
| Gja1 | 0,306836378 | 0,033900462 | P08050 |
| Prkaca | -0,306818182 | 0,08762782 | P27791 |
| Chpt1 | 0,306193346 | 0,034295397 | Q66H21 |
| Cav1 | -0,305905341 | 0,034473511 | P41350 |
| Mgat5 | 0,305882353 | 0,249254299 | Q08834 |
| Ppp6c | 0,30550429 | 0,03472281 | Q64620 |
| Psmc3 | 0,305262301 | 0,034873953 | Q63569 |
| Rps23 | 0,305045187 | 0,03501002 | P62268 |
| Mat2a | 0,304928354 | 0,035083422 | P18298 |
| Fau | 0,304828073 | 0,035146526 | P62864 |
| Atp2a2 | 0,304347827 | 0,035450032 | P11507 |
| Pdia3 | 0,303967867 | 0,035691687 | P11598 |
| Tmod1 | -0,302974381 | 0,036329973 | P70567 |
| Gar1 | 0,302941176 | 0,254066881 | Q6AYA1 |
| Col5a1 | -0,302941176 | 0,254066881 | Q9JI03 |
| Vps33b | 0,302941176 | 0,254066881 | Q63616 |
| Trio | 0,302419355 | 0,092504556 | F1M0Z1 |
| Prxl2b | -0,302052786 | 0,092920097 | D3ZVR7 |
| Spg7 | -0,301734198 | 0,037139923 | Q7TT47 |
| Pdk4 | 0,301587733 | 0,037236552 | O54937 |
| Sparc | 0,301128962 | 0,03754056 | P16975 |
| Grb2 | 0,301020408 | 0,037612793 | P62994 |
| Faf2 | 0,3008033 | 0,0377576 | Q5BK32 |
| Glo1 | -0,300781676 | 0,037772048 | Q6P7Q4 |
| Eef1d | 0,30061406 | 0,094564778 | Q68FR9 |
| Rps6 | 0,300276828 | 0,038110653 | P62755 |
| Mpp7 | -0,3 | 0,258936453 | Q5U2Y3 |
| Ripor2 | 0,3 | 0,258936453 | Q7TP54 |
| Zc3h18 | 0,3 | 0,258936453 | Q6TQE1 |
| Nrp1 | 0,299145068 | 0,038878805 | Q9QWJ9 |
| Slc4a1 | 0,298965995 | 0,039001503 | P23562 |
| Hsp90b1 | 0,298919829 | 0,039033187 | Q66HD0 |
| Smim12 | -0,298439409 | 0,039364156 | D4ACP2 |
| Grin2d | -0,297912768 | 0,03972961 | Q62645 |
| Cyp4v2 | 0,297896595 | 0,039740877 | A2RRT9 |
| H1-4 | 0,297299499 | 0,040158682 | P15865 |
| Echdc3 | -0,297221016 | 0,040213866 | Q3MIE0 |
| Mtrf1l | -0,297058824 | 0,263863015 | Q4V7E5 |
| Vegfa | -0,297058824 | 0,263863015 | P16612 |
| Ephx1 | 0,296439042 | 0,040767098 | P07687 |
| Manf | 0,295980677 | 0,041094271 | P0C5H9 |
| Hspa4 | 0,294748271 | 0,04198462 | O88600 |
| Mthfd1 | 0,294677993 | 0,042035864 | P27653 |
| Ehhadh | -0,294569436 | 0,04211512 | P07896 |
| Akr1b7 | -0,294251751 | 0,042347761 | Q5RJP0 |
| Fbxo6 | -0,294197471 | 0,042387615 | Q923V4 |
| Prkar2a | -0,293964394 | 0,042559097 | P12368 |
| Rack1 | 0,293879768 | 0,042621499 | P63245 |
| Col1a1 | -0,29385584 | 0,042639157 | P02454 |
| Gdi2 | 0,293530178 | 0,042880075 | P50399 |
| Slc2a1 | 0,292987408 | 0,043284078 | P11167 |
| Pdap1 | 0,292862478 | 0,270990658 | Q62785 |
| Rabggta | 0,292732106 | 0,103970595 | Q08602 |
| Acss3 | -0,292661746 | 0,043527969 | A0A0G2K047 |
| Pdia5 | 0,29261541 | 0,043562761 | Q5I0H9 |
| Mt-nd3 | 0,291855511 | 0,044136595 | P05506 |
| Ptms | -0,291788856 | 0,105141816 | P04550 |
| Eif3d | 0,291638397 | 0,044301676 | Q6AYK8 |
| Taok1 | 0,291176471 | 0,273887048 | O88664 |
| Ecsit | -0,290878498 | 0,04488343 | Q5XIC2 |
| Vars2 | 0,28983934 | 0,045689045 | Q6MG21 |
| Txnl1 | 0,288614467 | 0,04665372 | Q920J4 |
| Rab9a | 0,288235294 | 0,27898446 | Q99P75 |
| Scn4b | -0,288123167 | 0,109787935 | Q7M730 |
| Sdhaf3 | -0,287776813 | 0,047322925 | Q6TUF2 |
| Lyrm2 | -0,287756598 | 0,110260879 | B2GV91 |
| Rps24 | 0,287358194 | 0,047660275 | P62850 |
| Arf3 | 0,286753331 | 0,048151161 | P61206 |
| Rpl30 | 0,286047711 | 0,048729001 | P62890 |
| Eif3b | 0,28582284 | 0,048914328 | Q4G061 |
| Nudt9 | -0,285294118 | 0,28413874 | Q5XIG0 |
| Rraga | 0,285294118 | 0,28413874 | Q63486 |
| Gfer | -0,284752755 | 0,049804092 | Q63042 |
| Arl6ip5 | 0,284589915 | 0,049940634 | Q9ES40 |
| Gls | 0,28425652 | 0,050221135 | P13264 |
| Aldh3a2 | 0,284047115 | 0,05039797 | P30839 |
| Tuba1c | 0,284000544 | 0,050437366 | Q6AYZ1 |
| Tomm20 | 0,282457664 | 0,051756707 | Q62760 |
| Cd48 | 0,28238743 | 0,051817422 | P10252 |
| Cnbp | 0,282352941 | 0,289349829 | P62634 |
| Snx27 | 0,282352941 | 0,289349829 | Q8K4V4 |
| Oasl | 0,282352941 | 0,289349829 | G3V645 |
| Pitpna | 0,28199213 | 0,052160229 | P16446 |
| Entpd1 | 0,280565582 | 0,053412619 | P97687 |
| Ywhaq | 0,280178029 | 0,05375702 | P68255 |
| Myadml2 | 0,280058651 | 0,120549058 | B2RZ87 |
| Gstm7 | -0,280022797 | 0,05389547 | P08009 |
| Timm10b | 0,279635258 | 0,054242367 | Q9R1B1 |
| Cyp4f5 | -0,279411765 | 0,294617654 | P51870 |
| Chgb | -0,279411765 | 0,294617654 | O35314 |
| Rplp2 | 0,279270478 | 0,054570536 | P02401 |
| Steap4 | 0,279216198 | 0,054619505 | Q4V8K1 |
| Armc10 | 0,279068507 | 0,054752924 | B1WBW4 |
| Sec31a | 0,278502999 | 0,055266215 | Q9Z2Q1 |
| Cat | -0,27833261 | 0,05542163 | P04762 |
| Wdr44 | -0,278145771 | 0,296902571 | Q9R037 |
| Dnajc3 | 0,278115502 | 0,055620167 | Q9R0T3 |
| Snu13 | 0,277960214 | 0,055762522 | P55770 |
| Npr3 | -0,277884705 | 0,123579596 | P41740 |
| Mff | 0,277750638 | 0,05595511 | Q4KM98 |
| Cacna2d1 | 0,277262118 | 0,056406111 | P54290 |
| Cops3 | -0,277138515 | 0,056520685 | Q68FW9 |
| Smpd2 | 0,277044999 | 0,056607493 | Q9ET64 |
| Pde2a | 0,276921407 | 0,056722384 | Q01062 |
| Bdh1 | -0,276781111 | 0,056853031 | P29147 |
| Tmem135 | 0,2766741 | 0,299571885 | Q5U4F4 |
| Dhx36 | 0,276470588 | 0,299942128 | D4A2Z8 |
| Marcks | 0,276052974 | 0,057534982 | P30009 |
| Slc4a4 | 0,276026393 | 0,126214555 | Q9JI66 |
| Abcd1 | 0,275897631 | 0,05768132 | D3ZHR2 |
| Cox5a | -0,275618758 | 0,057944776 | P11240 |
| Atxn10 | 0,275517681 | 0,058040503 | Q9ER24 |
| Scpep1 | 0,275510204 | 0,05804759 | Q920A6 |
| Sptbn2 | -0,273746517 | 0,059738647 | Q9QWN8 |
| Ppig | 0,273529412 | 0,305323156 | O55035 |
| Stk24 | 0,273529412 | 0,305323156 | B0LT89 |
| Armcx3 | 0,273529412 | 0,305323156 | Q5XID7 |
| Retreg1 | 0,273460411 | 0,129920871 | Q5FVM3 |
| Rpl5 | 0,27340824 | 0,060067464 | P09895 |
| Nid1 | -0,272687799 | 0,060772595 | P08460 |
| Cul3 | 0,272385667 | 0,131496824 | B5DF89 |
| Cpne1 | 0,272376921 | 0,061078907 | D4A1R8 |
| Ndrg3 | 0,272268361 | 0,061186164 | Q6AYR2 |
| Rpl13 | 0,2719353 | 0,061516166 | P41123 |
| Dpp3 | 0,271718186 | 0,061732053 | O55096 |
| Asah1 | -0,271663908 | 0,061786119 | Q6P7S1 |
| Mtor | -0,271555351 | 0,061894365 | P42346 |
| Ap2s1 | 0,270904009 | 0,062547033 | P62744 |
| Xpo1 | 0,270733825 | 0,062718466 | Q80U96 |
| Plbd1 | -0,270524059 | 0,06293029 | Q5U2V4 |
| Slc29a1 | -0,27014411 | 0,063315422 | O54698 |
| Atp5mg | -0,270089831 | 0,063370594 | Q6PDU7 |
| Cops2 | -0,269601324 | 0,063868874 | P61203 |
| Nampt | -0,269500082 | 0,063972533 | Q80Z29 |
| Dmac2 | -0,267647059 | 0,316254419 | Q5I0I4 |
| Dtnb | -0,267647059 | 0,316254419 | P84060 |
| Ccs | 0,267647059 | 0,316254419 | Q9JK72 |
| Rnf114 | -0,267647059 | 0,316254419 | Q6J2U6 |
| Ctrb1 | 0,267595308 | 0,13869246 | P07338 |
| Ociad1 | -0,26759302 | 0,065950317 | Q5XIG4 |
| Hnrnpk | 0,266995956 | 0,066579438 | P61980 |
| Matr3 | 0,266568963 | 0,067032282 | P43244 |
| Mapre1 | 0,266361141 | 0,140592057 | Q66HR2 |
| Calm3 | -0,265414677 | 0,068268726 | P0DP31 |
| Bag6 | 0,264705882 | 0,321804396 | Q6MG49 |
| Slc25a20 | 0,264003474 | 0,069804929 | P97521 |
| Tubb4b | -0,26395636 | 0,069856685 | Q6P9T8 |
| Tep1 | 0,263467854 | 0,070395125 | O08653 |
| Slc25a4 | 0,262979347 | 0,070936851 | Q05962 |
| Git1 | -0,261764706 | 0,327410412 | Q9Z272 |
| Ilf2 | 0,261764706 | 0,327410412 | Q7TP98 |
| Psmd2 | 0,261310898 | 0,072812028 | Q4FZT9 |
| Ndufaf5 | -0,26069965 | 0,073508748 | B2GV71 |
| Kars1 | 0,259132606 | 0,075319008 | Q5XIM7 |
| Apoa2 | 0,259014051 | 0,332703855 | P04638 |
| Uba1 | 0,258922514 | 0,07556436 | Q5U300 |
| Alg6 | 0,258823529 | 0,333072307 | Q3T1L5 |
| Tmem109 | -0,25875967 | 0,075754968 | Q6AYQ4 |
| Eml2 | -0,257999729 | 0,076649492 | Q6P6T4 |
| Trim26 | 0,257775607 | 0,07691489 | P62603 |
| Plekhf1 | -0,25754238 | 0,335556004 | Q68FU1 |
| Pygm | -0,257225826 | 0,077568989 | P09812 |
| Rps21 | 0,255882353 | 0,338789908 | P05765 |
| Dars1 | 0,255760306 | 0,079333997 | P15178 |
| Cxadr | 0,255543192 | 0,079598144 | Q9R066 |
| Ahcy | 0,255488914 | 0,079664289 | P10760 |
| Camk2d | -0,254511901 | 0,080862294 | P15791 |
| Tmed1 | -0,254077673 | 0,081399259 | Q5BK85 |
| Lmnb1 | 0,253162241 | 0,162107125 | P70615 |
| Txnip | 0,252941176 | 0,344563031 | Q5M7W1 |
| Btd | 0,252941176 | 0,344563031 | Q5FVF9 |
| Tfb2m | 0,252941176 | 0,344563031 | Q5U2T7 |
| Dek | 0,25142485 | 0,084740741 | Q6AXS3 |
| Cox5b | -0,251302649 | 0,084897211 | P12075 |
| Mrpl14 | -0,251122721 | 0,165631065 | Q7M0E7 |
| Sptan1 | -0,250820962 | 0,085516179 | P16086 |
| Stx4 | 0,250441013 | 0,086006898 | Q08850 |
| Dpep1 | 0,250434216 | 0,086015696 | P31430 |
| Armt1 | -0,250400327 | 0,086059576 | Q6AYT5 |
| Vim | 0,250366569 | 0,166951359 | P31000 |
| Twf1 | 0,250022914 | 0,167553879 | Q5RJR2 |
| Aco1 | -0,249911793 | 0,086694068 | Q63270 |
| Synj2bp | 0,249674338 | 0,087003784 | Q9WVJ4 |
| Prepl | 0,249633431 | 0,168238618 | Q5HZA6 |
| Acad11 | 0,249484314 | 0,087252256 | B3DMA2 |
| Hp1bp3 | 0,249260416 | 0,08754573 | Q6P747 |
| Ndufs1 | 0,248656571 | 0,088341061 | Q66HF1 |
| Pacsin2 | -0,248548011 | 0,088484642 | Q9QY17 |
| Srsf5 | 0,247800587 | 0,171487661 | Q09167 |
| Ctsd | -0,247570971 | 0,089785067 | P24268 |
| Gpm6b | -0,24620729 | 0,09162492 | Q9JJK1 |
| Slc44a1 | 0,245769014 | 0,358872518 | Q8VII6 |
| Uimc1 | -0,245013163 | 0,093259953 | Q5PQK4 |
| Timm10 | -0,244958884 | 0,093334807 | P62074 |
| Plec | -0,244748413 | 0,093625504 | P30427 |
| Dnm2 | 0,244572297 | 0,093869288 | P39052 |
| Mvp | 0,244246635 | 0,094321375 | Q62667 |
| Micall1 | -0,244117647 | 0,362213496 | D3ZQL6 |
| Nedd8 | 0,244117647 | 0,362213496 | Q71UE8 |
| Mgp | 0,244117647 | 0,362213496 | P08494 |
| Srsf6 | 0,2436562 | 0,095145324 | G3V6S8 |
| Sh3glb2 | 0,242825673 | 0,364839381 | Q5PPJ9 |
| Taok3 | -0,241817981 | 0,366894781 | Q53UA7 |
| Ogfr | 0,241274494 | 0,098525693 | Q9QXY4 |
| Acox3 | -0,241226762 | 0,098594375 | Q63448 |
| Cast | 0,241202346 | 0,183552797 | P27321 |
| Opcml | -0,241176471 | 0,368206608 | P32736 |
| Vps33a | -0,241176471 | 0,368206608 | Q63615 |
| Timm13 | -0,240990013 | 0,09893558 | P62076 |
| Ppp3ca | 0,240616604 | 0,099475592 | P63329 |
| F11r | 0,240562325 | 0,099554276 | Q9JHY1 |
| Exoc3 | -0,240329977 | 0,185191499 | Q62825 |
| Nqo2 | -0,240182376 | 0,100106414 | Q6AY80 |
| Txn | 0,24003257 | 0,100324758 | P11232 |
| Gstm2 | -0,239754655 | 0,100730794 | P08010 |
| Tnni3k | -0,23957881 | 0,100988358 | Q7TQP6 |
| Kyat1 | -0,239574742 | 0,186618438 | Q08415 |
| Nceh1 | 0,239550539 | 0,101029815 | B2GV54 |
| Dnajc25 | 0,238825413 | 0,102097623 | Q5BJW9 |
| Eef1a2 | -0,238777615 | 0,102168313 | P62632 |
| Scamp4 | 0,238235294 | 0,37425413 | Q9ET20 |
| Oxr1 | -0,238235294 | 0,37425413 | Q4V8B0 |
| Aldh16a1 | 0,238187 | 0,103044909 | Q3T1L0 |
| Stip1 | 0,237794122 | 0,103631218 | O35814 |
| Col1a2 | -0,237522729 | 0,104037723 | P02466 |
| Sars1 | 0,237034223 | 0,104772517 | Q6P799 |
| Cops4 | -0,235670864 | 0,106844311 | Q68FS2 |
| Slc4a3 | -0,235337243 | 0,194767782 | P23348 |
| Gdi1 | -0,235255802 | 0,107481241 | P50398 |
| Rab3a | -0,235025918 | 0,107835257 | P63012 |
| Prkar1a | -0,234754526 | 0,108254343 | P09456 |
| Ppp1cb | 0,234537412 | 0,108590508 | P62142 |
| Ampd3 | 0,234368215 | 0,108853033 | O09178 |
| Gde1 | 0,233870968 | 0,197644466 | Q9JL55 |
| Eno1 | -0,233723234 | 0,109858243 | P04764 |
| Rock2 | 0,233159198 | 0,199051469 | Q62868 |
| Get3 | 0,232481138 | 0,111814025 | G3V9T7 |
| Mt-nd1 | 0,231618945 | 0,113187156 | P03889 |
| Psmd11 | 0,230683638 | 0,114691216 | F1LMZ8 |
| Mrc2 | 0,230571848 | 0,204224524 | Q4TU93 |
| Lsamp | -0,230568823 | 0,114876891 | Q62813 |
| Ncs1 | 0,229411765 | 0,392720491 | P62168 |
| Phka1 | -0,229411765 | 0,392720491 | Q64649 |
| Pik3c3 | -0,229411765 | 0,392720491 | O88763 |
| Ak1 | -0,229278619 | 0,116979127 | P39069 |
| Marcksl1 | 0,229128218 | 0,117226077 | Q9EPH2 |
| Slc25a34 | 0,228566776 | 0,118151426 | Q5XIF9 |
| Lss | 0,228108965 | 0,395487891 | P48450 |
| Ufsp2 | 0,22765022 | 0,119673952 | Q5XIB4 |
| Timm9 | 0,227155535 | 0,120501842 | Q9WV97 |
| Rab13 | 0,226560353 | 0,212427163 | P35286 |
| B2m | 0,226539589 | 0,212470199 | P07151 |
| Phldb1 | -0,226470588 | 0,398982927 | Q63312 |
| Ripor1 | 0,226335215 | 0,12188425 | Q4FZU8 |
| Poglut3 | -0,226010449 | 0,213568942 | Q566E5 |
| Osbpl1a | -0,225900999 | 0,122620828 | Q8K4M9 |
| Ca2 | -0,225575337 | 0,123175465 | P27139 |
| Dpysl2 | -0,22543086 | 0,123422129 | P47942 |
| Slc25a13 | -0,22505089 | 0,124072631 | F1LZW6 |
| Ndufs6 | 0,224936221 | 0,124269448 | P52504 |
| Flt1 | 0,224381305 | 0,216976212 | P53767 |
| Mapk14 | 0,224254929 | 0,125443679 | P70618 |
| Strap | 0,22406166 | 0,125778303 | Q5XIG8 |
| Cttn | 0,223693953 | 0,404943643 | Q66HL2 |
| Ubr5 | 0,223529412 | 0,405298347 | Q62671 |
| Rmdn2 | -0,223529412 | 0,405298347 | Q498D5 |
| Acy1a | -0,223240469 | 0,219384186 | Q6AYS7 |
| Gtf2f1 | 0,222596141 | 0,128337602 | Q6AY96 |
| Vps26a | 0,222535823 | 0,12844377 | Q6AY86 |
| P3h2 | -0,222222282 | 0,408121994 | Q4KLM6 |
| Fabp5 | 0,222216191 | 0,129007469 | P55053 |
| Abcc4 | 0,222071438 | 0,129263365 | F1M3J4 |
| Timm23 | -0,221637174 | 0,130033346 | O35093 |
| Lancl1 | -0,221462303 | 0,130344376 | Q9QX69 |
| Slc27a1 | 0,221136623 | 0,130925124 | P97849 |
| Psmc4 | 0,220674487 | 0,224866565 | Q63570 |
| Nudt21 | 0,220511411 | 0,225218096 | Q4KM65 |
| St13 | -0,220153608 | 0,132689791 | P50503 |
| Chp1 | 0,220039079 | 0,132896541 | P61023 |
| Sccpdh | 0,219562504 | 0,133759456 | Q6AY30 |
| Actn4 | 0,219399664 | 0,134055262 | Q9QXQ0 |
| Ryr2 | -0,219351337 | 0,134143143 | B0LPN4 |
| Slc38a3 | -0,219188493 | 0,134439591 | Q9JHZ9 |
| Gsto1 | -0,218953539 | 0,134868174 | Q9Z339 |
| Hax1 | -0,217982468 | 0,136650348 | Q7TSE9 |
| Ggt5 | 0,217867998 | 0,136861583 | Q9QWE9 |
| Ascc3 | 0,217647059 | 0,418086842 | F1LPQ2 |
| Dctn1 | 0,217375367 | 0,232050771 | P28023 |
| Abcf1 | 0,217348823 | 0,137822692 | Q6MG08 |
| Csrp3 | 0,216172203 | 0,140019486 | P50463 |
| Isoc1 | -0,216112181 | 0,234841974 | Q6I7R3 |
| Ccdc51 | -0,215985888 | 0,14036972 | Q5PPN7 |
| Skp1 | -0,214834315 | 0,142548902 | Q6PEC4 |
| Arpc5l | 0,214705882 | 0,424559234 | A1L108 |
| Cyth1 | 0,214705882 | 0,424559234 | P97694 |
| Gclm | 0,214705882 | 0,424559234 | P48508 |
| Slc2a8 | 0,214705882 | 0,424559234 | Q9JJZ1 |
| Optn | -0,214705882 | 0,424559234 | Q8R5M4 |
| Rad23b | 0,214705882 | 0,424559234 | Q4KMA2 |
| Uqcc2 | -0,214611376 | 0,142973666 | B5DFN3 |
| Pds5b | 0,214526111 | 0,14313637 | Q6TRW4 |
| Vamp3 | 0,214471828 | 0,143240024 | P63025 |
| Inpp5a | -0,214297346 | 0,143573579 | D3ZZX1 |
| Nckap1 | 0,214025946 | 0,144093555 | P55161 |
| Pfkm | 0,213917386 | 0,144301935 | P47858 |
| Rtn3 | 0,213326097 | 0,145440836 | Q6RJR6 |
| Slc2a4 | -0,213015586 | 0,24177951 | P19357 |
| Aars2 | -0,211848997 | 0,148314982 | D3ZX08 |
| Tpmt | 0,211764706 | 0,431083247 | Q9Z0T0 |
| Pfdn2 | 0,211712951 | 0,244738371 | B0BN18 |
| Hsdl1 | 0,211697653 | 0,14861182 | Q4V8B7 |
| Entpd2 | -0,210811984 | 0,150357723 | O35795 |
| Galm | -0,210410557 | 0,247720696 | Q66HG4 |
| Adprh | 0,210410557 | 0,247720696 | Q02589 |
| Gadd45gip1 | -0,210043988 | 0,248564429 | Q5XJW2 |
| Rap1a | 0,208880687 | 0,154217212 | P62836 |
| Maob | 0,208823529 | 0,437658511 | P19643 |
| Cc2d1b | 0,208640903 | 0,154701428 | Q5FVK6 |
| Psmb1 | 0,208423795 | 0,155140814 | P18421 |
| Purb | -0,208212338 | 0,155569645 | Q68A21 |
| Tomm40 | 0,207789389 | 0,156429985 | Q75Q40 |
| Nos3 | 0,206583982 | 0,158901092 | Q62600 |
| Gosr2 | 0,206421147 | 0,159237085 | O35165 |
| Dbh | -0,205882353 | 0,444284641 | Q05754 |
| Csnk1d | 0,205882353 | 0,444284641 | Q06486 |
| Uqcc3 | -0,205818498 | 0,160485109 | P0CD94 |
| Anks1b | -0,205499545 | 0,259182789 | P0C6S7 |
| Map1lc3b | 0,205275727 | 0,161615234 | Q62625 |
| Mrps10 | -0,205275727 | 0,161615234 | Q7TQ82 |
| Tpd52l2 | 0,205064184 | 0,162057267 | Q6PCT3 |
| Rbbp7 | 0,20501547 | 0,162159183 | Q71UF4 |
| Rala | 0,204435037 | 0,163377123 | P63322 |
| Fmo3 | -0,204195728 | 0,163881208 | Q9EQ76 |
| Erap1 | -0,204190187 | 0,163892894 | Q9JJ22 |
| Rpl34 | 0,203327272 | 0,16572006 | P11250 |
| Them6 | 0,202583868 | 0,167306015 | Q5XIE1 |
| Flot1 | -0,202258171 | 0,16800431 | Q9Z1E1 |
| Bcam | -0,201916031 | 0,168740133 | Q9ESS6 |
| Psmb3 | 0,201246334 | 0,269386883 | P40112 |
| Prmt1 | 0,201210411 | 0,170265056 | Q63009 |
| Prune1 | -0,200147221 | 0,457350017 | Q6AYG3 |
| Slc12a7 | -0,200081423 | 0,172725651 | Q5RK27 |
| Gorasp2 | 0,2 | 0,457687905 | Q9R064 |
| Ldaf1 | -0,198827553 | 0,175488442 | Q6UK00 |
| Plpp7 | -0,198767878 | 0,17562072 | Q5FVJ3 |
| Araf | 0,197953701 | 0,177432654 | P14056 |
| G6pc3 | 0,197947214 | 0,277479677 | Q6AZ83 |
| Maoa | -0,197845144 | 0,177675258 | P21396 |
| Mt-co1 | -0,197682308 | 0,178039613 | P05503 |
| Psma6 | 0,197633393 | 0,178149167 | P60901 |
| Snrpn | 0,197580645 | 0,278388472 | P63164 |
| Mpc1 | -0,197372708 | 0,178733845 | P63031 |
| Slc9a6 | -0,197058824 | 0,464464211 | D3ZJ86 |
| Psmb8 | 0,197058824 | 0,464464211 | P28064 |
| Ccdc141 | -0,196819194 | 0,179979865 | D3ZEY0 |
| Atp1a3 | -0,196482848 | 0,180740056 | P06687 |
| Psma4 | 0,196010314 | 0,181811946 | P21670 |
| Aarsd1 | 0,19536424 | 0,183284862 | Q5XI97 |
| Psmc5 | 0,195087529 | 0,183918311 | P62198 |
| Stat5b | 0,195032537 | 0,284758787 | P52632 |
| Cdk5 | 0,194117647 | 0,471289728 | Q03114 |
| Glrx2 | -0,194094659 | 0,186204106 | Q6AXW1 |
| Clic4 | 0,1940177 | 0,186382127 | Q9Z0W7 |
| Pes1 | -0,193443335 | 0,187714575 | Q3B8N8 |
| Mmgt1 | 0,19313864 | 0,188424179 | B5DF51 |
| Emc10 | 0,192975791 | 0,188804221 | Q6AYH6 |
| Ppt1 | 0,192832922 | 0,290332505 | P45479 |
| Clip1 | 0,192788867 | 0,474389415 | Q9JK25 |
| Lyrm1 | -0,191715543 | 0,293190386 | B2RYU8 |
| Psma1 | 0,191277445 | 0,192800278 | P18420 |
| Cacfd1 | 0,191176471 | 0,478164013 | D4A9I3 |
| Naa11 | -0,191176471 | 0,478164013 | Q4V8K3 |
| Atp5pf | -0,191065516 | 0,193303112 | P21571 |
| Ywhag | -0,190799295 | 0,193936083 | P61983 |
| Mark2 | 0,190517546 | 0,194607575 | O08679 |
| Ppp2r2a | 0,189555967 | 0,196911717 | P36876 |
| Ndufv3 | -0,189318281 | 0,197484227 | Q6PCU8 |
| Cst3 | 0,189274277 | 0,197590347 | P14841 |
| Top2a | 0,18914956 | 0,299820874 | P41516 |
| Mob1a | 0,188235294 | 0,485086609 | Q3T1J9 |
| Plbd2 | -0,188235294 | 0,485086609 | Q4QQW8 |
| Gps1 | -0,187907078 | 0,200907629 | P97834 |
| Coro1b | 0,187517185 | 0,304087905 | O89046 |
| Myzap | -0,187216719 | 0,202597515 | Q5EB94 |
| Tubb2a | -0,186821537 | 0,203569352 | P85108 |
| Slc2a3 | 0,186583578 | 0,306545473 | Q07647 |
| Jph2 | -0,186229544 | 0,205031321 | Q2PS20 |
| Xpnpep3 | -0,18601243 | 0,205569344 | B5DEQ3 |
| Ppm1a | -0,185539031 | 0,206745898 | P20650 |
| Alad | -0,185410334 | 0,207066566 | P06214 |
| As3mt | -0,185294118 | 0,49205705 | Q8VHT6 |
| Micu3 | 0,185203279 | 0,207583212 | A0A8I6A2H6 |
| Csnk2a1 | -0,185193226 | 0,207608318 | P19139 |
| Dpysl3 | -0,185035417 | 0,208002725 | Q62952 |
| Rtcb | 0,184986159 | 0,208125941 | Q6AYT3 |
| Snapin | -0,184967922 | 0,310827834 | P60192 |
| Gk | 0,184438353 | 0,209499695 | Q63060 |
| Sub1 | 0,183890578 | 0,21087971 | Q63396 |
| Sdhb | 0,18346632 | 0,211952906 | P21913 |
| Golga4 | -0,183101269 | 0,212879381 | Q5U4E6 |
| Sh3glb1 | 0,182487172 | 0,49875355 | Q6AYE2 |
| Vac14 | 0,182352941 | 0,499074857 | Q80W92 |
| Uba3 | -0,182352941 | 0,499074857 | Q99MI7 |
| St3gal6 | 0,182352941 | 0,499074857 | P61943 |
| Ufl1 | 0,182352941 | 0,499074857 | B2GV24 |
| Hk1 | -0,18210932 | 0,215411153 | P05708 |
| Pank4 | -0,181851515 | 0,319193233 | Q923S8 |
| Cdk5rap3 | 0,181818182 | 0,319283459 | Q9JLH7 |
| Vdac1 | 0,181684943 | 0,21650068 | Q9Z2L0 |
| Pura | -0,18167015 | 0,216538729 | P86252 |
| Pkp2 | -0,181581306 | 0,216767334 | F1M7L9 |
| Hint1 | -0,18145796 | 0,217084997 | P62959 |
| Aifm1 | -0,181240841 | 0,217644949 | Q9JM53 |
| Cox7a2 | -0,18032787 | 0,220010489 | P35171 |
| Psmd1 | 0,180091185 | 0,220626647 | O88761 |
| Stoml2 | -0,180007059 | 0,220845941 | Q4FZT0 |
| Scn7a | -0,179985337 | 0,324268909 | F1LQQ7 |
| Ucp3 | -0,179411765 | 0,50613954 | P56499 |
| Akr1e2 | -0,179411765 | 0,50613954 | Q5U1Y4 |
| Fgfr1op2 | -0,179411765 | 0,50613954 | Q6TA25 |
| Acot8 | -0,179252199 | 0,326276481 | Q8VHK0 |
| Chmp4c | -0,178961082 | 0,223585127 | Q569C1 |
| Arfgef1 | 0,178519062 | 0,328291701 | D4A631 |
| Acp2 | 0,178152493 | 0,329302177 | P20611 |
| Ca1 | -0,177490705 | 0,227475314 | B0BNN3 |
| Xk | 0,177485888 | 0,227488135 | Q5GH61 |
| Atad1 | 0,177386962 | 0,227751538 | Q505J9 |
| Abcc8 | -0,176676527 | 0,22964934 | Q09429 |
| Anxa8 | 0,176470588 | 0,513250597 | Q4FZU6 |
| Kif5b | 0,175921403 | 0,231678418 | Q2PQA9 |
| Msra | -0,175808071 | 0,231984008 | Q923M1 |
| Trip10 | -0,175785905 | 0,335871809 | P97531 |
| Psma3 | 0,175102451 | 0,233892883 | P18422 |
| Lman1 | 0,174831058 | 0,234629923 | Q62902 |
| Eif5b | -0,174446374 | 0,23567736 | B2GUV7 |
| Acp1 | -0,17379505 | 0,237458104 | P41498 |
| Sdhd | -0,173750204 | 0,237581052 | Q6PCT8 |
| P3h1 | 0,173529412 | 0,520407514 | Q9R1J8 |
| Prmt3 | 0,173529412 | 0,520407514 | O70467 |
| Stx2 | 0,173529412 | 0,520407514 | P50279 |
| Cox14 | -0,173103542 | 0,239358745 | Q5XFV8 |
| Ywhae | -0,172659918 | 0,240583515 | P62260 |
| Tomm70 | 0,171958965 | 0,242527416 | Q75Q39 |
| Krt75 | -0,171920821 | 0,346771984 | Q6IG05 |
| Mt-co3 | -0,171791462 | 0,242993514 | P05505 |
| Ptpmt1 | -0,17114012 | 0,244811746 | P0C089 |
| Aoc3 | -0,171040847 | 0,245089676 | O08590 |
| Ahcyl1 | 0,170755536 | 0,245889642 | B5DFN2 |
| Srp54 | 0,17065347 | 0,350392108 | Q6AYB5 |
| Mgll | -0,169891714 | 0,248322442 | Q8R431 |
| Vps29 | 0,169887104 | 0,248335471 | B2RZ78 |
| Pgam1 | -0,169846655 | 0,248449788 | P25113 |
| Fscn1 | 0,169787766 | 0,24861629 | P85845 |
| Ero1a | -0,169783157 | 0,248629322 | Q8R4A1 |
| Arglu1 | -0,169566043 | 0,249243849 | Q5BJT0 |
| Psmb4 | 0,168643309 | 0,251867037 | P34067 |
| Bst2 | 0,167716023 | 0,254521855 | Q811A2 |
| Itpr1 | -0,167647059 | 0,534856825 | P29994 |
| Camk1 | -0,167647059 | 0,534856825 | Q63450 |
| Cpq | -0,167612017 | 0,254820791 | Q6IRK9 |
| Acaa1a | -0,167286346 | 0,255758375 | P21775 |
| Ppp2ca | -0,166811422 | 0,257129799 | P63331 |
| Actc1 | -0,166648582 | 0,25760116 | P68035 |
| Slc16a1 | 0,165666984 | 0,2604548 | P53987 |
| Exoc8 | 0,164827123 | 0,541846706 | O54924 |
| Rab4b | 0,164705882 | 0,542148135 | P51146 |
| Snx20 | -0,164705882 | 0,542148135 | Q5BK61 |
| Cisd1 | 0,164698859 | 0,263289911 | B0K020 |
| Stxbp1 | 0,164631168 | 0,263488908 | P61765 |
| Leo1 | -0,164604528 | 0,367981964 | Q641X2 |
| Dynll2 | -0,164518143 | 0,263821402 | Q78P75 |
| Cyp27a1 | -0,164459401 | 0,263994317 | P17178 |
| Ap3m1 | 0,164222874 | 0,369108988 | P53676 |
| Uqcrq | -0,163595408 | 0,266546339 | Q7TQ16 |
| Septin8 | -0,163489736 | 0,371279647 | B0BNF1 |
| Arhgdia | 0,163378294 | 0,267190213 | Q5XI73 |
| Pcyt2 | -0,163052623 | 0,26815796 | O88637 |
| Palm | -0,162514249 | 0,269762865 | Q920Q0 |
| Atpsckmt | -0,162071211 | 0,271088338 | D3ZLY0 |
| Tp53i11 | 0,161883782 | 0,549185338 | B3DMA0 |
| Gulp1 | 0,161764706 | 0,549483143 | Q5PQS4 |
| Polr2e | 0,161764706 | 0,549483143 | B0BNE2 |
| Cul7 | 0,161764706 | 0,549483143 | D3ZEF4 |
| Dcun1d5 | 0,161764706 | 0,549483143 | Q5PPL2 |
| Strn3 | -0,161121804 | 0,37834174 | P58405 |
| Pdia6 | 0,16072301 | 0,275148376 | Q63081 |
| Myh7 | -0,16066873 | 0,275312674 | P02564 |
| Gsk3a | -0,160557185 | 0,380037167 | P18265 |
| Pacs1 | -0,160334347 | 0,276326227 | O88588 |
| Enpep | -0,160225792 | 0,276655796 | P50123 |
| Mcts1 | -0,160021997 | 0,3816483 | Q4G009 |
| Tuba8 | -0,159850191 | 0,277798112 | Q6AY56 |
| Corin | -0,159741634 | 0,278128845 | Q80YN4 |
| Rhob | 0,158823529 | 0,556861281 | P62747 |
| Cd81 | -0,158710343 | 0,281283726 | Q62745 |
| Cyb5r1 | -0,158009011 | 0,283442573 | Q5EB81 |
| Arrb1 | 0,157991202 | 0,387797895 | P29066 |
| Tiprl | -0,157946157 | 0,283636577 | A2VCX1 |
| Dcakd | 0,157266166 | 0,285741008 | Q6AY55 |
| Cul5 | -0,157094779 | 0,28627302 | Q9JJ31 |
| Abcb6 | 0,156652011 | 0,287650434 | O70595 |
| Gnl1 | -0,156158358 | 0,393396939 | Q6MG06 |
| Eif2b2 | 0,155882353 | 0,564281969 | Q62818 |
| Mapk10 | -0,155882353 | 0,564281969 | P49187 |
| Hspb3 | -0,155882353 | 0,564281969 | Q9QZ58 |
| Blmh | 0,155123752 | 0,292437889 | P70645 |
| Slc25a12 | -0,154430724 | 0,294625834 | F1LX07 |
| Pgrmc2 | 0,154050754 | 0,295829921 | Q5XIU9 |
| Rpl27 | 0,153821103 | 0,296559203 | P61354 |
| Pde3a | -0,153770999 | 0,296718468 | Q62865 |
| Spr | 0,152941176 | 0,571744619 | P18297 |
| Csnk1g3 | -0,152941176 | 0,571744619 | Q62763 |
| Ndufb1 | -0,152576872 | 0,300530602 | P0DN35 |
| Ppp5c | -0,152196922 | 0,301750142 | P53042 |
| Mt-nd4 | 0,151879496 | 0,302771439 | P05508 |
| Itfg1 | -0,151002795 | 0,30560369 | Q8R4E1 |
| Dhx30 | 0,150659824 | 0,410470373 | Q5BJS0 |
| Xdh | 0,150413897 | 0,307515683 | P22985 |
| Calu | 0,150301254 | 0,307882272 | O35783 |
| Nt5c2 | -0,150246974 | 0,308059024 | D3ZMY7 |
| Golim4 | 0,150021711 | 0,30879324 | Q5BJK8 |
| Crk | 0,15 | 0,579248631 | Q63768 |
| Ace | 0,15 | 0,579248631 | P47820 |
| Bles03 | 0,15 | 0,579248631 | Q566Q8 |
| Flot2 | -0,149541334 | 0,310362697 | Q9Z2S9 |
| Pafah1b1 | -0,148618575 | 0,313391741 | P63004 |
| Sts | 0,148463794 | 0,313901659 | P15589 |
| Mapk1 | 0,14830431 | 0,417910256 | P63086 |
| Anp32a | -0,148234592 | 0,314657725 | P49911 |
| Gpd1 | -0,147975248 | 0,315514613 | O35077 |
| Hexa | -0,147862668 | 0,315887043 | Q641X3 |
| Pyroxd2 | -0,147695815 | 0,316439532 | Q68FT3 |
| Wdr1 | 0,147219283 | 0,318020813 | Q5RKI0 |
| Sgta | 0,147058824 | 0,586793394 | O70593 |
| Mospd1 | 0,146986186 | 0,318796121 | Q5RJS6 |
| Srsf2 | 0,146330873 | 0,320982176 | Q6PDU1 |
| Rcn2 | -0,146091101 | 0,424968822 | Q62703 |
| Pip4k2c | -0,146009173 | 0,322058788 | O88370 |
| Camk2a | -0,14444139 | 0,430272925 | P11275 |
| Egln1 | 0,14444139 | 0,430272925 | P59722 |
| Nsfl1c | -0,144428152 | 0,430315633 | O35987 |
| Rab28 | -0,144428152 | 0,430315633 | P51158 |
| Plscr3 | 0,144117647 | 0,594378288 | Q6QBQ4 |
| Nucb1 | -0,143725575 | 0,329766553 | Q63083 |
| Abcb7 | -0,142969577 | 0,332343491 | Q704E8 |
| Ube2v2 | -0,142372513 | 0,334387539 | Q7M767 |
| Cox7b | -0,142325961 | 0,334547241 | P80431 |
| Cacna1c | 0,14195755 | 0,335812771 | P22002 |
| Prkacb | -0,141833578 | 0,3362393 | P68182 |
| Niban1 | 0,141457457 | 0,337535407 | Q9ESN0 |
| Naa35 | 0,141176471 | 0,602002684 | Q6DKG0 |
| Mapkapk3 | 0,141176471 | 0,602002684 | Q66H84 |
| Sik1 | 0,141176471 | 0,602002684 | Q9R1U5 |
| Cyb5r3 | 0,140906994 | 0,339437886 | P20070 |
| Atp1b1 | 0,140747978 | 0,339988701 | P07340 |
| Vldlr | 0,140705156 | 0,34013713 | P98166 |
| Slc25a10 | -0,140635601 | 0,340378299 | O89035 |
| Gnb3 | -0,140371828 | 0,341293854 | P52287 |
| Eif3e | 0,139600521 | 0,343979796 | Q641X8 |
| Cpt1a | 0,138305379 | 0,348519156 | P32198 |
| Apeh | -0,138189318 | 0,348927729 | P13676 |
| Dnajc16 | 0,138084512 | 0,349296935 | Q5FVM7 |
| Pi4ka | -0,137758841 | 0,350445718 | O08662 |
| Ube2n | -0,1377083 | 0,350624206 | Q9EQX9 |
| Phb2 | 0,137603474 | 0,350994577 | Q5XIH7 |
| Elob | -0,13694838 | 0,353314607 | P62870 |
| Rras | 0,13631552 | 0,355564772 | D3Z8L7 |
| Lum | -0,136235345 | 0,355850462 | P51886 |
| Scamp1 | 0,134990637 | 0,360303691 | P56603 |
| Rnf123 | -0,134607034 | 0,361682906 | D3ZXK7 |
| Micu2 | -0,13449848 | 0,362073784 | Q99P63 |
| Mrs2 | 0,133633674 | 0,365196883 | Q9ET09 |
| Nmt1 | 0,133420181 | 0,365970373 | Q8K1Q0 |
| Nln | -0,133087277 | 0,367178462 | P42676 |
| Cd200 | 0,133087277 | 0,367178462 | P04218 |
| Dcxr | -0,132870169 | 0,367967628 | Q920P0 |
| Mlip | -0,132352941 | 0,625106429 | A0A096MK47 |
| Stk3 | 0,132352941 | 0,625106429 | O54748 |
| Pitpnb | 0,132352544 | 0,369853256 | P53812 |
| Gstm5 | 0,132229611 | 0,370301936 | Q9Z1B2 |
| Ubac1 | -0,131964809 | 0,471547613 | Q5XIR9 |
| Tomm22 | -0,131256108 | 0,373866537 | Q75Q41 |
| Cav2 | -0,130648357 | 0,376102262 | Q2IBC5 |
| Krt14 | 0,129577213 | 0,380062038 | Q6IFV1 |
| Add3 | -0,129411765 | 0,632882332 | Q62847 |
| Akap1 | -0,129411765 | 0,632882332 | O88884 |
| Tmem175 | 0,129411765 | 0,632882332 | Q6AY05 |
| Lxn | 0,129411765 | 0,632882332 | Q64361 |
| Ppid | 0,129403463 | 0,380706678 | Q6DGG0 |
| Speg | -0,129237116 | 0,381324463 | Q63638 |
| Exoc4 | 0,129032258 | 0,481540476 | Q62824 |
| Ccdc127 | -0,128049939 | 0,385750675 | Q6PEB9 |
| Cox6a1 | -0,127018592 | 0,389620421 | P10818 |
| Pln | -0,126594649 | 0,391217705 | P61016 |
| Dhodh | 0,126587776 | 0,391243631 | Q63707 |
| Agrn | -0,126470588 | 0,640694441 | P25304 |
| Chrm2 | -0,126470588 | 0,640694441 | P10980 |
| Trappc3 | 0,125962605 | 0,492116457 | Q5U1Z2 |
| Actr1a | 0,125766705 | 0,394348204 | P85515 |
| Aprt | -0,125006785 | 0,397234355 | P36972 |
| Irgm | 0,124732 | 0,398281005 | Q6AYC2 |
| Kpna1 | 0,124633431 | 0,496732297 | P83953 |
| Psme1 | 0,124626825 | 0,398682038 | Q63797 |
| Fam98a | -0,123529412 | 0,648542067 | Q5FWT1 |
| Rad50 | 0,123529412 | 0,648542067 | Q9JIL8 |
| Magi3 | -0,123529412 | 0,648542067 | Q9JK71 |
| Blvra | 0,122899874 | 0,405300489 | P46844 |
| Tra2b | 0,121906209 | 0,409137252 | P62997 |
| Dync1h1 | 0,121695707 | 0,409952725 | P38650 |
| Wnk1 | 0,121529568 | 0,410596992 | Q9JIH7 |
| Tpm2 | -0,121471993 | 0,410820398 | P58775 |
| Fabp4 | -0,121424307 | 0,411005486 | P70623 |
| Cstb | 0,121207187 | 0,411848812 | P01041 |
| Tpp1 | 0,121173238 | 0,508850453 | Q9EQV6 |
| Eif5a | 0,120725221 | 0,413724383 | Q3T1J1 |
| Myo18a | -0,120712115 | 0,413775453 | D3ZFD0 |
| Fyn | -0,120588235 | 0,656424515 | Q62844 |
| Grb14 | 0,120588235 | 0,656424515 | O88900 |
| Crbn | 0,120588235 | 0,656424515 | Q56AP7 |
| Naa25 | -0,120588235 | 0,656424515 | Q6QI44 |
| Atp5f1e | 0,120386452 | 0,415045619 | P29418 |
| Atp5pb | -0,120236674 | 0,415630539 | P19511 |
| Lmod2 | -0,120179129 | 0,415855392 | A1A5Q0 |
| Fxr1 | 0,119741627 | 0,417567164 | Q5XI81 |
| Ttr | 0,119629821 | 0,418005264 | P02767 |
| Myl9 | 0,119300912 | 0,419295563 | Q64122 |
| Lgals3bp | -0,119134897 | 0,516057197 | O70513 |
| Abcc1 | 0,118879601 | 0,420951657 | Q8CG09 |
| Mvk | -0,117647059 | 0,664341079 | P17256 |
| Cdc37l1 | 0,117647059 | 0,664341079 | Q5XIC3 |
| Pelo | 0,117302212 | 0,427184948 | Q5XIP1 |
| Ywhah | -0,116861617 | 0,428935271 | P68511 |
| Npr1 | 0,116698781 | 0,429583176 | P18910 |
| Ank3 | 0,116484829 | 0,430435305 | O70511 |
| Tsc1 | 0,116261989 | 0,668080791 | Q9Z136 |
| Psmc2 | 0,115284412 | 0,435233875 | Q63347 |
| Coq2 | 0,115175858 | 0,435669277 | Q499N4 |
| Slc25a46 | 0,114744755 | 0,437400793 | Q5EB62 |
| Atp1b2 | -0,114705882 | 0,672291047 | P13638 |
| Becn1 | 0,114705882 | 0,672291047 | Q91XJ1 |
| Bin3 | 0,114705882 | 0,672291047 | Q68FW8 |
| Atp5f1c | -0,114645533 | 0,437799859 | P35435 |
| Pfkfb2 | -0,114207151 | 0,533684696 | Q9JJH5 |
| Gypc | -0,112525445 | 0,446374984 | Q6XFR6 |
| Hmgb1 | 0,111810682 | 0,449286685 | P63159 |
| Ctps2 | 0,111803519 | 0,54238639 | Q5U2N0 |
| Mlst8 | 0,111764706 | 0,680273695 | Q9Z2K5 |
| Ppm1g | 0,111267911 | 0,451504686 | F1LNI5 |
| Ddah1 | -0,110942249 | 0,452838356 | O08557 |
| Fahd2a | -0,110788438 | 0,453468998 | B2RYW9 |
| Aars1 | 0,110337243 | 0,547727388 | P50475 |
| Slc25a11 | -0,110254602 | 0,455661501 | P97700 |
| Rtn2 | -0,110134072 | 0,456157325 | Q6WN19 |
| Gna11 | 0,109968247 | 0,456839954 | Q9JID2 |
| Psmb6 | 0,109916952 | 0,457051227 | P28073 |
| Vcp | 0,109865654 | 0,457262564 | P46462 |
| Anxa6 | 0,109434373 | 0,459041452 | P48037 |
| Acsl6 | -0,108336952 | 0,463584799 | P33124 |
| Kank2 | -0,108014221 | 0,464925506 | D3ZD05 |
| Lmf2 | 0,107974337 | 0,556385977 | A1L1J9 |
| Herc4 | 0,107771261 | 0,55713307 | Q5PQN1 |
| Sfxn5 | -0,107745753 | 0,466042379 | Q8CFD0 |
| Eif3a | 0,107251411 | 0,468102686 | Q1JU68 |
| Xpnpep2 | -0,106822993 | 0,469892176 | Q99MA2 |
| Dctn4 | 0,106765815 | 0,470131282 | Q9QUR2 |
| Cndp2 | 0,106711537 | 0,470358324 | Q6Q0N1 |
| Tgm2 | -0,105948762 | 0,473555125 | Q9WVJ6 |
| Ndufs2 | 0,105317447 | 0,476209691 | Q641Y2 |
| Opa1 | -0,105148876 | 0,476919833 | Q2TA68 |
| Fasn | 0,10443184 | 0,479946757 | P12785 |
| Tpp2 | 0,104320452 | 0,480417883 | Q64560 |
| Sirt5 | -0,103617662 | 0,483396 | Q68FX9 |
| Lrrc4b | -0,103354685 | 0,484512869 | P0CC10 |
| Lmna | -0,103243317 | 0,48498626 | P48679 |
| Psma7 | 0,103186235 | 0,485228992 | P48004 |
| Prpsap1 | -0,102941176 | 0,704410382 | Q63468 |
| Celsr2 | -0,102941176 | 0,704410382 | Q9QYP2 |
| Napepld | -0,10265458 | 0,487492809 | Q769K2 |
| Plgrkt | 0,102214744 | 0,489369819 | D4ACN8 |
| Krt5 | -0,101906158 | 0,57890755 | Q6P6Q2 |
| Oat | -0,101720675 | 0,491482744 | P04182 |
| Fam162a | 0,101343466 | 0,493099092 | Q4QQV3 |
| Fmo1 | -0,101173021 | 0,581655788 | P36365 |
| Nrdc | -0,100743636 | 0,495675036 | P47245 |
| Prorp | 0,1 | 0,71251637 | B5DF07 |
| Stx17 | 0,1 | 0,71251637 | Q9Z158 |
| Chm | 0,1 | 0,71251637 | P37727 |
| Prps1 | 0,099983716 | 0,498948423 | P60892 |
| Eif3c | 0,099978289 | 0,49897184 | B5DFC8 |
| Slc25a15 | 0,099495196 | 0,501058593 | A0A0G2K309 |
| Surf1 | -0,099006677 | 0,503173326 | Q9QXU2 |
| Timm21 | -0,098632071 | 0,504798023 | Q5U2X7 |
| Kif1c | 0,098607038 | 0,591319882 | O35787 |
| Krt13 | -0,09860194 | 0,716379672 | Q6IFV4 |
| Scp2 | -0,097484191 | 0,509793074 | P11915 |
| Cd38 | 0,097264438 | 0,510752187 | Q64244 |
| Prkaa2 | -0,096721667 | 0,513125003 | Q09137 |
| Slc25a42 | 0,096564078 | 0,513814973 | P0C546 |
| Rtn1 | -0,096406471 | 0,514505483 | Q64548 |
| Cand1 | 0,096127229 | 0,515730051 | P97536 |
| Ggcx | 0,096049858 | 0,601019855 | O88496 |
| Itga1 | 0,095744681 | 0,517410018 | P18614 |
| Neu3 | -0,095729117 | 0,724338412 | Q99PW5 |
| Arf5 | 0,095429378 | 0,518796732 | P84083 |
| Gnaq | 0,095258773 | 0,519547836 | P82471 |
| Adcy6 | 0,094230039 | 0,524088385 | Q03343 |
| Psmb10 | 0,094117647 | 0,728814427 | Q4KM35 |
| Ace2 | -0,094117647 | 0,728814427 | Q5EGZ1 |
| Dnah12 | -0,094117647 | 0,728814427 | Q923J6 |
| Psmb7 | 0,093356492 | 0,527959362 | Q9JHW0 |
| Prkab1 | -0,093300339 | 0,611524753 | P80386 |
| Akt2 | 0,092382012 | 0,532294162 | P47197 |
| Pkm | -0,09227596 | 0,532766968 | P11980 |
| Mpv17 | -0,0921674 | 0,533251164 | Q5BK62 |
| Nisch | 0,092008798 | 0,616485735 | Q4G017 |
| Syn1 | -0,091842348 | 0,617126307 | P09951 |
| Cdnf | -0,091642229 | 0,617896821 | P0C5I0 |
| Ddx46 | 0,091350721 | 0,536900579 | Q62780 |
| Prkag1 | -0,091242164 | 0,53738659 | P80385 |
| Npm1 | 0,09118541 | 0,53764076 | P13084 |
| Ctbs | 0,091176471 | 0,73700495 | Q01460 |
| Cd36 | -0,091138554 | 0,537850652 | Q07969 |
| Ckmt2 | -0,090482265 | 0,540794639 | P09605 |
| Fam234b | 0,090272501 | 0,541737238 | D3ZWJ9 |
| Sar1b | 0,089722365 | 0,5442131 | Q5HZY2 |
| Ehd3 | -0,089672955 | 0,544435737 | Q8R491 |
| Utrn | -0,089442815 | 0,626391309 | G3V7L1 |
| Rabggtb | 0,089275895 | 0,627037924 | Q08603 |
| Ctsh | -0,088964881 | 0,547631003 | P00786 |
| Myl3 | -0,088799631 | 0,548378001 | P16409 |
| Myh4 | 0,088636795 | 0,549114561 | Q29RW1 |
| Psmb2 | 0,088363005 | 0,550354071 | P40307 |
| Hprt1 | -0,087987841 | 0,552054677 | P27605 |
| Syde1 | 0,087984603 | 0,63204929 | D3ZZN9 |
| Emb | 0,087279833 | 0,555270843 | O88775 |
| Impa1 | -0,087243402 | 0,634933094 | P97697 |
| Hyou1 | 0,087227922 | 0,555507 | Q63617 |
| Eif4e | 0,086628491 | 0,558237392 | P63074 |
| Dpp4 | -0,086475044 | 0,558937354 | P14740 |
| Gnao1 | -0,086470349 | 0,558958774 | P59215 |
| Acbd6 | -0,086031427 | 0,560963257 | Q5RJK8 |
| Caprin1 | 0,085968289 | 0,639906499 | Q5M9G3 |
| Rtn4 | 0,085927535 | 0,56143821 | Q9JK11 |
| Ube2z | -0,085868592 | 0,561707755 | Q3B7D1 |
| Tomm40l | 0,085663103 | 0,562647925 | A4F267 |
| Oas1a | -0,085294118 | 0,753465069 | Q05961 |
| Ndufs4 | 0,084620186 | 0,567430858 | Q5XIF3 |
| Brox | -0,08431085 | 0,64639411 | Q4V8K5 |
| Dipk1b | 0,083050695 | 0,651343823 | Q5FVL3 |
| Atp5pd | 0,082991831 | 0,574936094 | P31399 |
| Pip4k2b | -0,082937553 | 0,575187048 | O88377 |
| Cltb | -0,08266616 | 0,57644257 | P08082 |
| Get1 | -0,082352941 | 0,761733079 | Q6P6S5 |
| Txn2 | -0,082352941 | 0,761733079 | P97615 |
| Folh1 | 0,082352941 | 0,761733079 | P70627 |
| Pafah2 | 0,082127833 | 0,578936681 | P83006 |
| Cacna2d2 | -0,082123375 | 0,578957356 | Q8CFG6 |
| Wars1 | 0,082066869 | 0,579219439 | Q6P7B0 |
| Itm2b | 0,081973889 | 0,579650814 | Q5XIE8 |
| Uqcrfs1 | -0,081205048 | 0,583223376 | P20788 |
| Gbp2 | -0,081202457 | 0,658629851 | Q63663 |
| Hspa5 | 0,081040004 | 0,583991575 | P06761 |
| Ddb1 | -0,080605765 | 0,586014923 | Q9ESW0 |
| Ptdss2 | 0,080336545 | 0,587270933 | B2GV22 |
| Set | -0,080171525 | 0,58804141 | Q63945 |
| Arpc1a | -0,080004342 | 0,588822443 | Q99PD4 |
| Sae1 | 0,079411765 | 0,770025319 | Q6AXQ0 |
| Vps50 | 0,079411765 | 0,770025319 | F1LSG8 |
| Crym | -0,079244464 | 0,592378216 | Q9QYU4 |
| Gars1 | 0,078816665 | 0,594384238 | Q5I0G4 |
| Cap2 | 0,078488846 | 0,595923466 | P52481 |
| Mt-nd2 | 0,078111006 | 0,597699736 | P11662 |
| Capza2 | 0,078108886 | 0,597709709 | Q3T1K5 |
| Tmem245 | 0,078086335 | 0,670983692 | D3ZXD8 |
| Ube4a | 0,078086335 | 0,670983692 | Q6P7A2 |
| Ntmt1 | 0,078050369 | 0,597985013 | Q5BJX0 |
| Sirt2 | -0,077941815 | 0,598495875 | Q5RJQ4 |
| Exoc5 | 0,07771261 | 0,672471086 | P97878 |
| Memo1 | -0,07771261 | 0,672471086 | Q4QQR9 |
| Pdlim5 | 0,077672538 | 0,599763936 | Q62920 |
| Fam234a | 0,077403246 | 0,601033241 | Q5M7W6 |
| Ctbp1 | -0,077399045 | 0,601053053 | Q9Z2F5 |
| Vdac2 | 0,077351066 | 0,601279329 | P81155 |
| Jam3 | 0,076612903 | 0,676854867 | Q68FQ2 |
| Mras | 0,076470588 | 0,778340981 | P97538 |
| P33monox | 0,076470588 | 0,778340981 | Q5U2R6 |
| Cdc27 | 0,076470588 | 0,778340981 | Q4V8A2 |
| Ephx2 | -0,076313504 | 0,606181663 | P80299 |
| Myl1 | -0,076246334 | 0,678318448 | P02600 |
| Tsg101 | 0,076104658 | 0,607170518 | Q6IRE4 |
| Gstt2 | 0,074603611 | 0,684891351 | P30713 |
| Ybx1 | 0,074200727 | 0,616217251 | P62961 |
| Pisd | 0,073708207 | 0,618566797 | D3ZAW2 |
| Elac2 | 0,073529412 | 0,786679249 | Q8CGS5 |
| Oplah | -0,072600608 | 0,692936517 | P97608 |
| Cpox | -0,072576268 | 0,623980935 | Q3B7D0 |
| Eif2b3 | -0,072570359 | 0,624009252 | P70541 |
| Cmpk1 | 0,072411454 | 0,624770903 | Q4KM73 |
| Myh6 | -0,072409488 | 0,624780326 | P02563 |
| Pdcd6ip | 0,072079896 | 0,626361347 | Q9QZA2 |
| Ptgfrn | -0,071647624 | 0,62843743 | Q62786 |
| Bves | 0,071271543 | 0,630245958 | Q3BCU4 |
| Gapdh | -0,071215329 | 0,630516471 | P04797 |
| Parm1 | 0,070994355 | 0,631580296 | Q6P9X9 |
| Pgls | -0,070730649 | 0,632850815 | P85971 |
| Abcb8 | 0,070672529 | 0,633130972 | Q5RKI8 |
| Gpcpd1 | -0,070588235 | 0,795039302 | Q80VJ4 |
| Ctbp2 | 0,070588235 | 0,795039302 | Q9EQH5 |
| Tmem186 | -0,070573547 | 0,633608217 | Q4KLZ1 |
| Esd | 0,070560139 | 0,633672876 | B0BNE5 |
| Ppp1ca | 0,069837779 | 0,704087547 | P62138 |
| Ola1 | -0,069761131 | 0,637530869 | A0JPJ7 |
| Ndufa10 | -0,069480255 | 0,638889337 | Q561S0 |
| Enpp1 | 0,068914956 | 0,707825771 | Q924C3 |
| Eif3j | -0,068774597 | 0,642307453 | A0JPM9 |
| Apoa4 | 0,06866417 | 0,642843011 | P02651 |
| Psmb5 | 0,068662306 | 0,64285205 | P28075 |
| Hexb | 0,068557471 | 0,643360661 | Q6AXR4 |
| Atp5mk | 0,068287917 | 0,644669144 | Q9JJW3 |
| Itgad | -0,068280504 | 0,644705146 | Q9QYE7 |
| Smyd2 | 0,067647059 | 0,803420314 | Q7M6Z3 |
| Dnase1 | 0,067647059 | 0,803420314 | P21704 |
| Tsc2 | 0,066904959 | 0,715991049 | P49816 |
| Bsg | -0,066767995 | 0,652067076 | P26453 |
| Pcyt1a | -0,066348974 | 0,718255143 | P19836 |
| Farsa | 0,066348974 | 0,718255143 | Q505J8 |
| Eloc | -0,065950171 | 0,65606148 | P83941 |
| Klc1 | 0,065892314 | 0,656344425 | P37285 |
| Hmgn2 | 0,065733051 | 0,657123539 | P18437 |
| Nedd4 | 0,065678771 | 0,657389159 | Q62940 |
| Fth1 | 0,064915328 | 0,661129496 | P19132 |
| Atp2b2 | -0,064705882 | 0,811821451 | P11506 |
| Cox4i1 | -0,064265748 | 0,664318443 | P10888 |
| Tkfc | -0,063055632 | 0,731713961 | Q4KLZ6 |
| Hdgfl2 | 0,063049853 | 0,731737647 | Q925G1 |
| Nqo1 | -0,062963064 | 0,670731328 | P05982 |
| Rab4a | 0,062854506 | 0,671266791 | P05714 |
| Mapre2 | 0,062691671 | 0,672070288 | Q3B8Q0 |
| Rab1b | 0,062644194 | 0,67230463 | P10536 |
| Pygb | -0,06253223 | 0,672857388 | P53534 |
| Micu1 | 0,062155634 | 0,674717872 | Q6P6Q9 |
| Edf1 | -0,061877493 | 0,676093197 | P69736 |
| Syngr2 | 0,061772523 | 0,736979144 | O54980 |
| Map1a | 0,061764706 | 0,820241878 | P34926 |
| Cox4i2 | 0,061764706 | 0,820241878 | Q91Y94 |
| Gnas | -0,060958068 | 0,680646912 | P63095 |
| Mfn1 | -0,060359334 | 0,683618389 | Q8R4Z9 |
| Timm22 | 0,060142214 | 0,684697115 | Q9JKW1 |
| Limk1 | -0,060035283 | 0,685228611 | P53669 |
| Krt10 | -0,0588363 | 0,691198397 | Q6IFW6 |
| Mapk3 | -0,058823529 | 0,82868075 | P21708 |
| Sumo3 | 0,058675062 | 0,692002639 | Q5XIF4 |
| Fis1 | 0,058516991 | 0,692791407 | P84817 |
| Wbp2 | 0,058284457 | 0,751350989 | Q8R478 |
| Hras | 0,057814452 | 0,696300938 | P20171 |
| Cav3 | -0,05775703 | 0,696588067 | P51638 |
| Fgf13 | 0,057209542 | 0,699327809 | Q9ERW3 |
| Sorbs2 | 0,057006691 | 0,756636598 | O35413 |
| Cacnb2 | -0,056722575 | 0,70176788 | Q8VGC3 |
| Fcgrt | 0,056665219 | 0,702055472 | P13599 |
| Tfb1m | -0,055882353 | 0,837137222 | Q811P6 |
| Arfgef2 | 0,055799816 | 0,706399744 | Q7TSU1 |
| Slc25a51 | 0,055692767 | 0,706937767 | Q52KK3 |
| Alpl | 0,055145462 | 0,709690717 | P08289 |
| Samm50 | 0,054825752 | 0,711300563 | Q6AXV4 |
| Eif3h | 0,054602692 | 0,712424482 | Q6P9U8 |
| Prdx1 | -0,054007111 | 0,715428357 | Q63716 |
| Ralgapa1 | -0,052941176 | 0,845610441 | O55007 |
| Scn1b | 0,052941176 | 0,845610441 | Q00954 |
| Tfrc | 0,052922977 | 0,720907217 | Q99376 |
| Prxl2a | 0,052757273 | 0,72174586 | Q6AXX6 |
| Gpam | -0,052598725 | 0,722548592 | P97564 |
| Parl | 0,052438727 | 0,723358961 | Q3B8P0 |
| Arhgef37 | -0,052057557 | 0,777209343 | A1IGU3 |
| Nherf2 | -0,051243079 | 0,729424216 | Q920G2 |
| Dhrs7c | -0,051021793 | 0,730548563 | D3ZGP9 |
| Trmt10c | -0,050643218 | 0,732473385 | Q5U2R4 |
| Cyb5a | -0,050593057 | 0,732728542 | P00173 |
| Krt2 | -0,050586192 | 0,732763468 | Q6IG02 |
| Septin2 | 0,050480378 | 0,733301828 | Q91Y81 |
| Usp15 | -0,049504682 | 0,738271912 | Q9R085 |
| Obsl1 | 0,049394778 | 0,738832416 | D3ZZ80 |
| Casq1 | 0,048906259 | 0,741325469 | P19633 |
| Hsd17b4 | 0,048093364 | 0,745479709 | P97852 |
| Mybpc3 | -0,047984801 | 0,746035058 | P56741 |
| P4hb | 0,047927918 | 0,746326092 | P04785 |
| Ddx1 | 0,047880137 | 0,746570582 | Q641Y8 |
| Lamtor3 | 0,047546678 | 0,748277549 | Q5U204 |
| Lrrc57 | -0,047498847 | 0,748522494 | Q5FVI3 |
| Dnajc10 | 0,047276576 | 0,749661069 | Q498R3 |
| Gstm1 | 0,047058824 | 0,862603695 | P04905 |
| Ptpre | -0,047058824 | 0,862603695 | B2GV87 |
| Phaf1 | 0,046920821 | 0,798719686 | O08654 |
| Pcsk6 | -0,046842348 | 0,751886909 | Q63415 |
| Cds2 | 0,04679315 | 0,752139224 | Q91XU8 |
| Sbds | -0,046554252 | 0,800260471 | Q5RK30 |
| Cox7c | 0,04564946 | 0,758011846 | P80432 |
| Atad3 | 0,04559518 | 0,758290902 | Q3KRE0 |
| Ndufa5 | 0,045484151 | 0,758861801 | Q63362 |
| Cryl1 | -0,044834043 | 0,762207132 | Q811X6 |
| Atp5mpl | -0,044727914 | 0,762753662 | D3Z9R8 |
| Mcfd2 | -0,044117647 | 0,871122005 | Q8K5B3 |
| Nek7 | 0,043966781 | 0,766676589 | D3ZBE5 |
| Tmem38a | 0,043589187 | 0,768624883 | A6ZIQ8 |
| Serpinh1 | -0,043370878 | 0,769751952 | P29457 |
| Slc25a19 | 0,043264664 | 0,770300477 | Q6AYL0 |
| Arl3 | 0,043255132 | 0,814160081 | P37996 |
| Xpnpep1 | 0,042234407 | 0,77562679 | O54975 |
| Gtf2i | -0,042155425 | 0,818805874 | Q5U2Y1 |
| Septin7 | 0,042011561 | 0,776780229 | Q9WVC0 |
| Gsk3b | -0,041901867 | 0,777348177 | P18266 |
| Cadm3 | -0,041792686 | 0,820339624 | Q1WIM3 |
| Coro6 | 0,041788856 | 0,820355822 | Q920J3 |
| Rhot2 | -0,041632742 | 0,778742075 | Q7TSA0 |
| Oma1 | -0,041471027 | 0,779579984 | D3ZS74 |
| Babam2 | -0,041237158 | 0,879477447 | Q6P7Q1 |
| Hspbp1 | -0,041176471 | 0,879653615 | Q6IMX7 |
| Slc6a17 | -0,041176471 | 0,879653615 | P31662 |
| Atp5me | -0,041036776 | 0,781831244 | P29419 |
| Gstp1 | -0,040655702 | 0,783808275 | P04906 |
| Dgkz | 0,039947894 | 0,787483998 | O08560 |
| Vps35l | 0,03983934 | 0,788048138 | Q5XI83 |
| Mpc2 | 0,039623307 | 0,789171149 | P38718 |
| Pomgnt2 | -0,039603963 | 0,829607743 | Q5NDF0 |
| Fgf1 | -0,038538783 | 0,79481523 | P61149 |
| Ndufv2 | 0,038428137 | 0,795391643 | P19234 |
| Cnn3 | -0,038235294 | 0,888197654 | P37397 |
| Hacl1 | -0,03772356 | 0,799064681 | Q8CHM7 |
| Slc9a9 | 0,037559705 | 0,799919494 | D4A7H1 |
| Rnpep | -0,037289332 | 0,801330507 | O09175 |
| Plaa | -0,036791769 | 0,892395339 | P54319 |
| Gmps | -0,035923754 | 0,845241777 | Q4V7C6 |
| Atp1a2 | 0,03587809 | 0,808705517 | P06686 |
| Ubr4 | -0,035824784 | 0,808984417 | Q2TL32 |
| Cpne9 | 0,035294118 | 0,896753248 | Q5BJS7 |
| Cyp4f1 | 0,035294118 | 0,896753248 | P33274 |
| Pir | -0,035294118 | 0,896753248 | Q5M827 |
| Hspb8 | 0,035294118 | 0,896753248 | Q9EPX0 |
| Tmem126b | 0,033913841 | 0,853805039 | B2RZD2 |
| Pam | -0,033869786 | 0,81922897 | P14925 |
| Hbs1l | -0,033760313 | 0,81980353 | Q6AXM7 |
| Alas1 | -0,033707865 | 0,820078828 | P13195 |
| Nfia | -0,033707865 | 0,820078828 | P09414 |
| Mylk3 | 0,033438281 | 0,821494226 | E9PT87 |
| Mt-co2 | -0,033165966 | 0,822924529 | P00406 |
| Ist1 | 0,033111684 | 0,823209705 | Q568Z6 |
| Epb41l1 | 0,033056506 | 0,823499617 | Q9WTP0 |
| Uqcrc2 | 0,032404266 | 0,826928277 | P32551 |
| Krt1 | -0,032349988 | 0,827213751 | Q6IMF3 |
| Stau2 | -0,03207772 | 0,8616422 | Q68SB1 |
| Arhgef1 | 0,03207772 | 0,8616422 | Q9Z1I6 |
| Nsf | 0,031970038 | 0,829212681 | Q9QUL6 |
| Mt-nd5 | 0,031536666 | 0,831493975 | P11661 |
| Ppp1r7 | 0,031482386 | 0,831779805 | Q5HZV9 |
| Psma5 | 0,031263569 | 0,832932279 | P34064 |
| Dpp7 | -0,03099639 | 0,834339938 | Q9EPB1 |
| Otub1 | 0,030777582 | 0,835493135 | B2RYG6 |
| Hint3 | -0,030395962 | 0,837505224 | Q8K3P7 |
| Sdha | -0,02974462 | 0,840941788 | Q920L2 |
| Acta1 | 0,02941815 | 0,842665389 | P68136 |
| Ikbkb | -0,029411765 | 0,913895587 | Q9QY78 |
| Fam131b | -0,029411765 | 0,913895587 | Q568Z1 |
| Dbx1 | 0,029411765 | 0,913895587 | Q5NSW5 |
| Jmjd6 | -0,029149138 | 0,844086191 | Q6AYK2 |
| Chchd6 | -0,029148347 | 0,84409037 | D4A7N1 |
| Nt5e | 0,029093278 | 0,844381284 | P21588 |
| Slc25a21 | 0,029092488 | 0,844385456 | Q99JD3 |
| Ccdc90b | 0,028822667 | 0,845811125 | Q4V897 |
| Tlr4 | 0,028766826 | 0,84610624 | Q9QX05 |
| Me1 | -0,028225806 | 0,878124632 | P13697 |
| Hdgfl3 | 0,028171308 | 0,849254762 | Q923W4 |
| Lgals1 | 0,027953429 | 0,850407276 | P11762 |
| Skic8 | -0,027859238 | 0,879695915 | Q4V7A0 |
| Ppib | -0,027412132 | 0,853271913 | P24368 |
| Aplp2 | -0,027139252 | 0,854716755 | P15943 |
| Itm2c | 0,026869317 | 0,85614646 | Q5PQL7 |
| Acaca | 0,026761983 | 0,884401918 | P11497 |
| Lipa | -0,026759531 | 0,884412442 | Q64194 |
| Cpe | -0,026470588 | 0,922480571 | P15087 |
| Arl2 | -0,026470588 | 0,922480571 | O08697 |
| Abcb1 | 0,026470588 | 0,922480571 | P43245 |
| Thy1 | 0,025891549 | 0,861329002 | P01830 |
| Iah1 | 0,025847846 | 0,888325516 | Q711G3 |
| Krt6a | 0,025836567 | 0,861620596 | Q4FZU2 |
| Capn1 | 0,025510204 | 0,863351849 | P97571 |
| Tuba4a | 0,025405098 | 0,863909541 | Q5XIF6 |
| Ppp3r1 | 0,025240189 | 0,864784673 | P63100 |
| Ndufa9 | 0,025239504 | 0,864788309 | Q5BK63 |
| Anp32b | -0,024641772 | 0,867961675 | Q9EST6 |
| Gda | 0,024372371 | 0,869392602 | Q9WTT6 |
| Hnrnpc | 0,024318749 | 0,869677463 | G3V9R8 |
| Dnaja2 | 0,024263149 | 0,869972852 | O35824 |
| Chmp3 | -0,024195766 | 0,895422997 | Q8CGS4 |
| Cox6c2 | -0,024155245 | 0,870546171 | P11951 |
| Cdv3 | -0,023529412 | 0,931073584 | Q5XIM5 |
| Phyhip | 0,023529412 | 0,931073584 | Q568Z9 |
| Cyb5b | 0,022961053 | 0,8768955 | P04166 |
| Scn5a | -0,022362136 | 0,880082757 | P15389 |
| Akr1a1 | 0,022362136 | 0,880082757 | P51635 |
| Septin11 | 0,021063489 | 0,887000162 | B3GNI6 |
| Etfdh | -0,021060059 | 0,887018444 | Q6UPE1 |
| Elp1 | 0,02084238 | 0,888178775 | Q8VHU4 |
| Podxl | 0,020734951 | 0,888751504 | Q9WTQ2 |
| Aipl1 | -0,020527859 | 0,91120829 | Q9JLG9 |
| Sdcbp | -0,020355544 | 0,890774665 | Q9JI92 |
| Lrrfip1 | -0,019428152 | 0,915947777 | Q66HF9 |
| Adk | -0,01910707 | 0,897436846 | Q64640 |
| Ppp2r1b | -0,019061584 | 0,917528244 | Q4QQT4 |
| Atp5mf | -0,017967647 | 0,903523224 | D3ZAF6 |
| Lcmt1 | -0,017647059 | 0,94828015 | Q6P4Z6 |
| Tmem177 | -0,016881073 | 0,909332424 | Q4KM93 |
| Ppp2r5b | -0,016863716 | 0,927010666 | Q80W83 |
| Uqcrc1 | -0,016826336 | 0,909625192 | Q68FY0 |
| Txndc12 | -0,016608771 | 0,910788988 | Q498E0 |
| Cyp4b1 | -0,016446386 | 0,911657736 | P15129 |
| Glrx3 | 0,016175872 | 0,913105197 | Q9JLZ1 |
| Atp1a1 | 0,015796331 | 0,915136505 | P06685 |
| Immt | -0,015633482 | 0,916008238 | Q3KR86 |
| Hmga1 | 0,015631785 | 0,916017324 | Q8K585 |
| Csnk2b | 0,015523652 | 0,916596216 | P67874 |
| Ilk | 0,015414677 | 0,917179661 | Q99J82 |
| Trmu | -0,015214004 | 0,934134677 | B1WC37 |
| Tbcel | -0,015030703 | 0,934926552 | Q5PQJ7 |
| Mtus1 | -0,014716707 | 0,956860219 | Q6IMY1 |
| Gmppa | 0,014705882 | 0,956891924 | Q5XIC1 |
| Iqsec1 | -0,014705882 | 0,956891924 | A0A0G2JUG7 |
| Atic | 0,013623904 | 0,926773277 | O35567 |
| Vars1 | 0,013461069 | 0,927646159 | Q04462 |
| Slc25a40 | 0,013354687 | 0,928216467 | Q498U3 |
| Pex2 | 0,013244314 | 0,928808205 | P24392 |
| Cpt1b | 0,012865053 | 0,930841821 | Q63704 |
| Slc25a30 | -0,012592954 | 0,932301094 | Q5PQM9 |
| Myh3 | 0,011764706 | 0,96550817 | P12847 |
| Fam210a | 0,011723838 | 0,936963644 | Q5XIJ4 |
| Cend1 | 0,011507043 | 0,938127021 | Q5FVI4 |
| Vapb | -0,011367804 | 0,950762389 | Q9Z269 |
| Kcnj11 | -0,010747144 | 0,94220581 | P70673 |
| Cep104 | -0,010367475 | 0,94424425 | D3Z8X7 |
| Capns1 | -0,010096076 | 0,945701615 | Q64537 |
| Dnm1l | 0,009336156 | 0,949783155 | O35303 |
| Krt17 | -0,008823529 | 0,974127995 | Q6IFU8 |
| Cdk5rap1 | 0,008823529 | 0,974127995 | Q9JLH6 |
| Rxrg | -0,008823529 | 0,974127995 | Q5BJR8 |
| Phb1 | 0,008630516 | 0,9535743 | P67779 |
| Camk2g | -0,008615159 | 0,962675403 | P11730 |
| Psma2 | 0,008576003 | 0,95386722 | P17220 |
| Prkca | -0,008413168 | 0,954742241 | P05696 |
| Mfn2 | -0,008305738 | 0,955319561 | Q8R500 |
| Pdlim1 | 0,008250109 | 0,955618518 | P52944 |
| Hspb2 | -0,00797894 | 0,957075886 | O35878 |
| Fdps | 0,007697947 | 0,966646833 | P05369 |
| Add1 | 0,007436155 | 0,95999344 | Q63028 |
| Usp1 | -0,007358354 | 0,978423102 | Q569C3 |
| Fastkd2 | -0,007002117 | 0,962326836 | Q5M7V7 |
| Tmem126a | 0,006568055 | 0,964660671 | Q5HZA9 |
| Insrr | -0,006096824 | 0,982121677 | Q64716 |
| Wbp4 | 0,005882353 | 0,982750504 | Q5HZF2 |
| Arhgap29 | 0,005882353 | 0,982750504 | Q5PQJ5 |
| Rrad | 0,005498534 | 0,976173083 | P55043 |
| Prep | 0,005482278 | 0,970499853 | O70196 |
| Psme2 | -0,005428734 | 0,970787844 | Q63798 |
| Decr2 | 0,004667951 | 0,974880223 | Q9Z2M4 |
| Tubb5 | -0,004559518 | 0,975463558 | P69897 |
| Gmpr | 0,004342634 | 0,97663036 | Q9Z244 |
| Tor3a | 0,003665689 | 0,984114143 | Q5M936 |
| Rab12 | 0,003636758 | 0,980428181 | P35284 |
| Rbp1 | -0,003419546 | 0,98159694 | P02696 |
| Mast1 | 0,003311168 | 0,982180102 | Q810W7 |
| Wdr7 | 0,002941176 | 0,991374805 | Q9ERH3 |
| Cops8 | -0,002941176 | 0,991374805 | Q6P4Z9 |
| Akap6 | 0,002941176 | 0,991374805 | Q9WVC7 |
| Poglut1 | -0,002941176 | 0,991374805 | G3V9D0 |
| Cma1 | -0,002876917 | 0,984516832 | P50339 |
| Gtpbp1 | -0,002565982 | 0,988879545 | D2XV59 |
| Mt-atp8 | 0,002225479 | 0,988022488 | P11608 |
| Des | -0,002062527 | 0,988899437 | P48675 |
| Mat2b | 0,001832845 | 0,992056698 | Q5U2R0 |
| Aqp1 | 0,001302649 | 0,992988987 | P29975 |
| Tinagl1 | 0,001194159 | 0,993572876 | Q9EQT5 |
| Slc25a3 | 0,000868503 | 0,995325578 | P16036 |
| Oxsr1 | -0,00043424 | 0,997662843 | A0A8I5ZNK2 |
| Gsr | 0,000271393 | 0,998539315 | P70619 |
| Manba | 0 | 1 | Q4FZV0 |
| Pafah1b2 | 0 | 1 | O35264 |
| Aimp2 | 0 | 1 | Q32PX2 |
