# Supplementary material for: Galectin-3 associates with NF-κB activation and mitochondrial redox–related protein signatures in catecholamine-induced stress cardiomyopathy
Source: Mol Med. 2026 Apr 10;32:61. doi: 10.1186/s10020-026-01472-x (PMC13088642; doi:10.1186/s10020-026-01472-x)

**Galectin-3 associates with NF-κB activation and mitochondrial redox–related protein signatures in catecholamine-induced stress cardiomyopathy**

| **Supplementary materials** | **Page** |
| --- | --- |
| Figure S1. EV isolation in cardiac tissue following catecholamine-induced stress in rats | 2 |
| Figure S2. Nanoparticle tracking analysis (NTA) characterization of EVs | 3 |
| **Figure S3.** Figure S3. Transmission electron microscopy (TEM) characterization of apex-derived EVs collected after 24 hours induced stress | 5 |
| Figure S4. Galectin-3 expression in heart tissue after catecholamine-induced stress in rats | 6 |
| Table S1. PCR primers and corresponding gene accession numbers (Rattus norvegicus) | 7 |
| Table S2. Top 30 EV enriched apical myocardial proteins | 8 |
| Uncropped and Cropped Western blot bands | 9 |

**Figure S1. EV isolation in cardiac tissue following catecholamine-induced stress in rats**

Schematic experimental design for extracellular vesicle isolation.


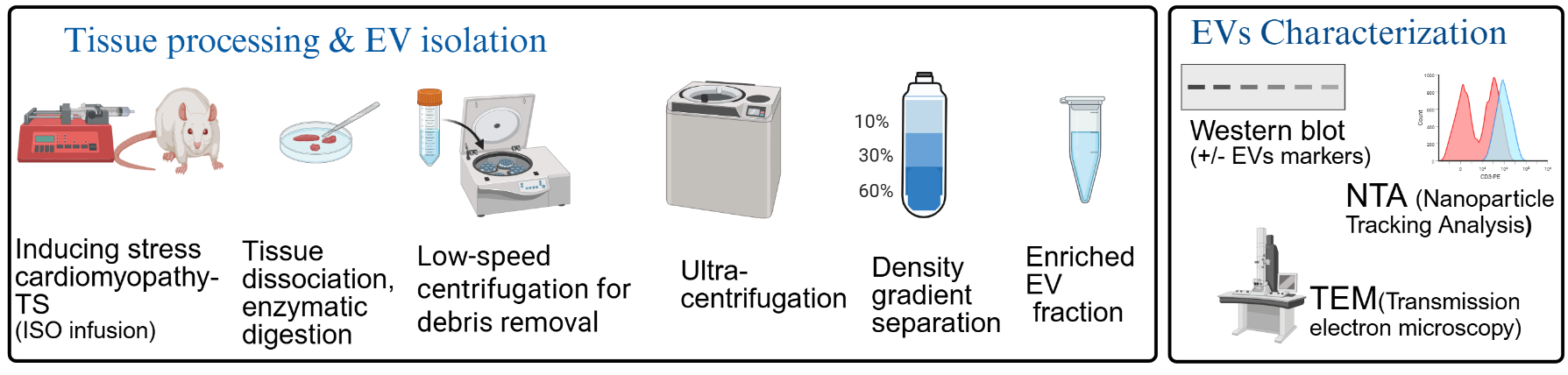


**Figure S2. Nanoparticle tracking analysis (NTA)** **characterization of EVs**

**(A)** Particle-to-protein ratio of extracellular vesicles derived from apical (A-EVs) and basal (B-EVs) myocardial regions measured by nanoparticle tracking analysis (NTA). Each dot represents an independent biological sample obtained from baseline or 24 h hearts (n = 12 per group). Data are shown as mean ± SEM. Comparable particle-to-protein ratios indicate similar EV yield between groups. **(B)** Size distribution of EVs assessed by nanoparticle tracking analysis (NTA). EVs derived from apex and base regions at baseline and 24 h display size distributions with a peak in the range of ~150–180 nm. Curves represent size distributions based on median particle size (X50) and particle concentration values.

**A)**


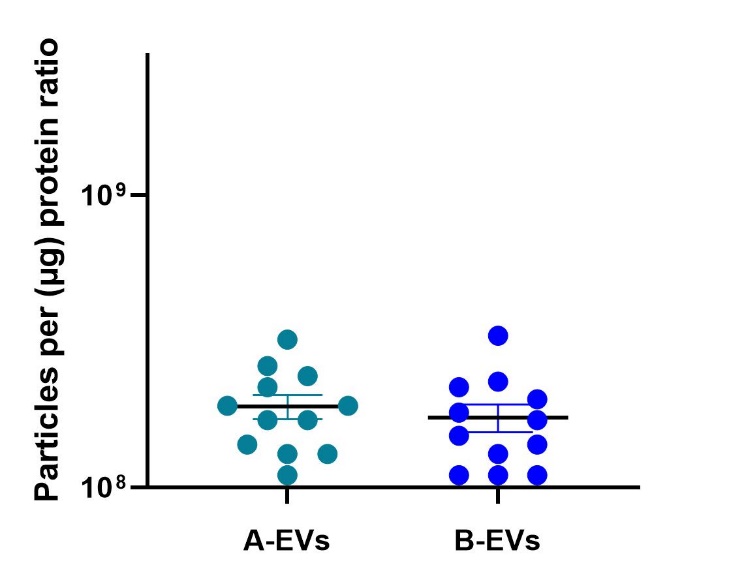


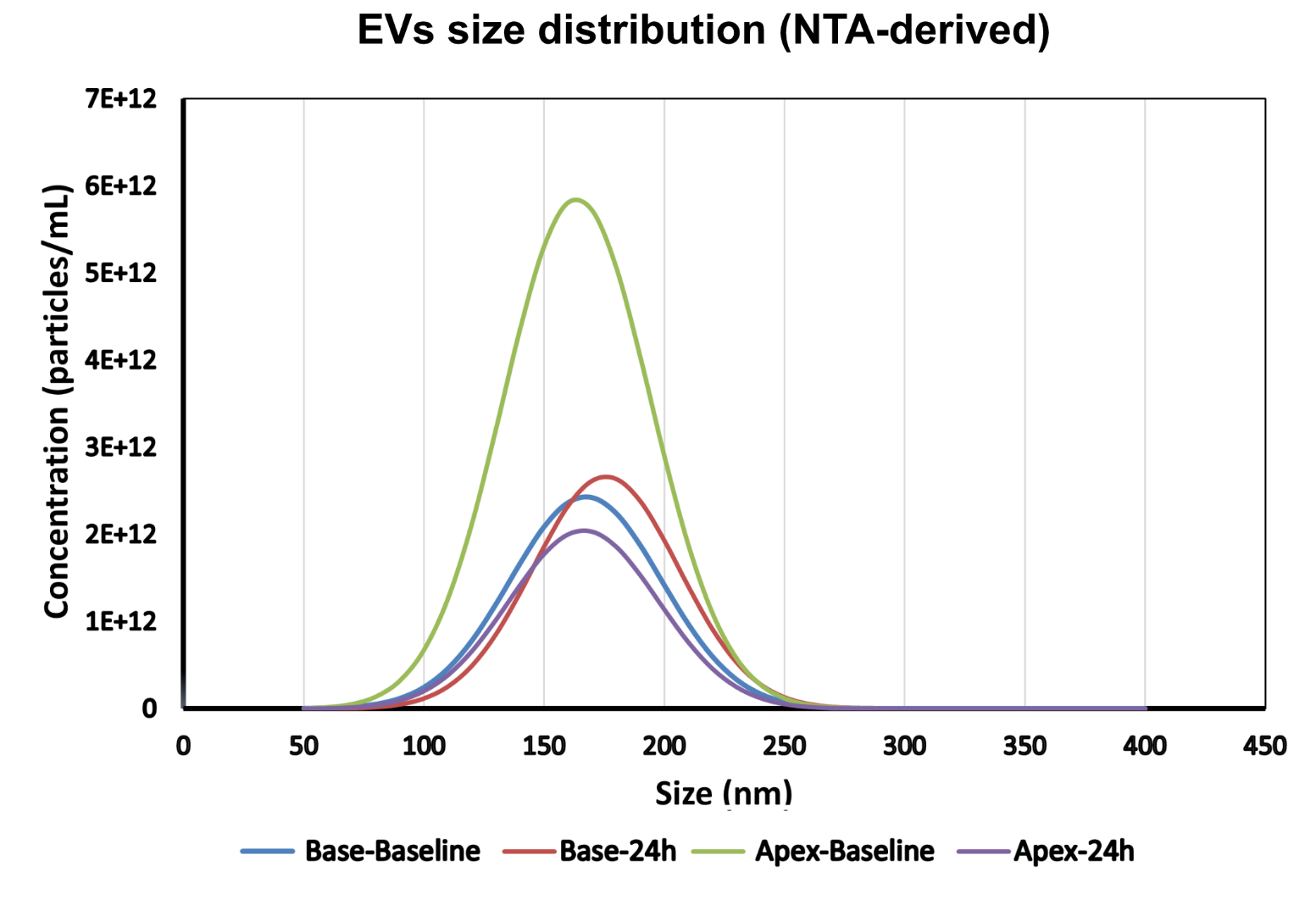
**B)**

**Figure S3.** **Transmission electron microscopy (TEM) characterization of apex-derived EVs collected after 24 hours induced stress**

Images show vesicles with typical round morphology and bilayer structure at increasing magnifications (scale bars: 1 μm, 500 nm, 200 nm).


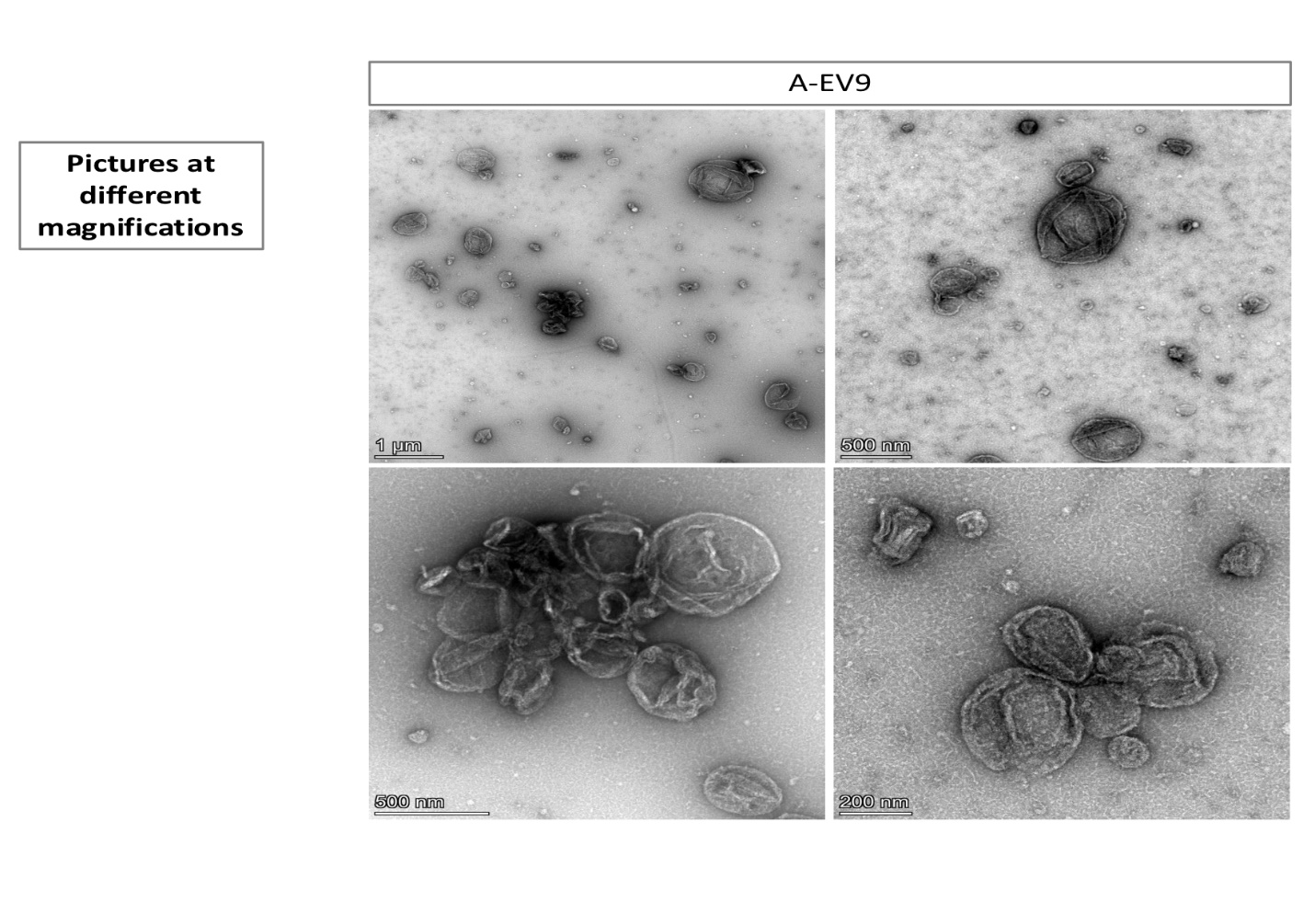


**Figure S4. Galectin-3 expression in heart tissue** **after catecholamine-induced stress in rats**

**(A)** Schematic experimental design for induction of catecholamine stress. Male rats were administered intravenous isoprenaline (ISO, 1 mg/kg, 15 minutes) to induce transient regional wall motion abnormalities. Hearts were collected 24 hours post-induction and dissected into apex and base regions. Baseline hearts were collected without prior ISO infusion. **(B)** Representative western blot analysis of Gal-3 levels and western blot quantification in 24 hours heart tissue with and without ISO infusion. **(C)** Gal-3 mRNA expression 24 hours after ISO infusion. n=2-5 per group in panel B and C. Statistical analyses were performed using one-way ANOVA. Data are presented as mean ± SEM: **p<0.01; ***p<0.001; ****p<0. 0001. ns= not significant.

**
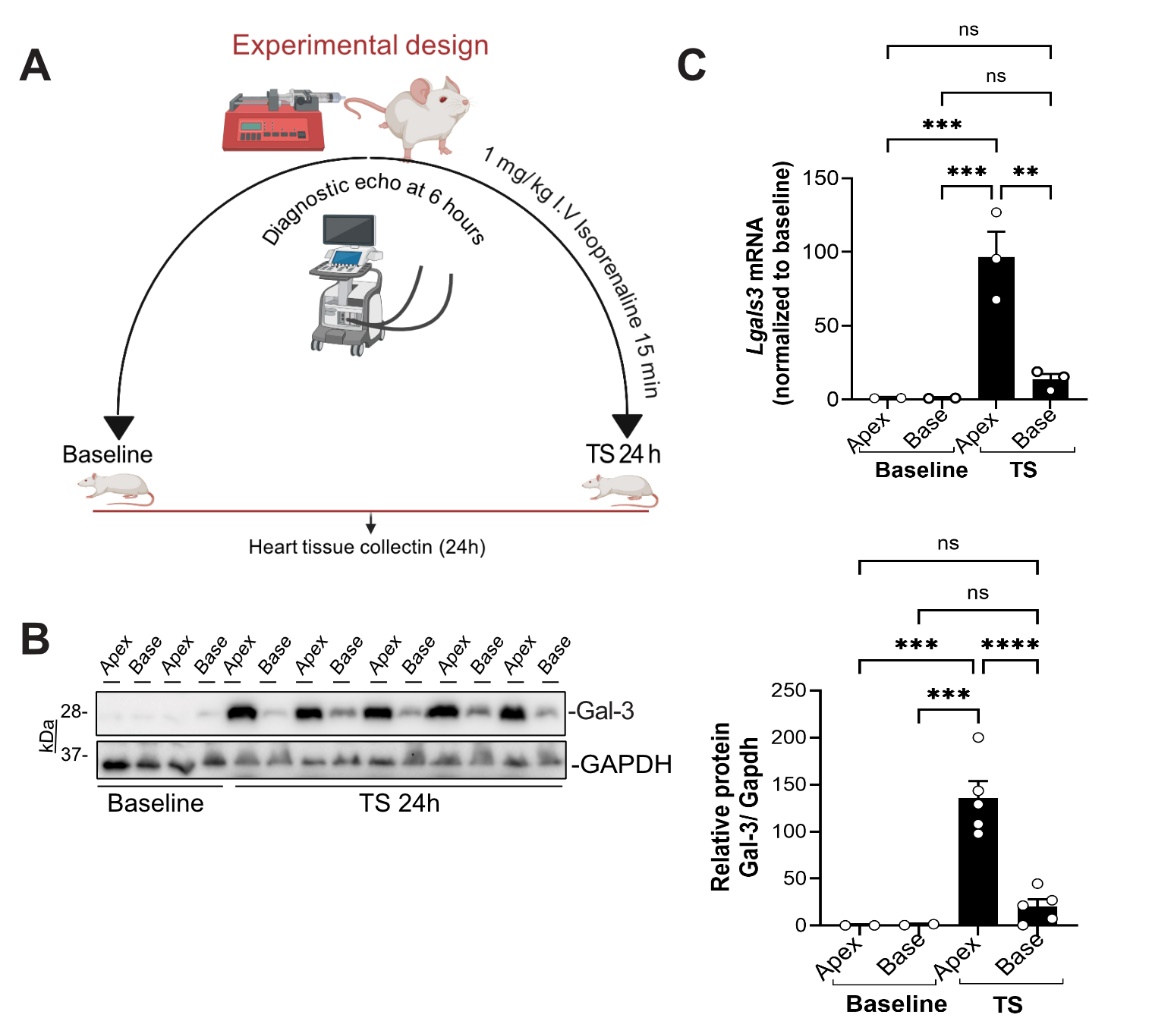
**

**Supplementary Table S1. PCR primers and corresponding gene accession numbers (Rattus norvegicus)**

| **Accession (RefSeq)** | **Gene name** | **Primer** | **Sequence (5'->3')** |
| --- | --- | --- | --- |
| NM_031832 | *Lgals3* | F | CAGTGCTCCTGGAGGCTATC |
|  | *Lgals3* | R | ATTGAAGCGGGGGTTAAAGT |
| NM_012854 | *Il-10* | F | CAAGGAGCATTTGAATTCCC |
|  | *Il-10* | R | GGCCTTGTAGACACCTTGGTC |
| NM_001415012 | *Nfkb1* | F | GTGCAGAAAGAAGACATTGAGGTG |
|  | *Nfkb1* | R | AGGCTAGGGTCAGCGTATGG |
| NM_012675 | *Tnfalpha* | F | GGCAGCCTTGTCCCTTGAAGAG |
|  | *Tnfalpha* | R | GTAGCCCACGTCGTAGCAAACC |
| NM_020081 | *Cd86* | F | TGCTCATCTAAGCAAGGATACCCG |
|  | *Cd86* | R | CGACTCGTCAACACCACTGTCCTG |
| NM_001006970 | *Uqcrc2 cxlll* | F | GGCTAATCCCTTGTACTGTCCT |
|  | *Uqcrc2 cxlll* | R | AGCCATTCTTGCACTTGTGA |
| NM_031347 | *Ppargc1a* | F | TACAACAATGAGCCCGCGAA |
|  | *Ppargc1a* | R | TGTGAGAACCGCTAGCAAGT |
| NM_012819 | *Acadl* | F | AAAGGTCTGGGAGTGATTGGA |
|  | *Acadl* | R | ACGAGATCACTTAACCAGCCA |
| NM_012880 | *Sod3* | F | CTTGACCTGGTTGAGAAGATAG |
|  | *Sod3* | R | GATCTGTGGCTGATCGG |
| NM_030826 | *Gpx1* | F | TGAGAAGTGCGAGGTGAATG |
|  | *Gpx1* | R | AACACCGTCTGGACCTACCA |
| NM_017008 | *Gapdh* | F | GGCAAGTTCAACGGCACAG |
|  | *Gapdh* | R | CGCCAGTAGACTCCACGAC |

**Supplementary Table S2. Top 30 EV enriched apical myocardial proteins**

| **Accession** | **Gene Symbol** | **Mean Baseline** | **Mean TS 24h** | **Foldchange** | **Log2FC** |
| --- | --- | --- | --- | --- | --- |
| P08721 | Spp1 | 0,06325 | 6,82025 | 107,8300395 | 6,752615 |
| P17977 | Ctsg | 0,167166667 | 4,939333333 | 29,54735793 | 4,884957 |
| Q6P7C7 | Gpnmb | 0,28825 | 6,293 | 21,83174328 | 4,448355 |
| O55006 | Rob1 | 0,2215 | 4,791333333 | 21,63130173 | 4,435049 |
| P50116 | S100a9 | 0,174 | 3,714 | 21,34482759 | 4,415815 |
| P00697 | Lyz1 | 0,2625 | 4,5445 | 17,31238095 | 4,113732 |
| P57756 | Fcn2 | 0,2855 | 4,699833333 | 16,46176299 | 4,041047 |
| P06238 | A2m | 0,557 | 9,049 | 16,2459605 | 4,022009 |
| Q9ERB4 | Vcan | 0,252 | 4,0145 | 15,93055556 | 3,993725 |
| P20961 | Serpine1 | 0,293833333 | 4,0905 | 13,92115712 | 3,799207 |
| P50115 | S100a8 | 0,221666667 | 3,057666667 | 13,79398496 | 3,785967 |
| P70553 | Slc11a1 | 0,2975 | 4,061 | 13,65042017 | 3,770873 |
| P35952 | Ldlr | 0,4255 | 5,360666667 | 12,59851156 | 3,655181 |
| Q91ZN1 | Coro1a | 0,382 | 4,601833333 | 12,04668412 | 3,590564 |
| P26051 | Cd44 | 0,428833333 | 5,110833333 | 11,91799456 | 3,57507 |
| P47819 | Gfap | 0,7155 | 8,523 | 11,91194969 | 3,574338 |
| Q07257 | Tgfb2 | 0,383833333 | 4,509666667 | 11,74902301 | 3,554469 |
| P04157 | Ptprc | 0,466 | 5,354 | 11,48927039 | 3,522215 |
| Q71LX6 | Xirp2 | 0,494833333 | 5,4135 | 10,94004715 | 3,451547 |
| Q9WV57 | Mpeg1 | 0,4345 | 4,7265 | 10,87802071 | 3,443344 |
| Q62737 | Cyba | 0,544 | 5,751 | 10,57169118 | 3,402134 |
| Q9R1T3 | Ctsz | 0,495 | 4,964166667 | 10,02861953 | 3,326051 |
| Q9R1E9 | Ccn2 | 0,3505 | 3,455333333 | 9,85829767 | 3,301339 |
| P05942 | S100a4 | 0,4485 | 4,1325 | 9,214046823 | 3,203835 |
| P20759 | Ighg1 | 0,246333333 | 2,188833333 | 8,885656292 | 3,151478 |
| Q9WVR6 | Slc7a8 | 0,599333333 | 5,162333333 | 8,613459399 | 3,106593 |
| P08932 | Knt2 | 0,4165 | 3,5795 | 8,594237695 | 3,10337 |
| **P08699** | **Lgals3** | **0,531666667** | **4,355666667** | **8,192476489** | **3,0343** |
| Q62715 | Defa | 0,453 | 3,5335 | 7,800220751 | 2,963515 |
| P08430 | Ugt1a6 | 0,46825 | 3,557 | 7,596369461 | 2,92531 |

**Uncropped and Cropped Western blot bands**


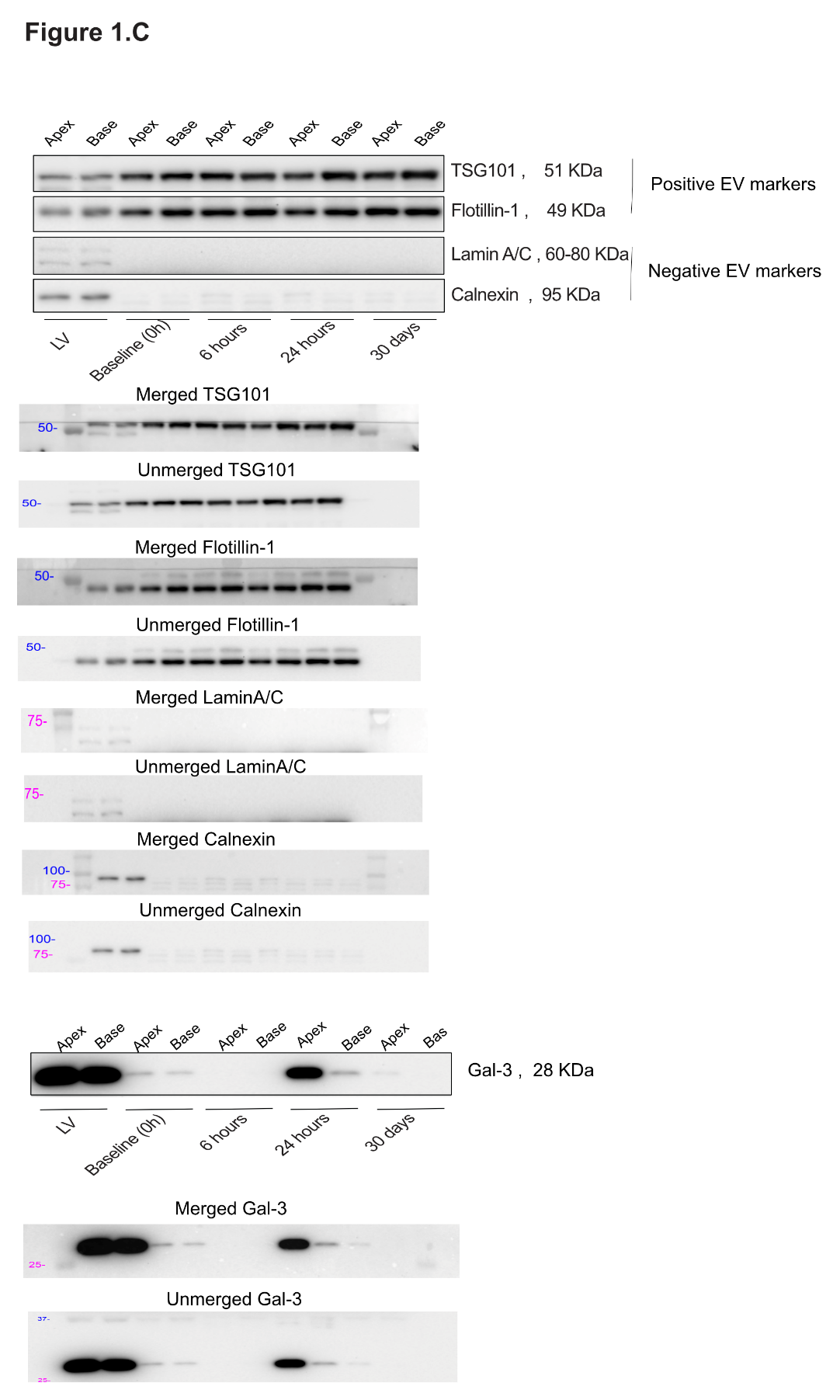


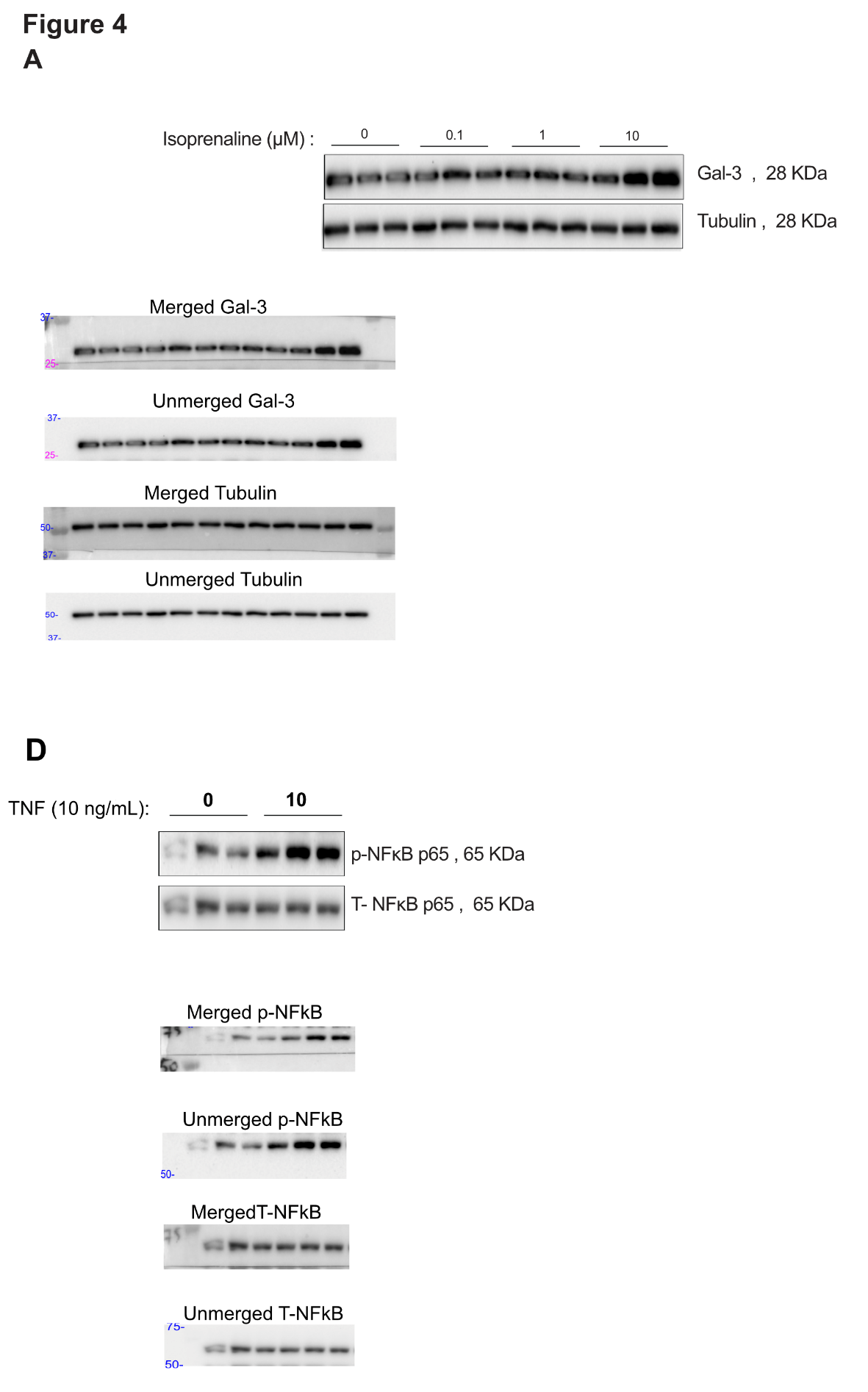


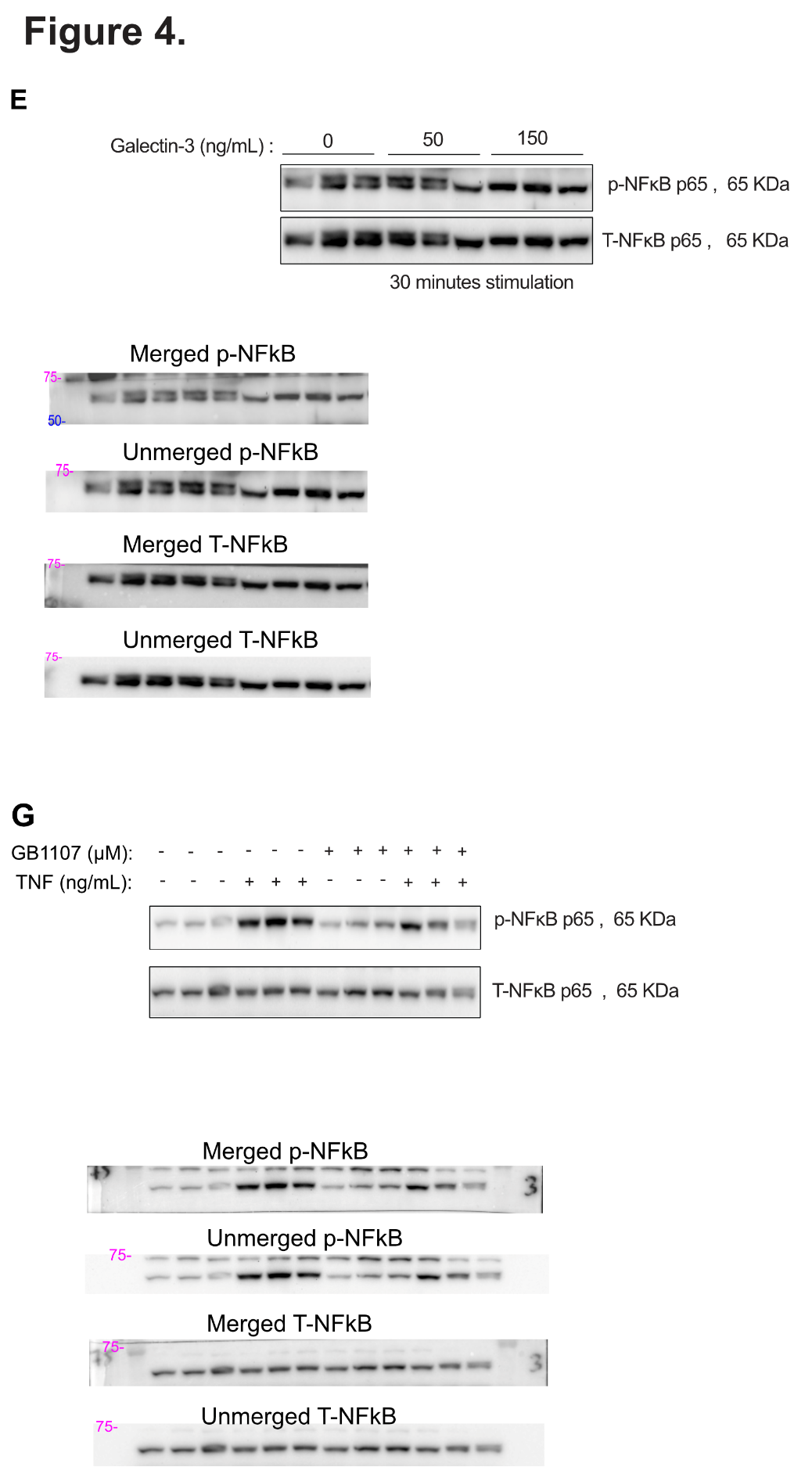


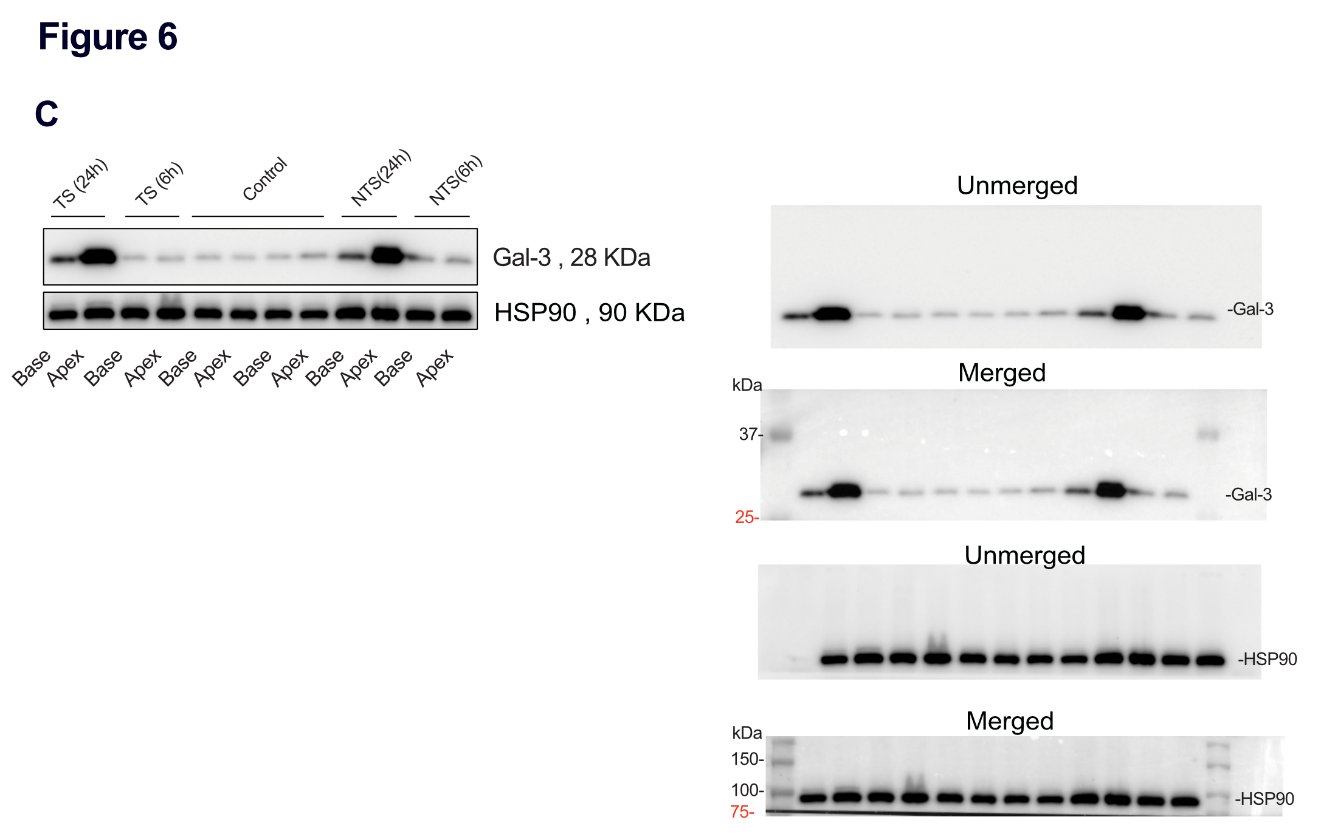


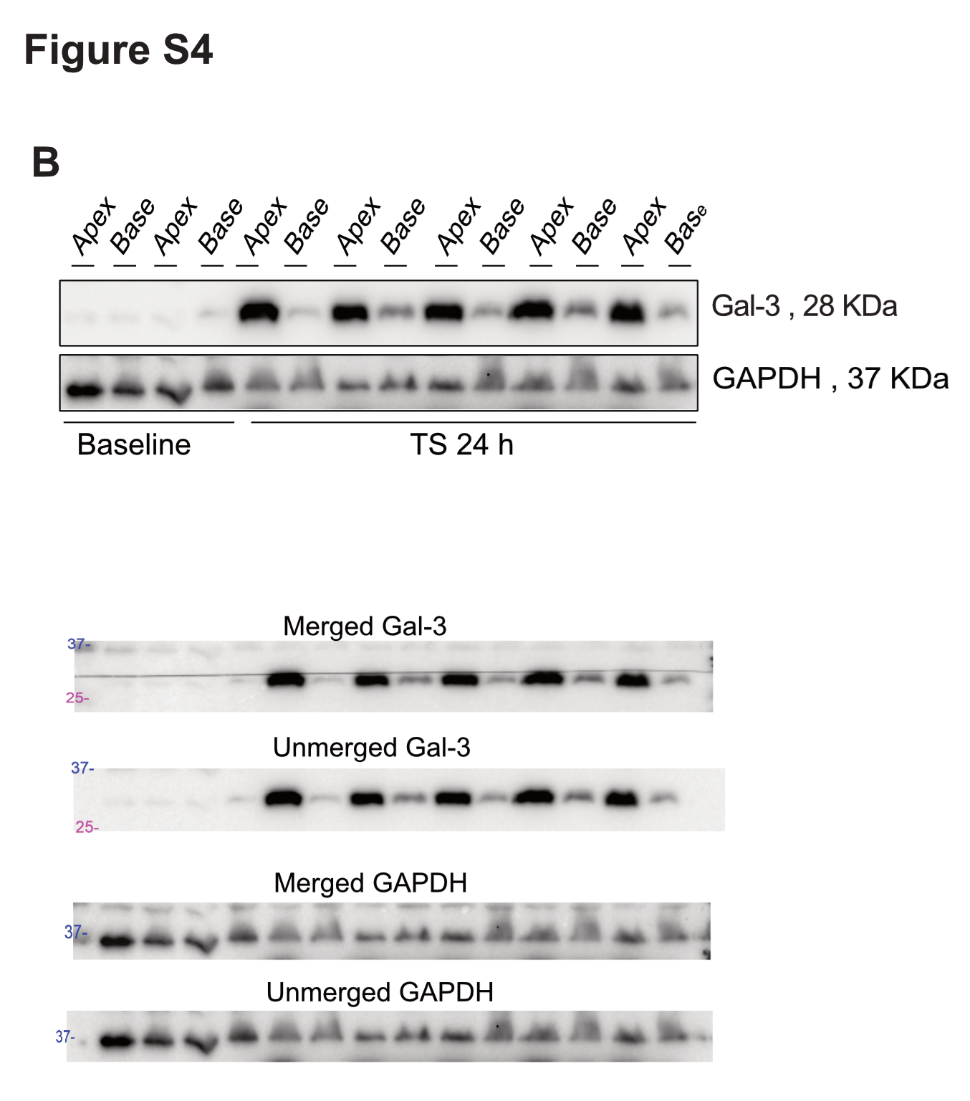

Supplement: Supplementary file 1 — Supplementary Material 1. Supplementary Appendix. [file 10020_2026_1472_MOESM1_ESM.zip › Kalani, et al-Supplementary material.docx]
